# Long‐Term Stable Subdural Recordings Enabled by Fibrosis‐Resistant Hydrogel‐Integrated µECoG Arrays

**DOI:** 10.1002/advs.202515453

**Published:** 2025-10-08

**Authors:** Lin Chen, Hao Zhong, Linghao Wang, Liju Xu, Wanying Fan, Yongpeng Zhao, Huiling Zhang, Yang Shen, Kai Wu, Xin Fu, Jingxiang Guo, Ke Li, Dong Qiu, Ting Wu

**Affiliations:** ^1^ Beijing Institute for Brain Research Chinese Academy of Medical Sciences & Peking Union Medical College Beijing 102206 China; ^2^ Chinese Institute for Brain Research Beijing 102206 China; ^3^ Beijing National Laboratory for Molecular Sciences, Laboratory of Polymer Physics and Chemistry CAS Research/Education Center for Excellence in Molecular Sciences Institute of Chemistry Chinese Academy of Sciences Beijing 100190 China; ^4^ University of Chinese Academy of Sciences Beijing 100049 China; ^5^ School of Basic Medical Sciences Capital Medical University Beijing 100069 China

**Keywords:** anti‐fibrotic, hydrogel‐integrated bioelectronics, micro‐electrocorticography arrays, neural interfaces, subdural implantation

## Abstract

The long‐term performance of microscale subdural arrays is often compromised by adverse tissue responses, leading to increased electrochemical impedance and signal deterioration during chronic applications. Here, the study presents an adhesive hydrogel‐integrated micro‐electrocorticography (aGel‐µECoG) array that improves mechanical compatibility and adhesion to brain tissue, effectively mitigating tissue responses and enabling stable, high‐performance electrical communication over days to months. The hydrogel consists of a hydrophilic polyvinyl alcohol and hydrophobic poly3‐(trimethoxysilyl) propyl methacrylate heteronetwork, serving as a biological and mechanical bridging to enable seamless, anti‐fibrotic, long‐term integration with brain tissue. The aGel‐µECoG achieves robust tissue adhesion (25.2 ± 3.8 kPa) with reversible and safe removal, enabling sutureless implantation. With an ultrathin 10‐µm ionic conductive hydrogel coating, the array exhibits high electrical fidelity, effectively preserving fine‐grained sub‐millimeter spatial resolution and maintaining unattenuated signal strength for functional cortical mapping. Compared to conventional µECoG arrays, aGel‐µECoG exhibits a 20‐fold reduction in acute‐phase impedance increases, attributed to significantly reduced neuroinflammation and fibrotic tissue formation, as confirmed by histological analyses. Long‐term recordings further reveal that aGel‐µECoG maintained 94.8% of the signal‐to‐noise ratio for steady‐state visually evoked potentials over 16 weeks, whereas uncoated µECoG arrays declined to 69.5%. These findings establish aGel‐µECoG as a durable and high‐performance neural interface.

## Graphical abstract

An adhesive hydrogel‐integrated micro‐electrocorticography (aGel‐µECoG) array is developed to ensure stable neural recordings by minimizing neuroinflammation and fibrosis during implantation. The hydrogel serves as a biological and mechanical bridge, enabling bioadhesive, reversible, anti‐fibrotic integration with brain tissue. The platform demonstrates high electrical fidelity, negligible adverse tissue responses, and stable recordings over four months, offering a promising solution for long‐term neural interfacing applications.

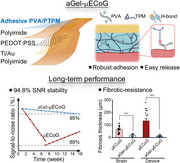

## 1 Introduction

**Figure 1:**
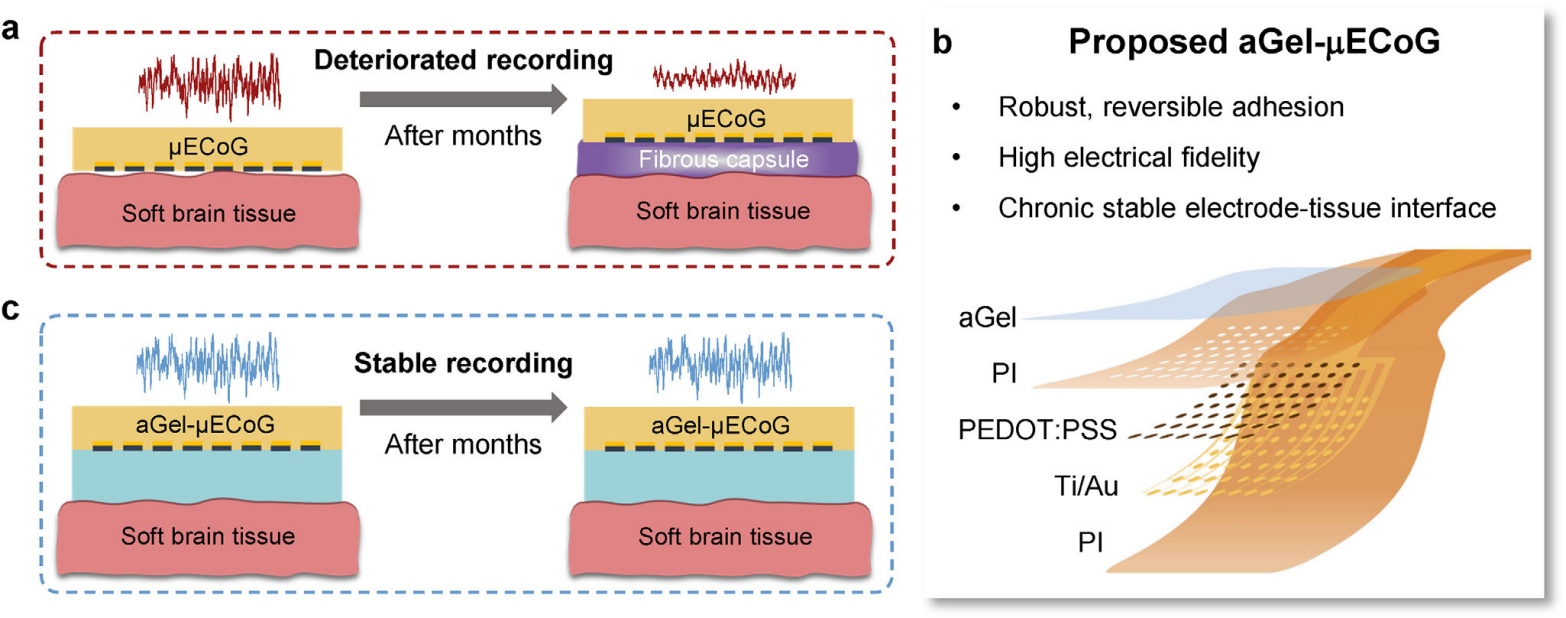
Conceptual and schematic overview of aGel‐µECoG for high‐performance chronic subdural signal recording. a) Schematic illustrations of long‐term in vivo neural signal recording with µECoG, highlighting the presence of observable fibrous capsule formation and deteriorated recording. b) Key features of the proposed ideal hydrogel‐integrated neural interface and an expanded schematic illustration detailing the layered structure of the proposed aGel‐µECoG. c) Schematic illustrations of long‐term in vivo neural signal recording with aGel‐µECoG, highlighting the absence of observable fibrous capsule formation at the electrode‐tissue interface, showing stable cortical recordings for months.

ECoG arrays are increasingly used in functional cortical mapping,^[^ ^1^, ^2^ ^]^ epilepsy monitoring,^[^ ^3^, ^4^ ^]^ and brain‐computer interfaces (BCIs).^[^ ^5^, ^6^, ^7^, ^8^ ^]^ Subdural ECoG arrays offer a favorable compromise between invasiveness and high spatiotemporal resolution,^[^ ^9^, ^10^, ^11^ ^]^ making them valuable tools for high‐performance neural interfacing. However, clinically available macro‐ and meso‐scale ECoG arrays suffer from limited spatial resolution (3.5–5 mm) and non‐conformal contact with the brain's irregular surface.^[^ ^12^, ^13^ ^]^ Flexible thin‐film µECoG devices have emerged as promising alternatives.^[^ ^14^, ^15^ ^]^ Constructed from ultrathin substrates of flexible plastics^[^ ^16^, ^17^, ^18^, ^19^, ^20^ ^]^ or soft elastomers,^[^ ^21^, ^22^, ^23^, ^24^ ^]^ µECoG arrays establish conformal contact with the brain, enhance signal coupling, reduce motion artifacts, and minimize tissue irritation. Second, their small‐diameter electrodes and high‐density configurations, enabled by advanced microfabrication, provide superior spatiotemporal resolution and signal‐to‐noise ratio (SNR) in both animal models^[^ ^16^, ^25^, ^26^ ^]^ and human studies.^[^ ^26^, ^27^, ^28^, ^29^ ^]^ However, the long‐term reliability of µECoG devices is compromised by adverse tissue responses at the electrode‐tissue interface. Despite their flexibility, the substantial mechanical modulus mismatch between µECoG arrays (Young's modulus *E* > 1 GPa) and soft brain tissue (*E* < 30 kPa)^[^ ^21^, ^22^, ^30^ ^]^ induces chronic inflammatory responses, including glial cell activation, progressive gliosis, and fibrotic capsule formation.^[^ ^31^, ^32^, ^33^ ^]^ These adverse tissue responses are believed to cause impedance fluctuations, deteriorate signal quality, and potentially lead to device dysfunction (**Figure** **1**a).^[^ ^9^, ^33^, ^34^, ^35^ ^]^ Additionally, because µECoG devices lack intrinsic adhesive properties, microscale displacement caused by bodily motion and cerebrospinal fluid (CSF) dynamics could introduce instability in high spatial resolution recording.^[^ ^36^, ^37^ ^]^ External anchoring methods, such as sutures or adhesive glues, are required but may exacerbate biocompatibility concerns.

Recent advances in hydrogel‐integrated bioelectronics offer promising strategies to address critical challenges, including the improvement of mechanical modulus, conductivity, durability, and adhesiveness, which are essential for reliable and long‐term biointerfacing applications.^[^ ^38^ ^]^ Approaches such as bio‐inspired adhesive chemistries, hierarchical structural design, and advanced crosslinking mechanisms have demonstrated significant progress in enhancing hydrogel performance across various biointerfaces, including cardiac, nerve, skin, subcutaneous, and neuromuscular systems (Table S1, Supporting Information).^[^ ^39^, ^40^, ^41^, ^42^, ^43^, ^44^, ^45^ ^]^ However, translating these successes into neural interfaces requires more than simple adaptation because of the unique physiological and mechanical complexities of the brain tissue. Emerging neural‐specific designs, such as highly conductive carrageenan‐interpenetrated PDA‐polyacryl‐amide hydrogel‐integrated ECoG arrays, exhibit immune‐invasive capabilities and provide reliable signal transduction.^[^ ^46^ ^]^ Similarly, non‐deformable hydrogel patches have been shown to enhance the recording stability of µECoG arrays for a week, while minimizing fibrotic tissue responses.^[^ ^36^ ^]^ Additionally, catechol‐conjugated alginate hydrogel cortex adhesive sensors have demonstrated stable epidural recording during transcranial focused ultrasound neurostimulation for nearly half a year.^[^ ^37^ ^]^ Despite these advances, these systems mainly rely on chemical modifications, such as catechols^[^ ^37^, ^46^ ^]^ and N‐hydroxysuccinimide^[^ ^36^ ^]^ for tissue adhesion. While these approaches ensure robust and permanent adhesion, they raise concerns about chemical contamination and potential damage during implant removal.^[^ ^47^ ^]^ In contrast, hydrogel‐integrated arrays that rely solely on physical hydrogen bonding mechanisms offer reversible attachment and mitigate chemical risks.^[^ ^32^, ^47^ ^]^ However, their long‐term subdural implantation remains underexplored, particularly in terms of the evaluation of signal stability during the progression of neuroinflammation and fibrotic tissue formation. Moreover, previous studies have often overlooked the spatial decoupling effect by interposing a functional hydrogel layer between the electrodes and neural tissue, which introduces signal attenuation governed by the classic volume conduction model.^[^ ^48^ ^]^ Suboptimal parameter designs for hydrogel thickness and conductivity may compromise the electrical fidelity of low‐amplitude neural recordings. Bridging these gaps requires innovative approaches to optimize material properties, adhesion mechanisms, and long‐term in vivo biocompatibility and stability evaluation.

In this study, we present an aGel‐µECoG neural interface that mitigates tissue responses and ensures long‐term signal stability (Figure 1b,c). We propose that such hydrogel‐integrated neural interfaces should meet the following requirements: i) robust tissue adhesion with reversible bonding to prevent cortical damage during explantation; ii) high electrical fidelity coating with spatiotemporal resolution comparable to that of its host device; and iii) minimized neuroinflammation and fibrotic capsule formation for sustained performance in vivo. To meet these requirements, we rationally optimized the aGel‐µECoG. The aGel‐µECoG consisted of a previously reported hydrophilic polyvinyl alcohol (PVA) and hydrophobic poly3‐(trimethoxysilyl) propyl methacrylate (PTPM) heteronetwork^[^ ^49^ ^]^ as a hydrogel adhesive, a high‐density polyimide (PI)‐based µECoG array for enhanced resolution, and an electroplated poly(3,4‐ethylenedioxythiophene):polystyrene sulfonate (PEDOT:PSS) layer for reducing the electrochemical impedance (Figure 1b). To ensure robust physical adhesion, aGel‐µECoG was dry‐cross‐linked to biological tissue, enabling tight adherence for long‐term implantation. We established the design principles for the adhesive hydrogel (aGel) interfacial layer and validated its high electrical interfacing efficacy using a 1024‐channel high‐resolution design. A long‐term in vivo histological analysis was conducted using a disk‐shaped variant following the ISO 10993–6 standards^[^ ^50^ ^]^ to evaluate long‐term in vivo biocompatibility. We demonstrated long‐term impedance and signal stability using a 64‐channel aGel‐µECoG over 16 weeks. We envision that the aGel‐µECoG platform will offer significant promise in advancing the implementation of reliable and long‐term neural interfaces.

## 2 Results

### 2.1 aGel Preparation and Characterizations

The aGel was engineered by combining PVA and PTPM to form a unique hydrophilic/hydrophobic heteronetwork structure (Figure S1, Supporting Information).^[^ ^49^ ^]^ PVA was chosen as the primary hydrogel matrix because of its biocompatibility and bioadhesiveness.^[^ ^51^, ^52^ ^]^ The incorporation of hydrophobic PTPM restricted excessive swelling of the PVA network. This design ensured structural stability while maintaining favorable adhesion properties. The successful synthesis of PVA/PTPM was confirmed by FTIR spectroscopy (Figure S2, Supporting Information). The ratio of PVA to PTPM significantly influences the hydrogel's functional properties. To identify the optimal composition, we evaluated three ratios (17:1, 7:1, and 3.5:1) based on key performance metrics, including ionic conductivity, tissue adhesion, swelling resistance, and transparency (Figure S3, Supporting Information). Among these, the 7:1 ratio was chosen for aGel in this work, which exhibited superior performance across multiple functionalities.

**Figure 2:**
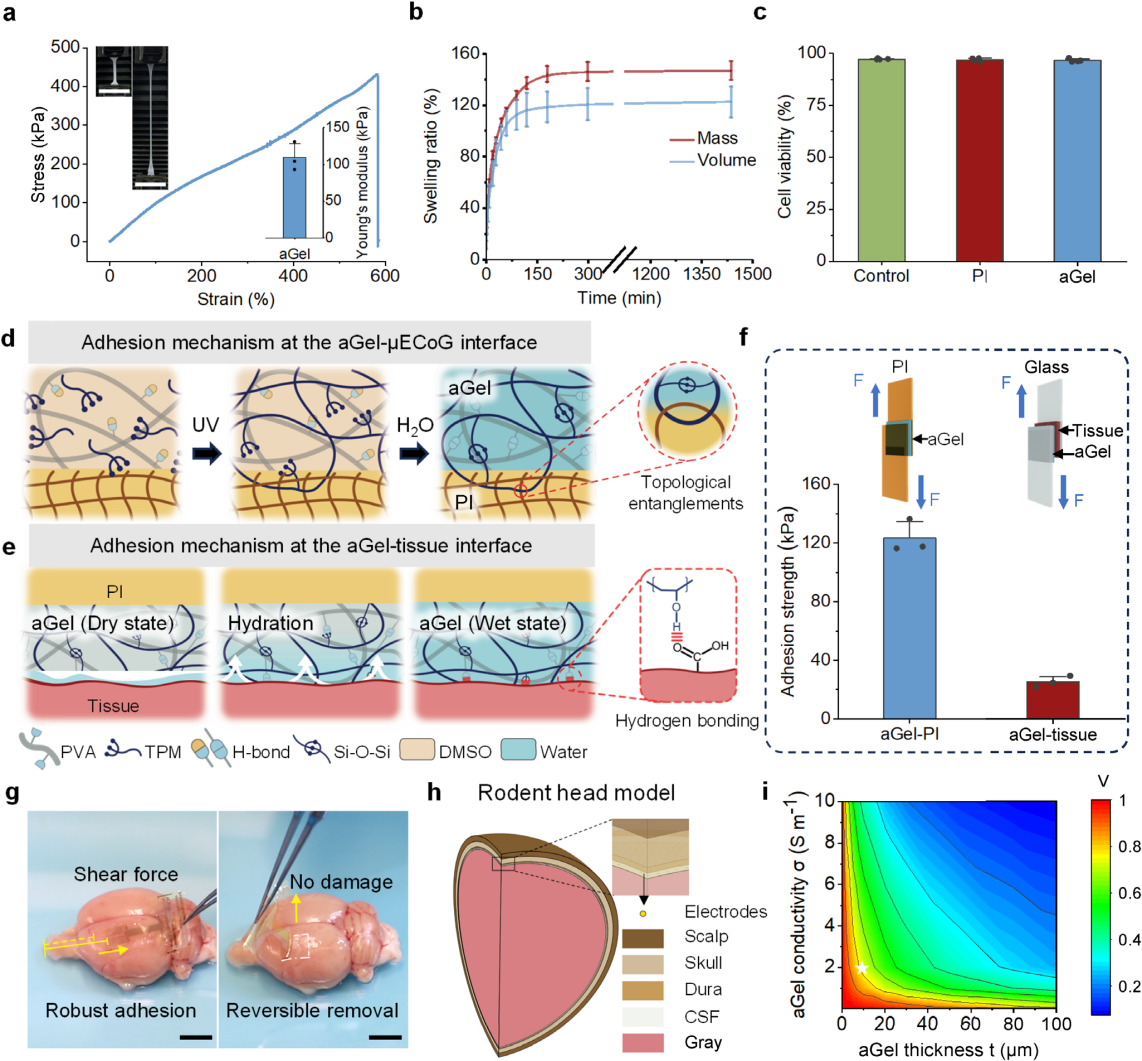
Key characterization and design optimization of aGel‐µECoG for enhanced functionality. a) Tensile stress‐strain curve of aGel, with Young's modulus derived from the curve (inset, bottom right; n = 3). Inset (top left): representative images of an aGel specimen before and after elongation, without fracture. Scale bar, 4 cm. b) Mass and volume swelling ratios of the aGel film immersed in 1X PBS over time (n = 3). c) Quantitative assessment of calcein‐AM/propidium iodide staining results following a 24‐hour incubation of NH3T3 cells with culture medium, 100% PI extract, or 100% aGel extract (n = 4). One‐way ANOVA revealed no significant intergroup differences. d) Adhesion mechanism at the aGel‐µECoG interface. e) Adhesion mechanism at the aGel‐tissue interface. f) Adhesion strengths at the aGel‐PI and aGel‐tissue interfaces, with insets illustrating the lap shear test configurations for each interface (n = 3). g) Images demonstrating the conformal and robust bioadhesive performance of the aGel‐µECoG array on ex vivo cortical tissues. h) Multi‐layered rodent head model for FE simulation. i) Normalized iso‐potential map showing the effects of varying *t* (0–100 µm) and σ (0.01–10 S m^−1^). The potential (*V*) was normalized to the maximum potential obtained at the geometric origin *d*_0_ under aGel‐free conditions. The white star indicates the optimal design parameter chosen to achieve high electrical fidelity (*t* ≈10 µm, σ ≈2 S m^−1^). Data are presented as mean ± standard deviation (SD).

Due to the incorporation of hydrophobic PTPM, the dry aGel film exhibited a larger water contact angle than the pure PVA film, while remaining hydrophilic overall (Figure S4a,c, Supporting Information). The water contact angle measurements further showed that the hydrated aGel achieved intermediate surface hydrophilicity between that of PI and hydrated PVA (Figure S4b,d, Supporting Information). Tensile tests of the aGel film demonstrated exceptional mechanical properties, including high stretchability (tensile strain: 555.1 ± 25.8%) and toughness (fracture energy: 31.4 ± 3.3 kJ m^−2^) (**Figure** **2**a). The aGel exhibited an ultrasoft Young's modulus of 109.4 ± 19.5 kPa, effectively bridging the mechanical mismatch between the soft brain tissue and the rigid polyimide substrate (Figure 2a).

A significant challenge for hydrogel interfaces during long‐term implantation is buckling or wrinkling caused by excessive swelling, which can lead to delamination from biological tissues or bioelectronics.^[^ ^49^ ^]^ Swelling tests revealed stable mass and volume swelling ratios (143.8 ± 6.3% and 118.1 ± 11.9%, respectively) after 180 min in phosphate‐buffered saline (PBS, 1X, pH 7.4; Figure 2b; Figure S5, Supporting Information), indicating the stability of the aGel film in physiological environment. To further demonstrate the long‐term stability of aGel, we immersed the film in PBS for one month and observed no significant changes in mass or volume (Figure S5, Supporting Information). The swelling resistance of the aGel layer prevents surface delamination and ensures structural integrity during long‐term implantation.

Next, we assessed the in vitro biocompatibility of aGel films. Since non‐specific protein adsorption is a key factor in fibrosis initiation,^[^ ^43^, ^53^ ^]^ we challenged PVA, aGel, and PI with bovine serum albumin (BSA) solution. We found that both aGel and PI demonstrated excellent resistance to protein fouling compared to PVA (*p* < 0.01, Figure S6, Supporting Information). Next, we conducted cytotoxicity assay using NIH3T3 cells (a mouse embryonic fibroblast cell line) and found no significant toxicity for either aGel or PI extracts compared to the negative control medium (Figure 2c; Figure S7, Supporting Information). Further, we assessed the ability of aGel and PI to support the adhesion and growth of NIH3T3 cells. The experimental results indicated that during the initial 24‐hour culture period, cell attachment density was low on both PI and aGel surfaces (Figure S8, Supporting Information). On day three, there was a significant decline in cell density on aGel surface (*p* < 0.05). Our results indicate that aGel has a low protein adsorption and weak capacity for cell adhesion and growth, highlighting its exceptional anti‐fouling performance.

In addition, the aGel exhibited high optical transparency across wavelengths of 400–800 nm (Figure S9, Supporting Information), enabling potential optogenetic applications. Its ionic conductivity (1.99 S m^−1^, Figure S10, Supporting Information) after soaking in PBS (1X) closely matched that of CSF, supporting its compatibility with electrophysiological applications.

### 2.2 aGel‐µECoG Preparation and Characterizations

To achieve high flexibility and biocompatibility, we fabricated 6 µm‐thick µECoG arrays using biocompatible PI as the substrate,^[^ ^54^ ^]^ with a 10 nm/200 nm Ti/Au metal layer for electrical conductivity (Figure S11, Supporting Information). The electrochemical performance of the 64/1024‐channel electrodes was further optimized by electroplating with PEDOT:PSS.

For seamless device integration, an aGel precursor was applied to the µECoG array using a scrape‐coating technique to ensure a uniform and precise layer thickness, followed by a two‐step in situ gelation process involving photopolymerization and solvent exchange (Figure 2d). The gelation kinetics were well controlled to prevent strain‐induced deformation or delamination between the aGel and µECoG devices. Impedance and cyclic voltammetry (CV) measurements showed negligible changes after coating, with no observable wrinkles on the PEDOT:PSS layer (Figures S20 and S26, Supporting Information). The heteronetwork structure of the aGel physically interlocked with the µECoG surface, providing robust adhesion, as confirmed by lap‐shear tests, which indicated an adhesion strength of 123.5 ± 11.2 kPa (Figure 2f). Figure S12 (Supporting Information) demonstrated that the aGel‐bonded PI films supported a weight of 500 g underwater, verifying their adhesiveness in wet environments. Cross‐sectional cryo‐scanning electron microscopy revealed seamless integration at the aGel‐PI interface, eliminating delamination or air gaps during chronic implantation (Figure S13, Supporting Information).

To address the adhesion challenges on slippery, wet biological tissues, we employed a dry cross‐linking mechanism. Upon contact with wet tissues, aGel‐µECoG in the dry state rapidly absorbed interfacial water through its hydrophilic PVA network. This process immediately forms strong hydrogen bonds with the tissue surface (Figure 2e). The PTPM network further enhanced the structural stability and biocompatibility, achieving an adhesion strength of 25.2 ± 3.8 kPa to biological tissues (Figure 2f; Figure S14a, Supporting Information). A 90‐degree peeling test revealed a low peeling energy (27.79 ± 2.24 Pa) for aGel‐µECoG (Figure S14b, Supporting Information). Ex vivo tests were performed on the rodent brain (Figure 2g; Movie S1, Supporting Information) and porcine heart tissue (Figure S15, Supporting Information), further confirmed that aGel‐µECoG achieved strong adhesion and conformal contact and ensured safe removal without causing damage to diverse tissues.

Although high‐density µECoG arrays are known for their superior signal quality and spatiotemporal resolution,^[^ ^16^, ^20^, ^25^ ^]^ integrating an aGel layer introduces design challenges owing to the increased source‐electrode distance, potentially compromising the fidelity of weak neural signals.^[^ ^19^ ^]^ To address this challenge, we systematically optimized the design parameters of the aGel layer using computational simulations. We constructed a five‐layer rodent head model based on previous studies,^[^ ^48^, ^55^ ^]^ including gray matter, CSF, dura mater, skull, and scalp, for finite element (FE) simulations (COMSOL Multiphysics V6.2; Figure 2h; Table S2, Supporting Information). A 16 × 16 aGel‐µECoG array, with 20 µm electrode diameters and 200 µm pitches, was placed subdurally in the rodent brain model in the simulations (Figure S16c, Supporting Information). Our results revealed trends consistent with theoretical volume conduction models. Specifically, both the thickness and conductivity of the aGel layer significantly influenced the signal propagation, with thicker or more conductive layers leading to reduced signal communication efficiency (Figure 2i; Figures S16–S19, Supporting Information). Integrating our findings, we identified an ultrathin aGel layer (≈10 µm thickness, ≈2 S m^−1^ conductivity) as design parameters for high electrical fidelity. The fabrication processes further confirmed the feasibility of achieving ultrathin aGel layers as thin as 10 µm, validating their suitability as operational windows for aGel‐µECoG arrays.

### 2.3 aGel‐µECoG Preserves Signal Fidelity in High‐Resolution Functional Cortical Mapping

**Figure 3:**
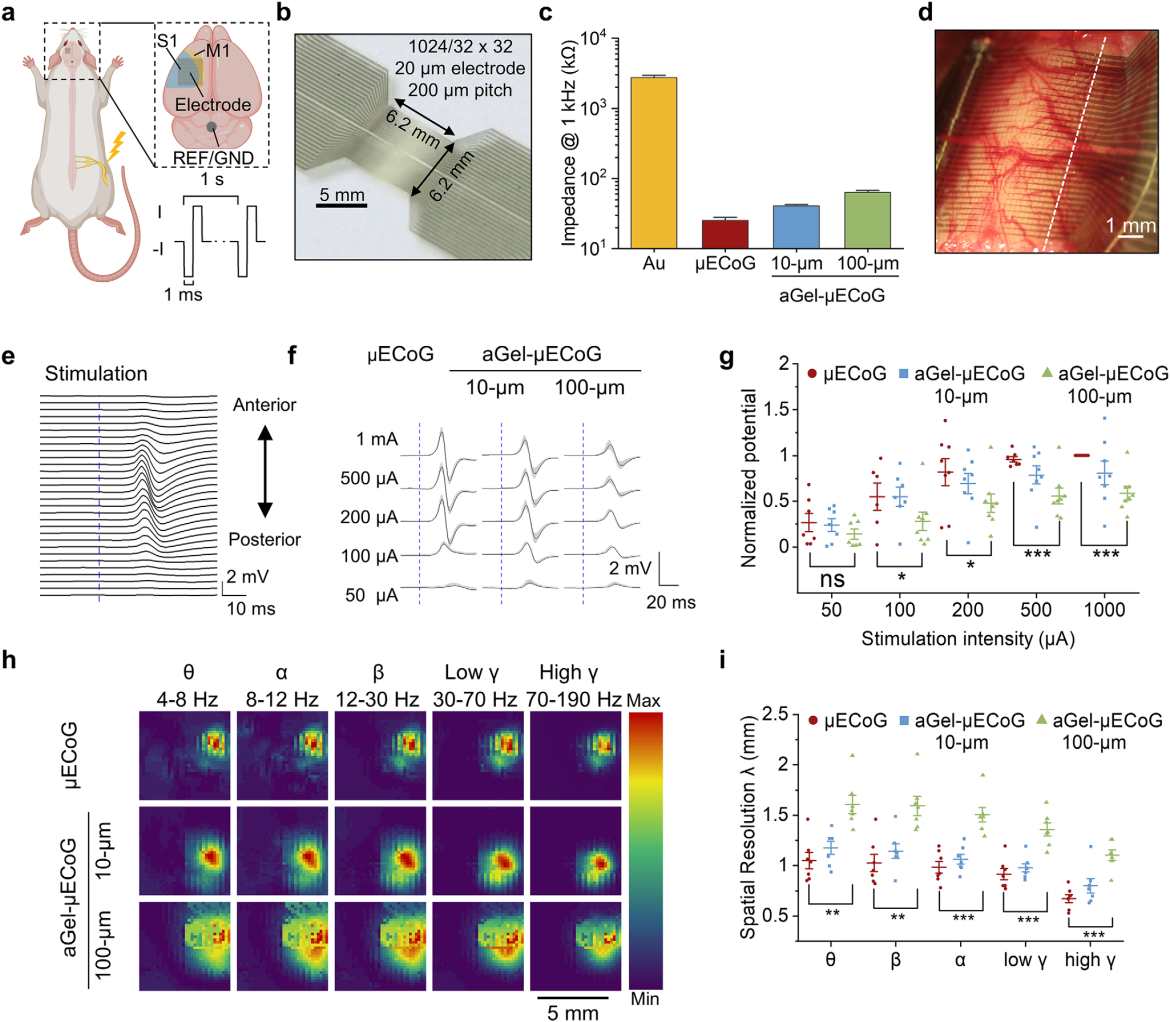
High‐resolution cortical mapping with 1024‐channel arrays. a) Cartoon diagram illustrating somatosensory cortex recordings during hindlimb electrical stimulation. b) Representative image of the 1024‐channel aGel‐µECoG array. c) Impedance at 1 kHz for devices with as‐fabricated bare Au electrodes, µECoG (electroplated with PEDOT:PSS), and aGel‐µECoG with coating thicknesses of 10 and 100 µm. Data were collected from more than 60 electrodes across three independent samples and are presented as the mean ± SD. d) Representative images showing placement of aGel‐µECoG on the rat cortex. During the experiment, each type of electrode was sequentially placed on the somatosensory cortex to cover comparable regions. e) SSEP signals recorded from the white dashed line indicate the column in (c) using a representative aGel‐µECoG during electrical stimulation. The blue dashed line indicates the onset of the electrical stimulation. f) Representative trial‐averaged SSEPs for three device types under varying stimulation intensities. g) Normalized SSEP potentials across varying stimulation intensities (50, 100, 200, 500, and 1000 µA) for three device types (sample sizes: µECoG, n = 7, 6, 8, 7, 8; 10‐µm aGel‐µECoG, n = 7, 7, 8, 8, 8; 100 µm aGel‐µECoG, n = 7, 8, 8, 8, 8). Statistical analysis was performed using an unpaired *t‐*test for a specific stimulation condition and two‐way ANOVA for comparisons among device types under all stimulation conditions. h) Bandpass‐filtered spatial maps of SNR at a stimulation intensity of 200 µA. i) Estimation of spatial resolution using the fitted parameter λ across frequency bands (n = 7 per group; exponential decay fit: y = a·exp(‐x/λ) + c). Data are presented as mean ± standard error of the mean (SEM), unless otherwise specified. ns, not significant; *p < 0.05, **p < 0.01, ***p < 0.001.

To experimentally evaluate the signal fidelity of the ultrathin aGel‐µECoG arrays, we performed functional cortical mapping in anesthetized rats using high‐density 1024‐channel devices (**Figure** **3**a). The arrays were fabricated on a 6‐µm‐thick PI substrate with 20 µm diameter electrodes spaced at a 200 µm pitch and electroplated with PEDOT:PSS to reduce the electrochemical impedance (Figure 3b; Figure S11d,f, Supporting Information). To test this hypothesis and validate the FE simulation results regarding the electrical fidelity properties of an ultrathin aGel interfacial layer, we prepared three device configurations: one without aGel (µECoG), one with 10 µm aGel (10‐µm aGel‐µECoG), and one with 100 µm aGel (100‐µm aGel‐µECoG) (Figure S20b–d, Supporting Information). Electrical characterization showed that the PEDOT:PSS coating effectively reduced the impedance and enhanced the charge storage capabilities across all configurations (Figure 3c; Figure S20e‐i, Supporting Information). Although the addition of 10‐µm and 100‐µm aGel layers slightly increased the impedance (Figure S20e–g, Supporting Information) due to the lower conductivity of aGel compared to that of PBS (Figure S10, Supporting Information), the values remained below 100 kΩ, ensuring negligible distortion when coupled with high input impedance amplifiers.

For a fair comparison, perfusion holes were incorporated into the µECoG devices to displace the CSF and minimize the source‐electrode distance (Figure S20a–d, Supporting Information). The devices were sequentially placed on the cortical surface of anesthetized rats to record signals from the left primary somatosensory cortex (Figure 3d; Figure S21, Supporting Information). Somatosensory evoked potentials (SSEPs) were elicited by stimulating the right sciatic nerve with 100 ms biphasic pulses using a pair of 26‐gauge bipolar needles (Figure 3a; Movie S2, Supporting Information). The recordings revealed highly localized, large‐amplitude SSEPs with peak responses at 20–30 ms post‐stimulation (Figure 3e), which is consistent with previous studies.^[^ ^17^, ^56^ ^]^ Trial‐averaged SSEPs from a representative measurement showed that the amplitude increased proportionally to the stimulation intensity for all the device configurations (Figure 3f,g). An unpaired t‐test applied to the normalized SSEP potential for each stimulation intensity revealed a significant difference for the 100‐µm aGel‐µECoG, starting at a stimulation current of 100 µA, indicating the threshold for a discernible response (Figure 3g). Further analysis across all stimulation conditions showed that aGel thickness significantly influenced signal power (two‐way ANOVA: F(2,15) = 14.98, *p* = 2.6 × 10^−6^). Post hoc Tukey's tests indicated no significant difference between µECoG and 10‐µm aGel‐µECoG (*p* = 0.42). In contrast, the 100‐µm aGel‐µECoG exhibited a significantly reduced signal power (*p* < 0.001) (Figure 3g). This observation is consistent with computational modeling predictions that thick aGel layers can attenuate signal propagation.

To assess the impact of aGel thickness on spatial resolution, we analyzed the bandpass‐filtered spatial maps of the SNR (Figure 3h). These maps revealed distinct activity patterns, particularly in the gamma (γ, 30–70 Hz) and high‐gamma bands (high γ, 70–190 Hz), demonstrating the ability of high‐density aGel‐µECoG to record high‐frequency oscillations. Notably, high‐gamma band activation is strongly associated with spiking activity and exhibits high spatial specificity.^[^ ^57^ ^]^ Figure 3h shows that both µECoG and 10‐µm aGel‐µECoG maintained sub‐millimeter spatial resolution at the gamma and high gamma bands, whereas 100‐µm aGel‐µECoG showed a broader spatial spread. We further quantified the spatial resolution using magnitude‐squared coherence across increasing inter‐electrode distances at different frequency bands using data recorded in the resting state (Figure 3h). The data were fitted to an exponential decay model, y = α·exp(‐x/λ) + c,^[^ ^58^ ^]^ where λ represents an approximation of the spatial resolution (Figure S22, Supporting Information). An unpaired t‐test applied to the spatial resolution for each frequency band revealed a significant difference for the 100‐µm aGe‐µECoG, indicating signal dispersion across all examined frequency bands (Figure 3i). A two‐way ANOVA confirmed that the aGel thickness significantly affected the spatial resolution (F(2,15) = 42.77, *p* < 0.001). While the 10‐µm aGel‐µECoG maintained a resolution comparable to that of µECoG (*p* = 0.41), the 100‐µm aGel‐µECoG showed a significantly degraded spatial resolution (*p* < 0.001).

These experimental results align well with our previously proposed design principles, providing robust validation of the simulation methodology. They also demonstrated that 10‐µm aGel‐µECoG arrays could achieve high‐resolution functional mapping comparable to high‐density µECoG, validating their potential for high‐fidelity neural recordings.

### 2.4 Histological Analysis Reveals Superior In Vivo Biocompatibility of aGel Coating

**Figure 4:**
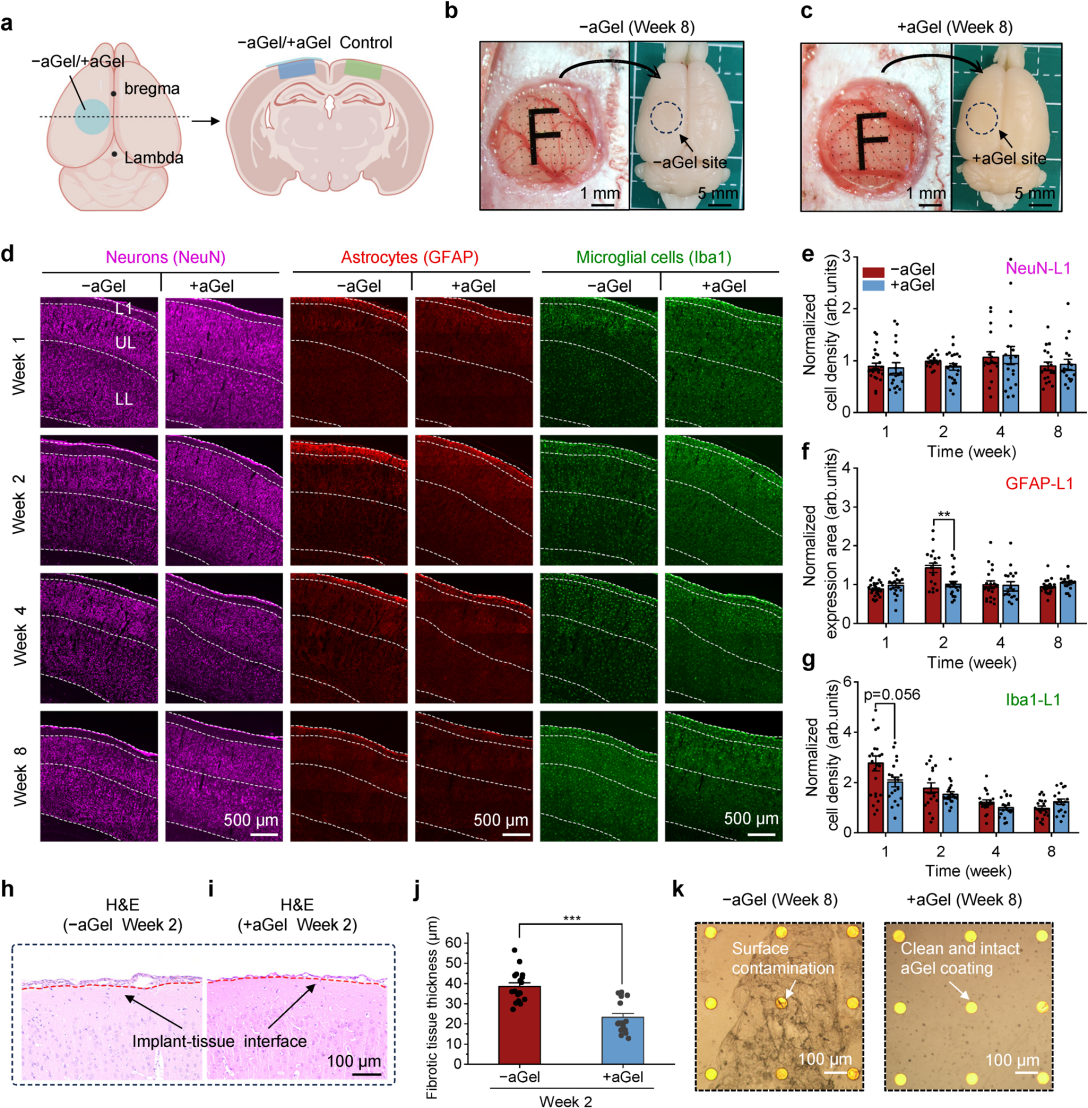
Histological evaluation of the in vivo biocompatibility of −aGel and +aGel. a) Illustration of the implantation of −aGel or +aGel and brain sections. The blue and green regions indicate the sections designated for quantitative analysis. Image was created with BioRender. b,c) Photographs of rat brains collected 8 weeks after implantation of the −aGel (b) and +aGel (c) devices. Images captured during implantation (left) and after perfusion (right), with a black circle indicating the implantation site. d) Representative immunofluorescence images at various time points. e–g) Quantitative statistical analysis of NeuN (e), GFAP (f), and Iba1 (g) expression in L1 at various time points. Data from the implant side were normalized to the contralateral control side for both the −aGel and +aGel groups (n = 24, 19 from five rats for week 1; n = 16, 21 from three rats for week 2; n = 18 from three rats for week 4; n = 18 from three rats for week 8). *p* = 0.0065 (GFAP L1, week 2). h,i) H&E imaging of brain tissues for −aGel (h) and +aGel (i) 2 weeks post‐implantation. The red dashed lines represent the implant‐tissue interface. j) Quantitative analysis of fibrotic tissue thickness in brain tissue two weeks post‐implantation with +aGel or −aGel (n = 18 from three independent rats). k) Representative photos of −aGel (left) and +aGel (right) films retrieved 8 weeks after in vivo implantation. Data are presented as mean ± SEM.

Next, we evaluated the in vivo biocompatibility of the aGel‐coated devices in rat models using chronic subdural implantation (**Figure** **4**a). To minimize adverse tissue reactions associated with connector depression^[^ ^19^ ^]^ and implant shape,^[^ ^59^ ^]^ we fabricated 4‐mm disc‐shaped devices in accordance with the ISO 10993–6 standards^[^ ^50^ ^]^ for the biological evaluation of medical devices (Figure S11b, Supporting Information). We compared two device configurations, one without aGel coating and one with a 10‐µm‐thick aGel coating (referred to as −aGel and +aGel, respectively) to distinguish them from the 64‐channel long‐term implanted devices. The polyimide‐encapsulated “F” pattern facilitated the orientation identification (Figure 4b,c (left)). Brain samples were collected at 1, 2, 4, and 8 weeks post‐implantation. Bright‐−field imaging revealed no visible cortical damage in either group (Figure 4b,c (right)).

To assess neuroinflammatory responses in the brain tissue underneath the implants, we used immunofluorescence markers for neurons (NeuN), astrocytes (GFAP), and microglia (Iba1) (Figure 4d). The expression levels were normalized to those of the contralateral controls^[^ ^20^ ^]^ to minimize individual variability (Figure S23, Supporting Information). To account for regional differences in cellular responses, the cortex was divided into three regions: layer I (L1), upper layers (UL, layers II–IV), and lower layers (LL, layers V–VI), based on neuronal density and morphology.^[^ ^20^, ^60^ ^]^ NeuN staining revealed no significant difference in neuronal density between the −aGel and +aGel groups at any stage of the study (Figure 4e; Figure S24, Supporting Information). Quantitative analysis revealed that GFAP expression, which is indicative of astrocytic activation, was significantly higher in all cortical regions of the −aGel group at two weeks post‐implantation (Figure 4f; Figure S24, Supporting Information). Similarly, Iba1 expression, a marker of microglial activation, was significantly elevated in the −aGel group compared to the +aGel group at one week in all cortical regions and at two weeks in the LL layer (Figure 4g; Figure S24, Supporting Information). After four weeks, no significant differences in marker expression were observed between the two groups. These results demonstrate the excellent biocompatibility of aGel‐µECoG devices, characterized by weaker in vivo immune responses than those of bare µECoG devices.

To evaluate the degree of fibrotic tissue growth, we examined the collected tissue samples using hematoxylin and eosin (H&E) staining (Figure 4h,i). The −aGel implants showed substantial fibrous capsule formation by week 2 (Figure 4j). Notably, loose connective tissues formed at the brain surface in the −aGel group as early as two weeks, consistent with a previous study.^[^ ^61^ ^]^ We also examined the fibrous tissue encapsulation of the explanted devices using second‐harmonic generation (SHG) imaging (Figure S25a,c, Supporting Information). A strong SHG signal and foreign material visible under an optical microscope were observed on −aGel discs, indicating significant collagen accumulation and protein adsorption.^[^ ^31^ ^]^ In contrast, no such signals were detected on the +aGel discs, indicating superior resistance to fibrotic capsule formation (Figure S25b,d, Supporting Information). Microscopic observations further confirmed that the aGel coating on explanted +aGel devices maintained its structural integrity across all time points. A representative image from week 8 is shown in Figure 4k (right panel). Additionally, a significant amount of adsorptive contamination was observed in the −aGel devices, whereas the +aGel devices remained clean (Figure 4k). These results collectively demonstrate the in vivo capabilities of aGel‐µECoG in mitigating neuroinflammation and reducing fibrous tissue formation in the brain.

### 2.5 Long‐Term Impedance Stability of aGel‐µECoG for Subdural Implantation

Electrochemical impedance spectroscopy (EIS) is a valuable tool for monitoring electrode‐tissue interface dynamics, with established correlations between in vivo impedance spectra and histological changes.^[^ ^10^, ^62^, ^63^ ^]^ Previous studies on long‐term surface electrode implantation have reported an initial rapid impedance increase followed by gradual stabilization over months to years, reflecting the progression of tissue responses from acute to chronic phases.^[^ ^10^, ^63^, ^64^, ^65^ ^]^

**Figure 5:**
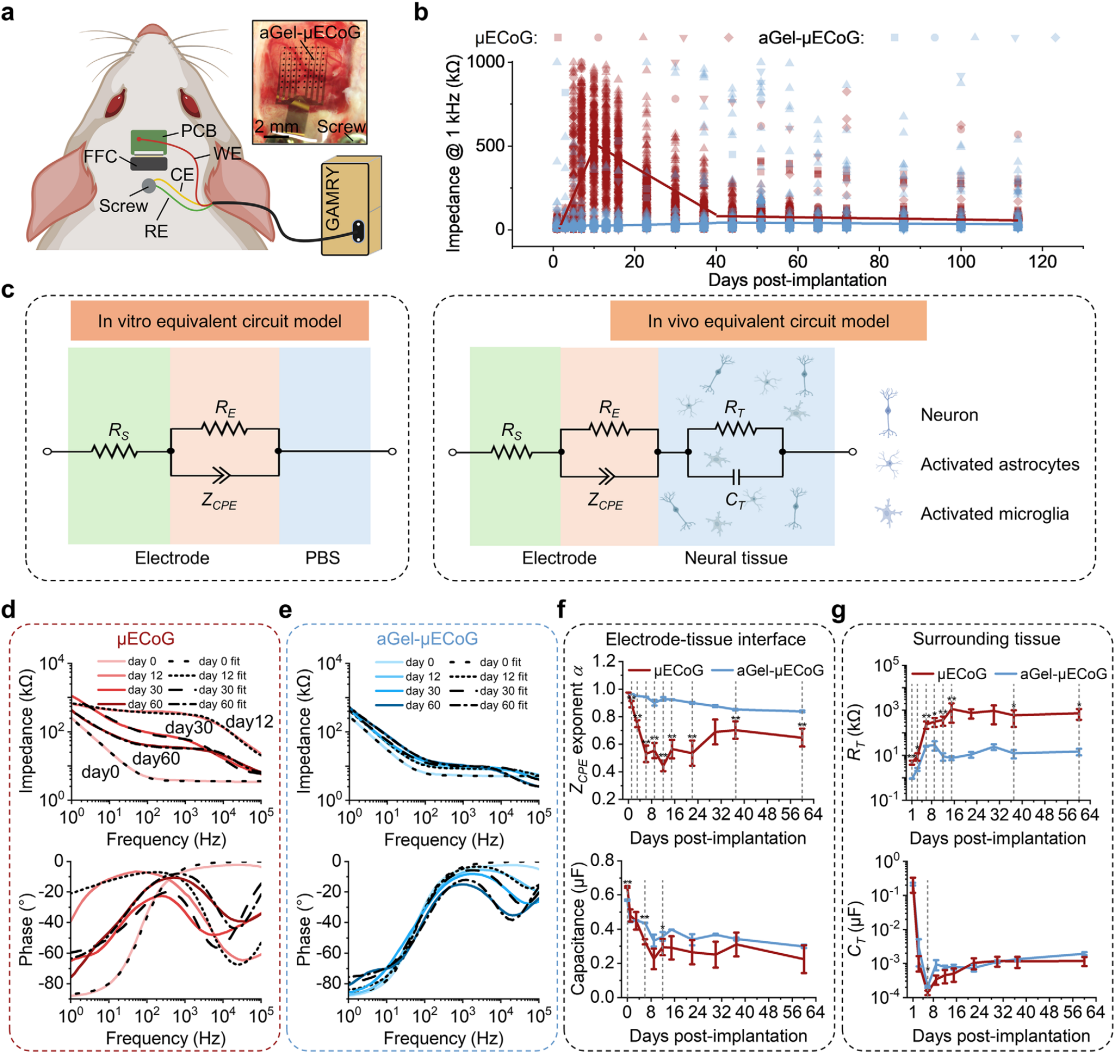
Long‐term evaluation of electrochemical impedance. a) Cartoon diagram illustrating in vivo EIS measurements using a two‐electrode system and a photograph of aGel‐µECoG placed on the visual cortex during the surgery. Image was created with BioRender. b) Distinct 1 kHz impedance trends of µECoG and aGel‐µECoG over 16 weeks (n = 5 rats). µECoG electrode exhibited a sharp increase from an initial value of ≈20 kΩ on day 1 to ≈540 kΩ on day 11 (slope = 55.8 kΩ d^−1^, *p* < 0.001), followed by a sharp decrease to ≈74 kΩ on day 40 (slope = −12.9 kΩ d^−1^, *p* < 0.001), and then decreased slowly (slope = −0.4 kΩ d^−1^, *p* < 0.001); aGel‐µECoG electrode exhibited a slow increase from initial value of ≈20 kΩ on day 1 to ≈27 kΩ on day 11 (slope = 0.8 kΩ d^−1^, *p* = 0.0827), followed by a slow increase to ≈41 kΩ on day 40 (slope = 0.6 kΩ d^−1^, *p* = 0.0077), and then decreased slowly (slope = −0.2 kΩ d^−1^, *p* = 0.0361). c) Equivalent circuit models for in vitro and in vivo conditions. Image was created with BioRender. d,e) EIS and fitted curves for µECoG (d) and aGel‐µECoG (e) on 0, 12, 30, and 60 days post‐implantation. f) Changes in the *Z*_CPE_ exponent term *α* and capacitance at the electrode‐tissue interface for µECoG and aGel‐µECoG over a 60‐day implantation period. n = 6 from two rats on each testing day. g) Changes in the resistive and capacitive components (*R*_T_ and *C*_T_) of the surrounding tissue for µECoG and aGel‐µECoG over the 60‐day implantation period. n = 6 from two rats on each testing day. Data are presented as mean ± SEM.

To evaluate the long‐term stability of the aGel‐µECoG, we employed 64‐channel µECoG arrays (Figure S11c, Supporting Information) and chronically implanted them in the visual cortex of rats (**Figure** **5**a). Two configurations were compared: µECoG without aGel and aGel‐µECoG with a 10‐µm interfacial aGel layer (Figure S26b,c, Supporting Information). Electrical characterizations (Figure S26d–g, Supporting Information) confirmed that the aGel layer minimally affected the electrical properties of the PEDOT:PSS‐electroplated µECoG. Accelerated aging tests in 60 °C PBS (1X, pH 7.4) for 100 days predicted an equivalent functional lifetime exceeding one year for both configurations,^[^ ^64^ ^]^ highlighting the durability of the PEDOT:PSS layer and aGel coating under simulated physiological conditions (Figure S27, Supporting Information). Electrochemical impedance was measured every three days for the first two weeks post‐implantation, and subsequently on a weekly or biweekly basis.

We first investigated the 1 kHz impedance as a fast and common metric for assessing the implant and tissue reactions. The results revealed distinct differences between the two configurations (Figure 5b; Table S3, Supporting Information). The µECoG control group exhibited significant impedance variability, characterized by three distinct phases with breakpoints on days 11 and 40, identified using a generalized additive model (GAM) (Figure S28b, Supporting Information). We observed an initial surge of impedance followed by a gradual decrease and recovery to baseline. The impedance fluctuations observed in this study reflect dynamic biological processes at the electrode‐tissue interface following implantation and are consistent with previous studies.^[^ ^10^, ^63^, ^64^ ^]^ The initial increase in impedance is driven by biofouling and protein adsorption, followed by a gradual decrease indicative of protein densification and tissue remodeling. By day 40, the impedance stabilized at 70.4 ± 1.2 kΩ (slope = −0.4, *p* < 0.001), suggesting that the reactive tissue response had reached equilibrium. Histological analysis supports these findings, showing a rapid initial increase in glial cell density at the implantation site, followed by stabilization marked by reduced glial cell activation and formation of tissue encapsulation. Although the impedance recovered to levels close to baseline, it remained slightly elevated, indicating persistent changes at the electrode‐tissue interface.

In contrast, aGel‐µECoG electrodes demonstrated remarkable stability throughout the implantation period, with impedance consistently remaining within a narrow range (32.3 ± 1.1 kΩ, Table S3, Supporting Information), indicating a more stable electrode‐tissue interface. Histological and SHG imaging revealed minimal fibrotic capsule formation in the arachnoid and explanted devices.

While single‐frequency impedance measurements provide a convenient metric for evaluating electrode functionality, wide‐frequency spectrum EIS combined with equivalent circuit modeling offers a more comprehensive understanding of tissue reactions at the electrode‐tissue interface.^[^ ^63^ ^]^ Combining EIS with circuit modeling (Figure 5c–e; Figure S28a,c–h, Supporting Information), we found that the µECoG group exhibited a significant decrease in the constant phase element (*Z*_CPE_) exponent term α and capacitance magnitude in the *Z*_CPE_ (Figure 5f), indicating a transition toward more resistive characteristics at the interface.^[^ ^62^, ^65^ ^]^ In contrast, the *Z*_CPE_ exponent term α and capacitance magnitude in the aGel‐µECoG group remained stable for two months. Additionally, the tissue resistance *R*_T_ increased rapidly in both groups during the acute phase (<2 weeks), but the µECoG stabilized at two orders of magnitude higher impedance than that of the aGel‐µECoG group (≈1000 kΩ vs ≈10 kΩ) (Figure 5g), due to the formation of less conductive encapsulation layer.^[^ ^66^ ^]^ Furthermore, the smaller tissue capacitance (*C*_T_) in the µECoG group after stabilization also suggested the formation of a thicker encapsulation layer,^[^ ^65^ ^]^ which is consistent with our histological analysis (Figure 4h–j). These results reinforce our findings that aGel‐µECoG preserves a stable electrode‐tissue interface in vivo.

### 2.6 Long‐Term Signal Stability of aGel‐µECoG

Electrophysiological signals with well‐defined patterns, such as visual evoked potentials,^[^ ^25^, ^30^, ^67^ ^]^ auditory evoked potentials,^[^ ^68^ ^]^ sensory evoked potentials,^[^ ^56^ ^]^ and epileptiform signals^[^ ^36^ ^]^ are commonly used to evaluate the chronic stability of implanted surface arrays. Among these, steady‐state visual evoked potentials (SSVEP) provide a non‐invasive and reliable measure of cortical activity owing to their consistent responses and minimal training requirements.^[^ ^68^ ^]^ SSVEPs elicit widespread activation of the visual cortex, ensuring uniform SNRs across the electrodes. Additionally, the SNR of the SSVEP is quantified in the frequency domain and is thus not susceptible to environmental noise.

**Figure 6:**
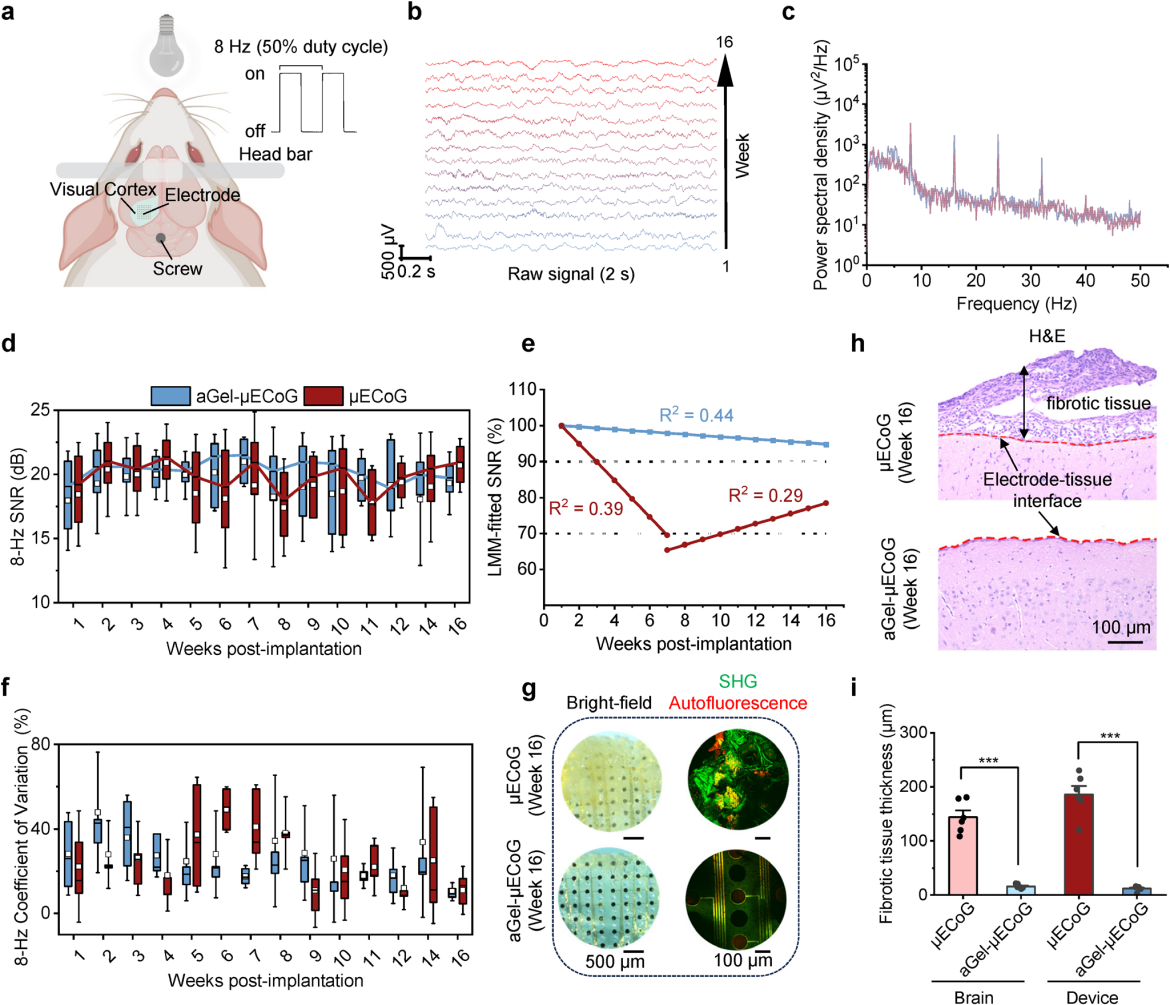
Long‐term signal stability of SSVEP recordings. a) SSVEP experimental setup. b) Time‐domain raw waveforms of a representative rat implanted with aGel‐µECoG for 16 weeks. c) Power spectral density of a representative rat implanted with aGel‐µECoG for 16 weeks, showing the frequency response at 8 Hz and its harmonics. d) SNR of 8 Hz harmonics for aGel‐µECoG and µECoG electrodes over 16 weeks. N > 200 independent electrodes from four or five rats for both types of arrays across all time points. e) LMM‐fitted SNR over weeks post‐implantation. f) Coefficient of variation of the 8‐Hz SNR shown in (d). g) Images of the µECoG electrodes (top) and aGel‐µECoG electrodes (bottom) taken 16 weeks after implantation. The photographs display the electrodes under bright‐field (left) and SHG images captured using a Nikon two‐photon microscope (right), with green representing the SHG signal and red representing spontaneous fluorescence, highlighting the presence of a thick fibrous encapsulation at the electrode‐tissue interface of the µECoG devices. h) H&E imaging showing fibrotic capsule formation in the brain tissue 16 weeks after the implantation of the µECoG electrodes (top) and aGel‐µECoG electrodes (bottom). The red dashed lines represent the electrode‐tissue interface. i) Quantitative statistical analysis of fibrotic tissue thickness on the arachnoid surface and beneath the device after 16 weeks of implantation with µECoG and aGel‐µECoG electrodes (n = 18 from three independent rats). Data are presented as mean ± SEM. In the box plots, the central line indicates the median, the box represents the interquartile range (IQR), the whiskers denote the SD, and the white dots represent mean values.

Similar to the long‐term evaluation of impedance, the 64‐channel aGel‐µECoG and µECoG were implanted in the left visual cortex of rats (**Figure** **6**a). The rats were head‐fixed and awake during recordings, with an 8‐Hz flashing light positioned 10 cm in front of their eyes (Figure S29 and Movie S3, Supporting Information). Light stimuli were delivered at a constant intensity with a 50% duty cycle to ensure consistent activations. The SSVEP signals were recorded weekly for the first 12 weeks and biweekly thereafter for up to 16 weeks. Representative results from an aGel‐µECoG electrode showed stable signal amplitude and power spectra over 16 weeks (Figure 6b,c), with periodic neural activity characterized by distinct peaks at 8 Hz and their corresponding harmonics. When the rat's eyes were covered, these frequency‐specific signals, particularly even harmonics (e.g., 16 and 32 Hz), disappeared, confirming that the visual cortex was the origin of the recorded activity (Figure S29e, Supporting Information).

To assess the signal stability over time, the SNR at 8 Hz was analyzed in five rats for each device type over a 16‐week period (Figure 6d). Signal stability analysis is challenging because of potential variabilities in the electrode‐tissue interface, individual subject differences, and attention and physiological status of the animals. To address these confounding factors, we employed a linear mixed‐effects model (LMM), incorporating electrode type (µECoG vs aGel‐µECoG), post‐implantation time, and their interaction as fixed effects, while accounting for unintended variations as random effects.

LMM modeling showed that both µECoG and aGel‐µECoG had similar baseline signal quality (*β*_1_ = 10.3, *p* = 0.594; Table S4, Supporting Information), corroborating our previous findings that a 10‐µm aGel coating does not change signal quality. The aGel‐µECoG electrodes maintained a consistent trend over time (*β*_2_ = −0.4, *p* = 0.029; Tables S4 and S5, Supporting Information), with a predicted SNR of 94.8% at 16 weeks post‐implantation. The µECoG arrays exhibited a significantly different SNR trend over time compared with the aGel‐µECoG arrays (*β*_3_ = −1.0, *p <* 0.001). We found that the µECoG exhibited a nonlinear SNR trend characterized by an initial decrease, followed by an increase. Using the minimum mean square error method, the data were segmented into two periods: before and after week 7. Our analysis revealed a significant SNR decrease before week 7 (*p* < 0.001, predicted SNR at week 7 = 69.5%) and a subsequent significant increase after week 7 (*p* < 0.001, predicted SNR at week 7 = 65.4%, at week 16 = 78.4%; Figure 6e; Tables S6 and S7, Supporting Information). Furthermore, intra‐electrode consistency analysis showed that aGel‐µECoG electrodes exhibited significantly lower coefficients of variation for an 8‐Hz SNR (*p* < 0.001, two‐way ANOVA, Figure 6f), indicating reduced SNR variability and a more homogeneous electrode‐tissue interface. The observed fluctuations and variabilities in signal quality for the µECoG group are likely driven by dynamic biological reactions at the electrode‐tissue interface. While the impedance begins to decrease after day 11, the decline in SNR persists until week 7, indicating a decoupling between these two metrics. These findings suggest that local changes in tissue resistivity, driven by inflammation and encapsulation, are the potential dominant factors influencing signal quality, which are consistent with previous studies.^[^ ^64^, ^69^, ^70^ ^]^

At 16 weeks post‐implantation, brain tissues were examined using H&E staining, and explanted devices were evaluated using bright‐field imaging, propidium iodide staining, and SHG imaging. The µECoG devices showed opaque, thick, and uneven fibrous tissue encapsulation under bright‐field imaging, with SHG imaging indicating strong collagen accumulation around the implants (Figure 6g, top panel). Propidium iodide staining of the extracted electrodes showed significantly enhanced cellular encapsulation on µECoG (Figure S30, Supporting Information), which correlated with the SHG results. H&E imaging revealed the formation of a thick fibrotic capsule beneath the µECoG on the brain slices. In contrast, aGel‐µECoG remained transparent, with no detectable fibrotic capsules on either the device or the brain surface (Figure 6g (bottom), Figure 6h (bottom); Figure S30, Supporting Information). Statistical analysis of H&E staining (Figure 6h) and propidium iodide staining (Figure S30, Supporting Information) revealed significantly thicker fibrotic capsules beneath the µECoG implants on both the brain surface (Figure 6i, left) and device surface (Figure 6i, right) than those of aGel‐µECoG. This nonhomogeneous fibrotic capsule may be the primary cause of signal fluctuations and increased variability across µECoG arrays. The findings indicate that while both electrode types demonstrate similar baseline performance, aGel‐µECoG electrodes exhibit enhanced longitudinal stability and spatial consistency during chronic implantation. This underscores their suitability for long‐term neural recording applications.

## 3 Discussion

Subdural ECoG arrays offer high spatial and temporal resolutions with superior signal quality, making them valuable tools for neural interfacing.^[^ ^9^, ^10^, ^23^ ^]^ However, their long‐term implantation often leads to neuroinflammation and fibrotic tissue growth at the electrode‐tissue interface, leading to progressive signal deterioration.^[^ ^10^, ^11^ ^]^ Recent advances have highlighted the potential of hydrogel‐based functional interfaces in bioelectronics due to their inherent biocompatibility, mechanical compliance, and bioadhesiveness.^[^ ^39^, ^40^, ^41^, ^42^, ^43^, ^44^, ^45^ ^]^ Here, we developed an aGel‐µECoG platform that integrates a flexible, high‐density µECoG array with an ultrathin PVA/PTPM hydrophilic/hydrophobic heteronetwork. The resulting hydrogel demonstrated excellent swelling resistance, mechanical compliance (Young's modulus of ≈109.4 ± 19.5 kPa), and robust bioadhesiveness (≈25.2 ± 3.8 kPa). Unlike chemically bonded adhesive strategies that pose potential risks of chemical contamination and tissue damage, we employed a dry cross‐linked hydrogen bonding mechanism. Our approach enabled a strong yet reversible bond to the delicate brain surface, ensuring mechanical stability while minimizing tissue damage.

We systematically investigated the hydrogel's impact on signal communication and optimized its design parameters for high electrical fidelity. High‐precision functional cortical mapping experiments confirmed that the optimized aGel‐µECoG preserved weak signal amplitude and maintained submillimeter spatial resolution. Long‐term implantation studies demonstrated its superior ability to mitigate neuroinflammation and suppress fibrotic tissue formation, resulting in a 20‐fold reduction in acute‐phase impedance increases compared to conventional µECoG arrays. Furthermore, aGel‐µECoG group maintained stable SSVEP recordings, with a signal‐to‐noise ratio of 94.8% over 16 weeks, significantly outperforming conventional µECoG arrays. While this study was conducted in rodent models, future work should explore the translational potential of this platform in large animal models and human trials, accounting for physiological differences in cortical and dura mater anatomy. Given the growing interest in the development of reliable and durable subdural neural interfaces, the aGel‐µECoG platform highlights promising strategies for enhancing accessible neuroprosthetic technologies and BCI systems.

## 4 Experimental Section

### Animals

All experiments were approved by the Institutional Animal Care and Use Committee (IACUC) of the Chinese Institute for Brain Research (CIBR), Beijing (license number: CIBR‐IACUC‐049). Male Sprague Dawley rats (8–15 weeks) were obtained from Beijing Vital River Laboratory Animal Technology Co., Ltd. The animals were housed in a standard facility at the CIBR under controlled environmental conditions, including a temperature range of 21–23 °C, humidity levels of 55–60%, and a 12‐hour light‐dark cycle. The animals were provided ad libitum access to water and food. Fresh porcine heart tissue was obtained from local butcher shops. To mitigate pain and reduce the risk of infection, meloxicam (2 mg kg^−1^) and ceftriaxone sodium (100 mg kg^−1^) were administered one hour before surgery and on postoperative days 0, 1, 2, 3, 5, and 7.

### Preparation of PVA/PTPM

The primary solution was prepared by dissolving 6 g of PVA‐1799 (98–99% hydrolyzed; Aladdin) in 50 mL of dimethyl sulfoxide (DMSO; Aladdin) under continuous stirring at 95 °C for 2 h. Subsequently, 0.85 mL (0.35 and 1.67 mL for PVA/PTPM ratios of 17:1 and 3.5:1, respectively) of 3‐(trimethoxysilyl) propyl methacrylate (TPM; Alfa) and 25 mg (10.54 mg and 50 mg for PVA/PTPM ratios of 17:1 and 3.5:1, respectively) of 2,2‐bimethoxy‐2‐phenylacetophenone (DMPA, 98%; Alfa) were added to the primary solution. The mixture was homogenized by stirring for 30 min in the dark to prevent premature photoreaction. Before assembly with µECoG, the resulting solution was defoamed by centrifugation (Cence, L500‐A) at 2000 rpm for 3 min.

### Fabrication of µECoG Arrays

The 1024/64‐channel µECoG arrays and 4‐mm discs were fabricated using standard microfabrication techniques on 4‐inch silicon wafers. Initially, a 3 µm layer of polyimide (PI2611, HD MicroSystems) was spin‐coated onto silicon wafers. The coated wafer was subjected to soft baking, followed by vacuum curing at 300 °C. Photolithographic patterning was performed using an AR‐N 4340 photoresist to define electrode structures. Subsequently, a 10 nm titanium layer and a 200 nm gold layer were deposited, and the excess material was removed through a lift‐off process. A second 3 µm polyimide layer was spin‐coated, soft‐baked, and vacuum‐cured. To define the electrode contours and contact pads, AZ 4620 photoresist was used as a hard mask for reactive‐ion etching (RIE). After the microfabrication steps were completed, the devices were optically inspected for defects. The devices were then released from the silicon wafer in deionized water, dried using N_2_ blowing, and transferred to glass substrates for packaging.

For the 64‐channel µECoG arrays, an Anisotropic Conductive Film (ACF) packaging method was employed. For the 1024‐channel electrodes, a gold ball‐bonding method was used.^[^ ^71^ ^]^ Both device types were integrated into custom‐designed flexible flat cables (FFC). During gold ball‐bonding, the electrode pad and FFC pad were precisely aligned and stacked, followed by bonding a gold ball within the electrode pad via to establish a secure connection between the electrode and FFC. For the 1024‐channel electrode arrays, Molex connectors (502430‐6421) were incorporated at the FFC edge, enabling reliable electrical interfacing with the corresponding Molex connectors (502426‐6421) on the Intan RHD 128‐channel headstage (C3316).

The electrodeposition of PEDOT:PSS was conducted in an aqueous solution containing EDOT (0.01 m) and PSS (2 wt.%) using NanoZ's fixed plating time mode.^[^ ^72^ ^]^

### aGel‐µECoG Assembly and Sterilization

The defoamed hydrogel solution was spread onto the µECoG arrays using a scrape‐coating technique to achieve precise thickness control. The coated aGel‐µECoG arrays underwent pulsed UV irradiation (365 nm wavelength, 0.8 W cm^−2^, 5 s On/3 s OFF) for 30 min, followed by solvent exchange in ultrapure deionized water (DI water, 18.2 MΩ·cm) for an additional 30 min. Next, aGel‐µECoG was immersed in sterile PBS (1X, pH 7.4, Macklin) for three days to ensure complete elimination of residual DMSO. PBS was renewed daily. The coating thickness was measured using a Stylus Profiler (DektakXT, Bruker). Prior to implantation, aGel‐µECoG arrays were dehydrated by baking at 60 °C for 15 min. The dehydrated arrays were then sealed in sterile packaging and subjected to UV sterilization for 30 min to ensure sterility.

### ATR‐FTIR Analysis of PTPM Formation

The formation of PTPM was characterized using attenuated total reflection Fourier transform infrared spectroscopy (ATR‐FTIR). Spectral measurements were conducted using an FT‐IR spectrometer (Bruker Equinox 55) equipped with ATR accessories. The spectra were recorded over a wavenumber range of 400–4000 cm^−1^.

### Measurement of Water Contact Angles

Water contact angles were measured with a contact angle meter (OCA25, Germany). Water contact angles were acquired on dry state aGel and PVA films. Water contact angles were also acquired on PI, fully swollen aGel, and PVA films to compare their hydrophilicity.

### Morphological Characterization via Cryogenic Scanning Electron Microscopy

The microscopic morphologies of the aGel and aGel‐µECoG specimens were analyzed using cryogenic scanning electron microscopy. The specimens were transferred into a cryo‐preparation chamber (LEICA EM ACE600) under vacuum conditions and subjected to sublimation at −100 °C for 20 min. Surface conductivity was achieved by platinum coating in an Ar atmosphere (15 mA, 200 s). The specimens were subsequently transferred to a microscope cryostage (S‐4300, HITACHI, Ltd., Japan) maintained at −137 °C. Image acquisition was performed using operating parameters of 3 kV landing energy and 10 µA current.

### Tensile Measurements of aGel

The mechanical properties of aGel were tested using fully swollen specimens in PBS and characterized using a universal testing machine (Instron5944 with a 1 kN load cell). Tensile tests were performed on unnotched dumbbell‐type specimens (GB/T 528–2009, length: 25 mm, width: 4 mm, thickness: 1.5 mm) at a constant tensile speed of 10 mm min^−1^. The specimens were loaded with the maximum strain until rupture. The fracture energy, which characterizes the material toughness, was determined by integrating the complete stress–strain curve and multiplying it by the initial specimen length:

(1) $$G_{f}=\left(\int_{0}^{\varepsilon_{f}}\sigma \left(\varepsilon \right)d\varepsilon \right)\cdot L_{0}$$

Young's modulus was calculated from the linear region of the stress–strain curve within the initial 10% strain range and is given by:

(2) $$E=\frac{\sigma }{\varepsilon }$$

where *ε* is the engineering strain, *σ* is the engineering stress, *L*_0_ is the initial length, and *ε*_f_ is the failure strain of the specimen.

### Lap Shear Adhesion Tests for PI Substrates

The adhesive performance of aGel was evaluated through lap‐shear tests (ASTM F2255 standard) by examining both the PI substrate and the biological tissue interfaces. In the lap‐shear adhesion test for PI, aGel was applied to one end of the PI substrate, and an area of approximately 25 mm × 25 mm was covered with another PI substrate and pressed to set. The specimens were tested after soaking in PBS for three days at room temperature and characterized by a universal testing machine (Instron5944 with a 1 kN load cell). The samples were stretched at a constant tensile speed of 10 mm min^−1^ until failure.

### Lap Shear Adhesion Test for Biological Tissue Interfaces

For the lap adhesion test at the tissue interface, the aGel was applied to one end of a stiff backing (glass) measuring approximately 25 mm × 25 mm. The biological tissue (porcine heart) was firmly adhered to another piece of glass backing and kept moist by spraying with water. The aGel with backing and tissue interface was pressed together and set for 30 min before the test. Lap shear adhesion tests were performed using a mechanical testing machine (TST‐01M with a 50 N load cell, PubTester). The specimens were pulled apart at a constant speed of 5 mm min^−1^ until failure. The adhesion strength of both configurations was calculated by dividing the force at failure by the overlap area.

### 90° Peeling Test for Interfacial Toughness

The interfacial toughness was determined using a 90° peeling test performed in accordance with ASTM D3330 standards. The test was conducted on an aGel sheet (dry state, dimensions: 30 mm in length, 15 mm in width, and 0.2 mm in thickness) adhered to a porcine heart immobilized using a glass backing. During the peeling process, the measured force reached a steady state once the peeling progressed uniformly. The interfacial toughness was calculated by dividing the steady‐state peel force by the width of the sample.

### Volume and Mass Swelling Ratio Measurements

The volume and mass swelling ratios were determined using dry‐state aGel sheets. The initial dimensions, including length (*l*_1_), width (*l*_2_), height (*h*_0_), and mass (*m*_0_), were recorded in the dry state. The specimens were then immersed in PBS at room temperature until equilibrium swelling was reached. After equilibrium was reached, the swollen dimensions were measured: length (*l*_1s_), width (*l*_2s_), height (*h*_s_), and mass (*m*_s_).

The volume swelling ratio (*Q*_v_) and mass swelling ratio (*Q*_m_) were calculated using the following equations:

(3) $$Q_{V}=\frac{l_{1s}\times l_{2s}\times h_{s}-l_{1}\times l_{2}\times h_{0}}{l_{1}\times l_{2}\times h_{0}}\times 100\%$$

(4) $$Q_{M}=\frac{m_{s}-m_{0}}{m_{0}}\times 100\%$$

### AC Conductivity Measurements

AC conductivity measurements were conducted on PBS and aGel specimens equilibrated in PBS using a four‐probe tester (ST2242, Suzhou Lattice Electronics) and a Gamry Reference 620 potentiostat. PBS was tested in a standardized vessel with dimensions of 4 mm (thickness), 20 mm (width), and 50 mm (length). For the aGel specimens, the individual dimensions were measured and recorded prior to testing.

The conductivity (*σ*) was calculated from the measured impedance (*R*) using the following formula:

(5) $$\sigma =\frac{1}{\rho }=\frac{1}{R}\cdot \frac{L}{A}$$

where *σ* is the conductivity (S m^−1^), *ρ* is the resistivity (Ωm), *R* is the impedance (Ω), *L* is the testing distance (probe spacing, m), and *A* is the cross‐sectional area of the specimens (m^2^).

### Measurement of Transmittance

Light transmission across the 400–800 nm wavelength range was measured using a UV–Vis spectrophotometer (Lambda 950, PerkinElmer). Test specimens were prepared by applying aGel coatings of varying thicknesses onto glass substrates, whereas uncoated glass substrates were used as reference blanks to account for substrate effects. Note that the aGel coatings were in a dry state during the tests. For direct comparison, the average transmission over the 400–800 nm wavelength was defined and obtained from the following equation:

(6) $$T\%=\frac{\int_{400}^{800}xdx}{800-400}$$

### In Vitro Cytotoxicity Tests

The cytotoxicity of −aGel and +aGel was evaluated following the ISO 10993‐12 standards. Films (thickness < 500 µm) were immersed in the culture medium at a ratio of 6 cm^2^ mL^−1^ and incubated at 37 °C for 24 h to prepare the extracts. NIH‐3T3 cells were cultured in Dulbecco's modified Eagle's medium (DMEM) supplemented with 10% fetal bovine serum and 1% penicillin‐streptomycin. Cells were subcultured upon reaching 90% confluency, seeded at 5 × 10^3^ cells per well in 96‐well plates, and incubated for 24 h.

After the initial incubation, the cells were exposed to the extract medium for 24 h, while the control groups were maintained in normal medium. Cell viability was assessed using a live/dead assay with calcein‐AM and propidium iodide for live and dead cells, respectively. Fluorescent images and live/dead cell ratios were obtained using an Opera Phenix high‐content screening system. Cell viability was calculated as the ratio of the total number of live cells to the total number of cells.

### Non‐Specific Protein Adsorption Experiments

Non‐specific protein adsorption was assessed following established protocols.^[^ ^73^ ^]^ PI, aGel, and PVA samples were cut to standard size and equilibrated in PBS overnight. They were then incubated in a 2 mg mL^−1^ BSA solution at 37 °C for 90 minutes. After incubation, loosely adsorbed proteins were removed via two PBS washes, while tightly bound proteins were desorbed using ultrasonication in 1% SDS solution. The protein concentration in the desorption solution was quantified using a micro‐BCA protein assay kit (Thermo Scientific, 23235).

### Cell Adhesion and Growth Experiments

The PI substrate was prepared by applying PI to the cell culture glass slides using the spin‐coating method. The aGel was coated onto the PI substrate using the same spin‐coating technique to produce the aGel substrate. Prior to use, the samples were sterilized using ultraviolet irradiation. After sterilization, the substrates were washed three times with PBS (3 min per wash) to remove residual contaminants. NIH3T3 cells were seeded at a density of 3 × 10^4^ cells per substrate and cultured at 37 °C for 1, 3, and 5 days. Viable cell density was quantitatively assessed using calcein‐AM/propidium iodide staining.

### Propidium Iodide Staining

The electrodes were stained with propidium iodide solution (Coolaber, China, 1 µg mL^−1^) for 7–8 min, followed by three washes with PBS solution (3 min per wash). The stained samples were imaged using a Nikon two‐photon microscope operated in the single‐photon mode.

### COMSOL Finite Element Model of the Rat Brain

To evaluate the effects of hydrogel thickness and conductivity, finite element models (FEM) of a rat brain and aGel‐µECoG electrodes were developed using COMSOL Multiphysics (Version 6.2, COMSOL Inc.).^[^ ^55^ ^]^ The rat brain was approximated as a multilayered spherical structure, including the gray matter, cerebrospinal fluid (CSF), dura mater, skull, and scalp. The aGel‐µECoG system was modeled as a thin, flexible layer conforming to the cortical surface, comprising a 6 µm‐thick PI substrate, PEDOT:PSS electrodes (20 µm diameter, 200 µm spacing), and an adhesive aGel layer. The thickness and electrical conductivity of each layer were assigned based on previously reported values and are summarized in Table S2 (Supporting Information). A schematic of the rodent head model is shown in Figure 2h. To optimize the computational efficiency while maintaining accuracy, a quarter‐sphere model was employed, leveraging the symmetry of the rat brain. A free tetrahedral mesh was generated with a finer mesh size at the electrode‐hydrogel‐cortex interface to capture the fine‐scale interactions. Convergence was achieved with a relative tolerance of 10^−3^. The FE models were computed using the AC/DC module. The scalp was grounded throughout the simulation.

### Design Optimization Using Computational Simulations

Neuronal recording was simulated by placing radially oriented dipoles at a depth of 0.3 mm in the gray matter, with a 100 µm separation between poles and currents of +1 µA and −1 µA applied to the upper and lower poles, respectively (Figure S16a,b, Supporting Information). Using this model, the study systematically swept two key parameters of the aGel layer—its thickness (*t*) from 0 to 100 µm and conductivity (*σ*) from 0.01 to 10 S m^−1^—and generated isopotential maps to analyze how these parameters influence the signal magnitude (Figure 2i; Figures S16 and S17, Supporting Information). This analysis revealed a clear trade‐off between the thickness and conductivity of the aGel layer: thinner layers or lower conductivities generally preserved stronger signals, whereas thicker or more conductive layers led to greater signal attenuation. For example, an aGel coating with a thickness of *t* < 30 µm and conductivity of σ ≈2 S m^−1^ can maintain a maximum signal amplitude exceeding 50% of the potential observed under aGel‐free conditions (Figure S16i, Supporting Information). Spatial resolution was characterized by potential decay profiles, with the critical resolution distance (*d*_c_) defined as the point where the normalized potential dropped to 0.1 of its initial value (Figures S16j and S17g, Supporting Information). This analysis revealed two key design principles: 1) for fixed σ, *d*_c_ decreases monotonically with decreasing *t*, and 2) at constant t, lower σ values yield smaller *d*_c_. These findings provide clear guidelines for designing aGel layers to balance the signal strength and spatial resolution in neural recording applications.

For electrical stimulation, the effects of aGel parameters on the voltage distribution within the brain were investigated by applying a constant 1 V voltage between adjacent electrodes (Figures S18 and S19, Supporting Information). The voltage distribution was quantified to determine how effectively the applied energy was transferred through the aGel layer into the surrounding neural tissue. To optimize the aGel design, the study introduced an energy transfer ratio (Δ*V*1/Δ*V*2), defined as the ratio of the potential obtained in the brain tissue below the electrodes to the potential applied to the electrodes. An energy transfer ratio of 0.5 was chosen in the analysis as the threshold for efficient stimulation, balancing the energy delivery to the brain while minimizing the losses within the aGel. Through this analysis, it was determined that the optimal aGel parameters for efficient stimulation were a thickness of 1–10 µm and a conductivity range of 0.1–2 S m^−1^.

Integrating these findings, an ultrathin aGel layer (≈10 µm thickness, ≈2 S m^−1^ conductivity) was identified as the optimal design for balancing high‐fidelity recording and stimulation efficiency.

### In Vitro Electrical Characterizations

The single‐frequency impedance at 1 kHz was measured using an Intan RHD 1024‐channel recording controller and RHD 128‐channel headstage, with a platinum electrode as the reference. CV and EIS were performed using a Gamry Reference 620 potentiostat with a platinum counter electrode and an Ag/AgCl reference electrode. CV measurements were conducted at a scan rate of 100 mV s^−1^ within a voltage range of −0.5 to +0.5 V. The EIS data were acquired over a frequency range of 1 Hz to 100 kHz using an AC voltage amplitude of 25 mV.

### Aging Experiments

The devices were aged by immersion in PBS at 60 °C. CV, EIS, and impedance measurements were performed at specific time points (0, 2, 4, 6, 8, 10, 12, 14, 21, and 28 days) and subsequently every two weeks or one month.

### Animal Surgery for Functional Cortical Mapping

Sprague Dawley rats (350–500 g, 10–15 weeks) were used for acute functional cortical recordings. Anesthesia was induced using 3–5% isoflurane and maintained at 1.5–3%. The scalp and right thigh were shaved, and the skin was disinfected with iodine and alcohol. To minimize pain and bleeding, 1% lidocaine (15 mg kg^−1^, SC) and etamsylate (125 mg kg^−1^, IP) were administered. Brain swelling was prevented by intravenous injection of 20% mannitol (2 g kg^−1^, IV). Prior to signal recording, the rats were transitioned from isoflurane anesthesia to an intraperitoneal injection of 5% ketamine (100 mg kg^−1^) and 10% xylazine (10 mg kg^−1^). Supplemental anesthetic doses were administered every 30 min.

A midline scalp incision was made to expose the skull, and the left temporalis muscle was excised. A square craniotomy (AP: 2.5 to −6 mm; ML: +1 to +7 mm) was performed using a stereotaxic apparatus (RWD Life Science, China). The bone flap was carefully removed, and the dura mater was excised. The exposed brain surface was kept moist with a saline‐soaked gelatin sponge, and bleeding was controlled prior to electrode placement.

For sciatic nerve preparation, a small incision was made on the contralateral thigh, and the connective tissues around the nerve were dissected. An insulated plastic sheet was placed under the nerve to prevent direct muscle stimulation, and a bipolar electrode was attached (Movie S2, Supporting Information).

### Bipolar Electrical Stimulation and Neural Recordings for Functional Cortical Mapping

Bipolar electrical stimulation of the sciatic nerve was performed using two disposable needle electrodes (Haizhen, EN‐ST2000; length: 13 mm, diameter: 0.40 mm). The cathode was then placed close to the spinal cord. A signal generator (DG4202, RIGOL) delivered 1 Hz square‐wave triggers to the Intan RHS 128‐channel stimulation/recording controller (M4200, Intan Technologies) and RHS 16‐channel stim/record headstage (M4016) to generate biphasic current pulses. The stimulation parameters were biphasic, cathodic‐first, 1 ms phase duration, and 1 Hz frequency. The stimulation intensity were 50, 100, 200, 500, or 1000 µA.

Neural signals were recorded using an INTAN 1024‐channel recording controller and eight RHD 128‐channel headstages, at a sampling rate of 10 kHz. Three device configurations (µECoG, 10‐µm aGel µECoG, and 100‐µm aGel µECoG) were tested for each rat. For each configuration and stimulation intensity, 1‐minute recordings were obtained. In addition, 5‐minute resting‐state signals were recorded for each electrode configuration.

### Data Recording and Processing for Functional Cortical Mapping

Neural signals were preprocessed using a 4th‐order bidirectional Butterworth bandpass filter (4–1000 Hz) and downsampled to 2000 Hz. Signals were segmented into individual trials, each spanning from a 0.5 s pre‐stimulus to a 0.5 s post‐stimulus. A trial was classified as “good” if the maximum amplitude within the post‐trigger window (0–0.5 s) exceeded 1.5 times the mean amplitude of the pre‐trigger baseline window (−0.5 to 0 s) (0.5 s duration, threshold multiplier = 1.5). Channels with impedance >10 MΩ or a good trial rate (ratio of good trials to total trials) of <10% were excluded from the study.

Baseline correction was applied by subtracting the pre‐stimulus means, and trial‐averaged responses were visualized for SSEP characterization. Good‐trial‐averaged and Hilbert‐transformed signals were used to compute the SNR, which was quantified as the root mean square (RMS) of the signals within the post‐stimulus window (0–100 ms) and normalized to the RMS of the pre‐stimulus baseline window (−100 to 0 ms):

(7) $$\mathrm{SNR}=RMS_{t_{0}}^{t_{100}}/RMS_{t_{-100}}^{t_{0}}$$

An adaptive re‐referencing pipeline was implemented to suppress common noise. Initial SNR maps were generated from good trials using a sliding thresholding window (0.2 s duration, threshold multiplier = 2), which guided the subsequent 2D Gaussian fitting. These maps were then fitted using a 2D Gaussian function as follows:

(8) $$G\left(x,y\right)=A\cdot e^{-\left(\frac{{\left(x-x_{0}\right)}^{2}}{2\sigma_{x}^{2}}+\frac{{\left(y-y_{0}\right)}^{2}}{2\sigma_{y}^{2}}\right)}$$

where *A* is the amplitude, (*x*_0_, *y*_0_) represents the centroid of the SNR maxima, and σ_*x*_, σ_*y*_ denote the spatial spread along the *x*‐ and *y*‐axes, respectively. The farthest 100 channels from the Gaussian centroid were selected for re‐referencing. The SNR maps were subsequently recalibrated using the re‐referenced signals.

Frequency‐resolved SNR mapping (Figure 3h) employed 4th‐order Butterworth bandpass filtering across canonical frequency bands (θ:4–8 Hz, α:8–12 Hz, β:12–30 Hz, low γ:30–70 Hz, and high γ:70–190 Hz).

For the stimulation intensity analysis (Figure 3f,g), spatially localized channels were selected from the high γ‐band (70–190 Hz) SNR map by retaining those with Gaussian‐fitted values ≥90% of the maximum. The mean amplitude of the raw signals (prior to the Hilbert transformation) within these channels was calculated to quantify the stimulation‐induced response. Post‐stimulus Hilbert‐transformed root mean square (RMS) amplitudes (10–200 ms) were normalized to those of aGel‐µECoG under 1 mA stimulation.

Resting‐state magnitude squared coherence analysis (Figure 3i; Figure S22, Supporting Information) used signal bandpass filtered between 1–1000 Hz, 50 Hz notch filtering, and adaptive common average re‐referencing. Continuous 5‐second epochs were segmented and analyzed for frequency‐specific coherence across various frequency bands using the multitaper method to estimate magnitude‐squared coherence. Coherence‐distance relationships were modeled with an exponential decay function: y = a⋅exp(−x/λ)+c to derive an approximation of the spatial resolution λ, where the spatial resolution parameter λ represents the characteristic decay length of coherence (smaller λ values indicate stronger spatial localization). The model parameters (a, λ, and c) were optimized via nonlinear least‐squares fitting.

### Immunohistochemical Analysis

To evaluate the biocompatibility of the +aGel and −aGel films, 4‐mm diameter discs for brain implantation were prepared according to the ISO 10993–6 standards. To distinguish between the anterior and posterior disc surfaces, an “F” mark was added owing to the transparency of the materials.

### Surgical Procedure for Histological Analysis

Sprague Dawley rats (250–350 g, 8–10 weeks) were anesthetized with isoflurane and positioned in the stereotaxic apparatus (RWD Life Science, China). Anesthesia and pain management were performed according to the previously described protocol. A circular craniotomy window with a diameter of 4.4 mm was created, centered at AP −3.2 mm and ML +2.9 mm, with reference to the bregma. The films were then purified, sterilized, and implanted subdurally. The removed bone flap was returned to its original position, and Kwik‐Sil (World Precision Instruments Inc., USA) was used to fill the gaps between the cranial bone. After curing the Kwik‐Sil, dental cement was applied to provide additional reinforcement. The skin was then sutured.

### Tissue Collection and Processing for Histological Analysis

At specified time points, the rats were anesthetized with urethane (1.5 g kg^−1^) and perfused transcardially with PBS until the liver became bloodless. Subsequently, 10% formalin solution was perfused until the body stopped trembling. The brains were removed, immersed in 10% formalin solution overnight, and dehydrated in 30% (w/v) sucrose solution the following day. Brains were coronally sectioned and mounted on a sample holder using an optimal cutting temperature (OCT) compound (Tissue‐Tek, USA). The sections were cut into 25 µm slices using a cryostat (Leica CM3050 S, Germany), mounted on glass slides, and stored at −80 °C.

### Immunostaining Protocol

Brain slices were washed three times with PBS (10 min each) and blocked for 1.5 h in 5% (w/v) bovine serum albumin (BSA) prepared in 1X PBST (0.5% Triton in 1X PBS). After blocking, the BSA solution was removed, and the slices were incubated overnight at 4 °C with the following primary antibodies: rabbit anti‐GFAP (1:500, Bioss #bs‐0199R‐1, USA), goat anti‐Iba1 (1:500, Abcam #ab289874, USA), and rat anti‐NeuN (1:500, Abcam #ab279297, USA). After primary antibody incubation, the slices were washed three times with PBS (10 min each) and incubated at room temperature for 1.5 h with the following secondary antibodies: Alexa Fluor 546 donkey anti‐rabbit (1:1000, Invitrogen #2411581, USA), Alexa Fluor 488 donkey anti‐goat (1:1000, AffiniPure #705545147, USA), and Alexa Fluor 647 donkey anti‐rat (1:1000, Jackson ImmunoResearch #712‐605‐153, USA), while avoiding exposure to light. The slices were then washed five times with PBS (10 min/wash). Finally, they were mounted using an anti‐fading mounting medium containing 4′,6‐diamidino‐2‐phenylindole (DAPI; Beyotime Biotechnology, China), stored at −20 °C, and protected from light exposure.

### Fluorescence Imaging and Analysis

Fluorescence imaging was performed using a VS200 Virtual Slide Microscope (Olympus, Japan) and analyzed using QuPath software (Queen's University Belfast, UK). The threshold and smoothing values were set in the “Thresholder” tool to identify the brain slice boundaries. Subsequently, the cortical regions were delineated using a custom‐written script, allowing manual adjustments of the curve range to enhance recognition accuracy. Owing to the high density of GFAP‐labeled astrocytic processes and the difficulty in identifying cell bodies, the proportion of GFAP‐positive areas in various regions was assessed. In contrast, NeuN‐labeled neurons had clear boundaries, allowing the quantification of the proportion of NeuN‐positive cells. For Iba1‐labeled microglia, the study distinctly identified cell bodies and defined co‐localization with DAPI as positive Iba1 expression, calculating the proportion of Iba1‐positive cells in various regions.

For GFAP analysis, regions of GFAP expression were classified as positive, whereas all other areas were deemed negative, and the highlighted edges of the brain slices were excluded from the area calculations. For NeuN and DAPI, “Cell Detection” was used to identify individual cells. Data from the implanted side were normalized to the control side to account for individual variability.

### SHG Imaging

SHG images were obtained using a two‐photon upright microscope (Nikon). The excitation wavelength for the SHG signal was 920 nm, and the detected light wavelength ranged from 457 to 463 nm. Additionally, a non‐specific fluorescence detection band was established to exclude interference from autofluorescence, with an excitation wavelength of 1040 nm and a received light wavelength of 564–629 nm.

### H&E Analysis

Brain tissue specimens were fixed in 10% neutral‐buffered formalin, followed by sequential gradient ethanol dehydration, xylene clearing, and paraffin embedding. Tissue sections (3–5 µm thick) were prepared using a rotary microtome. Prior to staining, the sections were dewaxed in xylene and rehydrated with a descending ethanol gradient. H&E staining was performed using standardized protocols, after which the sections were dehydrated using an ascending ethanol series and cleared in xylene. The stained sections were permanently mounted with a neutral resin mounting medium (Solarbio, China) and imaged under bright‐field illumination using a Leica DM3000 microscope (Leica Microsystems, Germany).

### Animal Surgeries for Chronic Study

Sprague Dawley rats (250–300 g, 8–9 weeks) were used for chronic recordings. Ten animals were divided into two groups: the control group (n = 5) was implanted with µECoG arrays, and the experimental group (n = 5) was implanted with aGel‐µECoG arrays. Animals were handled for two days prior to surgery for acclimatization. Anesthesia and pain management were performed according to the protocols described previously. After lidocaine application and tissue clearance, four self‐tapping screws were inserted into the skull to anchor dental cement (Sun Medical, Japan). A cranial window was drilled using a 0.5 mm drill bit (RWD Life Science, China) at coordinates relative to bregma: AP: −3.5 to −8 mm; ML: +0.5 to +5.5 mm. A screw electrode positioned at AP: −10 mm, ML: 0 mm was used as the ground and reference for electrical recording. The 64‐channel aGel‐µECoG and µECoG were placed on the visual cortex based on the rat brain atlas, and the bone flap was replaced over the cranial window. The skull was sealed using Kwik‐Sil (World Precision Instruments Inc., USA) and dental cement. A stud was fixed to the skull for head stabilization during awake recordings. A custom 3D‐printed cap was used to protect the connectors (Figure S29b, Supporting Information).

### Chronic In Vivo EIS Experiments

EIS was performed on 64‐channel aGel‐µECoG and µECoG electrodes implanted using the same surgical procedure as described earlier. Measurements were taken at 1, 3, 6, 9, 15, 22, 30, 37, and 60 days post‐implantation. Testing was conducted using a Gamry potentiostat with a cranial screw serving as both the reference and counter electrodes. The EIS measurement parameters were the same as those used for the in vitro EIS measurements.

### Equivalent Circuit Model

The in vitro and in vivo EIS analyses used different equivalent circuit models, as shown in Figure 5c. The in vitro EIS model comprised the access impedance of the circuit *R*_S_, electrode‐electrolyte/tissue interface resistance *R*_E_, and a Constant Phase Element *Z*_CPE_.^[^ ^62^ ^]^ The in vivo EIS equivalent circuit incorporated two additional elements, *C*_T_ and *R*_T_, which represent the capacitive and resistive components of the tissue, respectively. Note that the equivalent circuit components of the aGel layer contributed minimally and were not considered for simplicity. EIS was fitted using Gamry Echem Analyst with the Simplex Method algorithm, setting the Maximum Iterations to 300. The initial value ranges for *R*_S_ and *R*_E_ were determined based on the in vitro EIS data and the corresponding equivalent circuit model. These initial values were subsequently applied to fit the in vivo EIS data using the in vivo equivalent circuit model, enabling the extraction of the values for the remaining circuit elements. The impedance of the *Z*_CPE_ is defined as:

(9) $$Z_{CPE}=\frac{1}{Y_{0}{\left(j\omega \right)}^{\alpha }}$$

where *Y*_0_ is the *Z*_CPE_ constant, *j* is the imaginary unit, *ω* is the angular frequency, and *α* is the *Z*_CPE_ exponent, which ranges from 0 to 1. The *Z*_CPE_ functions as an ideal capacitor when *α* = 1, and as an ideal resistor when *α* = 0. The capacitance was calculated using the equation provided above.^[^ ^74^ ^]^

### 1 kHz Impedance Analysis

To characterize the change of electrode impedance over the days of implantation, GAM was employed to identify breakpoints and subsequently modeled each phase using linear regression. Only the electrode sites with impedance values between 1 kΩ and 1 MΩ were included in the analysis. The impedance changes over time were divided into three distinct phases, separated by two breakpoints. The first breakpoint was defined as the maximum impedance on the fitted curve, and the second breakpoint was defined as the intersection of the curve with the mean plus the IQR of the impedance values measured after 60 days, when stabilization occurred (Figure S28b, Supporting Information). Each phase was modeled using linear regression to quantify the relationship between the implantation duration and impedance. All analyses were performed using the *statsmodels* library in Python.

### SSVEP Data Recording

The day of surgery was designated as day 0. A signal generator (DG4202, RIGOL) was used to control a white‐light LED for the generation of visual stimulation. The signal generator was configured to produce an 8 Hz square wave (3 V high, 0 V low, 50% duty cycle). The LED emitted a luminous intensity of 32000 cd m^−2^ and was positioned 10 cm away from the rat eyes. The output from the generated signal was connected to a BNC T‐adapter to synchronize the signal between the LED and INTAN recording system.

To minimize movement artifacts, the rats were restrained using a custom‐designed jacket that stabilized their bodies and lifted them slightly. A stud fixed to the skull during surgery was used for head fixation, eliminating the interference caused by head movements (Figure S29c, Supporting Information). Two days before the recording, the animals were habituated to the setup by placing them in a jacket. Each recording session consisted of a 10‐second resting‐state recording followed by a 3‐minute SSVEP recording (Movie S3, Supporting Information). To confirm that the recorded signals originated from brain activity, an eye‐covered test was conducted (Figure S29c–e, Supporting Information). During this test, the rats’ eyes were covered with an eye mask, and the recording procedure was repeated as described above.

### SNR Analysis for SSVEP

Fast Fourier Transform processing was performed on the recorded SSVEP signals from 10 to 180 s. The SNR was computed using the following formula:^[^ ^32^ ^]^

(10) $$SNR=\frac{\left(n-1\right)\cdot P\left(f_{t}\right)}{\left(\sum_{f=f_{1}}^{f_{2}}P\left(f\right)\right)-P\left(f_{t}\right)}\;$$

where *P(f)* denotes the power calculated by FFT across frequencies *f*_1_ = 7 Hz to *f*_2_ = 9 Hz, *f_t_* is the target stimulation frequency equal to 8 Hz, and *n* represents the number of frequency bins between *f*_1_ and *f*_2_. To mitigate transient artifacts, epochs containing signal amplitudes exceeding 2 mV were considered as artifacts and were automatically removed.

### Mixed‐Effects Regression Modeling of SSVEP

To analyze the signal stability during long‐term implantation, LMM was employed to account for the complexity of in vivo data recording. Random effects were included to capture the variability across individual rats, electrode channels, and other unintended sources of variation. The LMM was designed to estimate the fixed effects of implantation time (*Time* in week), electrode type (*ElectrodeType*), and their interaction (*Time × ElectrodeType*) on SNR, while minimizing the influence of random effects. The model is formulated as follows:

(11) $$\begin{array}{c} Y=\beta_{0}+\beta_{1}\cdot ElectrodeType+\beta_{2}\cdot Time+\beta_{3}\cdot \left(ElectrodeType\times Time\right)+\mu +\nu +\varepsilon \end{array}$$

here, *Y* represents the SNR (on a linear scale); *β*_0_ represents the intercept, corresponding to the baseline SNR (i.e., *Time* = 0) for the aGel‐µECoG electrodes (i.e., the reference group); *β*_1_ represents the difference in baseline SNR between the µECoG and aGel‐µECoG electrodes; *β*_2_ represents the temporal trend in SNR over time for the aGel‐µECoG electrode; and *β*_3_ represents the interaction effect, capturing the differences in time‐dependent trends between the µECoG and aGel‐µECoG electrodes. The random term *µ* represents the random effect for each rat, accounting for subject variability, and *ν* represents the random effect for each electrode, capturing electrode variability. The residual term *ε* represents the unexplained error or noise that follows a normal distribution. Finally, the SNR retention rate was determined by calculating the ratio of the predicted values at each time point to those from the first week.

### Statistical Analysis

All statistical analyses were performed using Origin 2021. The Kolmogorov‐Smirnov test was used to assess the normality of data distribution. For cytotoxicity and cell adhesion experiments with a normal distribution, one‐way ANOVA was conducted; otherwise, the Kruskal‐Wallis test was used for significance analysis. For electrophysiological data, two‐way ANOVA with Tukey's multiple comparison test and two‐tailed unpaired *t*‐test were used. For in vivo histological analysis and EIS experiments that were normally distributed, a two‐tailed unpaired *t*‐test was conducted; if the data were not normally distributed, the MannWhitney test was used. The significance thresholds were set at ns *p* > 0.05, * *p* < 0.05, ** *p* < 0.01, and *** *p* < 0.001. Data are presented as mean ± SEM, unless otherwise specified.

## Conflict of Interest

The authors declare no conflict of interest.

## Author Contributions

L.C., H.Z., L.W., and L.X. contributed equally to this work. T.W. and D.Q. designed and supervised the experiments. L.C. and L.X. prepared and characterized the PVA/PTPM hydrogels. L.C., Y.Z. and H.‐L.Z. prepared and characterized µECoG and aGel‐µECoG. K.W. performed the COMSOL simulations. H.Z., L.W., Y.Z, J.G., and K.L. conducted behavioral and electrophysiological measurements. L.W., Y.S., and L.C. performed electrophysiological data analysis. W.F. and X.F. performed the histological studies. H.‐L.Z. performed the aging tests, the in vivo EIS measurements and equivalent circuit modeling. T.W. wrote and reviewed the manuscripts with inputs from all the co‐authors.

## Supporting information



Supporting Information

Supplemental Movie 1

Supplemental Movie 2

Supplemental Movie 3

## Data Availability

The data that support the findings of this study are available from the corresponding author upon reasonable request.
