# Supplementary material for: Long‐Term Stable Subdural Recordings Enabled by Fibrosis‐Resistant Hydrogel‐Integrated µECoG Arrays
Source: Adv Sci (Weinh). 2025 Oct 8;12(47):e15453. doi: 10.1002/advs.202515453 (PMC12713035; doi:10.1002/advs.202515453)
Supplement: Supplementary file 1 — Supporting Information [file ADVS-12-e15453-s002.docx]

Supporting Information

**Title: Long-Term Stable Subdural Recordings Enabled by Fibrosis-Resistant Hydrogel-Integrated μECoG Arrays**

*Lin Chen, Hao Zhong, Linghao Wang, Liju Xu, Wanying Fan, Yongpeng Zhao, Huiling Zhang, Yang Shen, Kai Wu, Xin Fu, Jingxiang Guo, Ke Li, Dong Qiu*, Ting Wu**

**This file includes:**

Figures S1 to S30

Tables S1 to S7

Descriptions for Movies S1 to S3

**Other Supporting Materials for this manuscript include the following:**

Movies S1 to S3


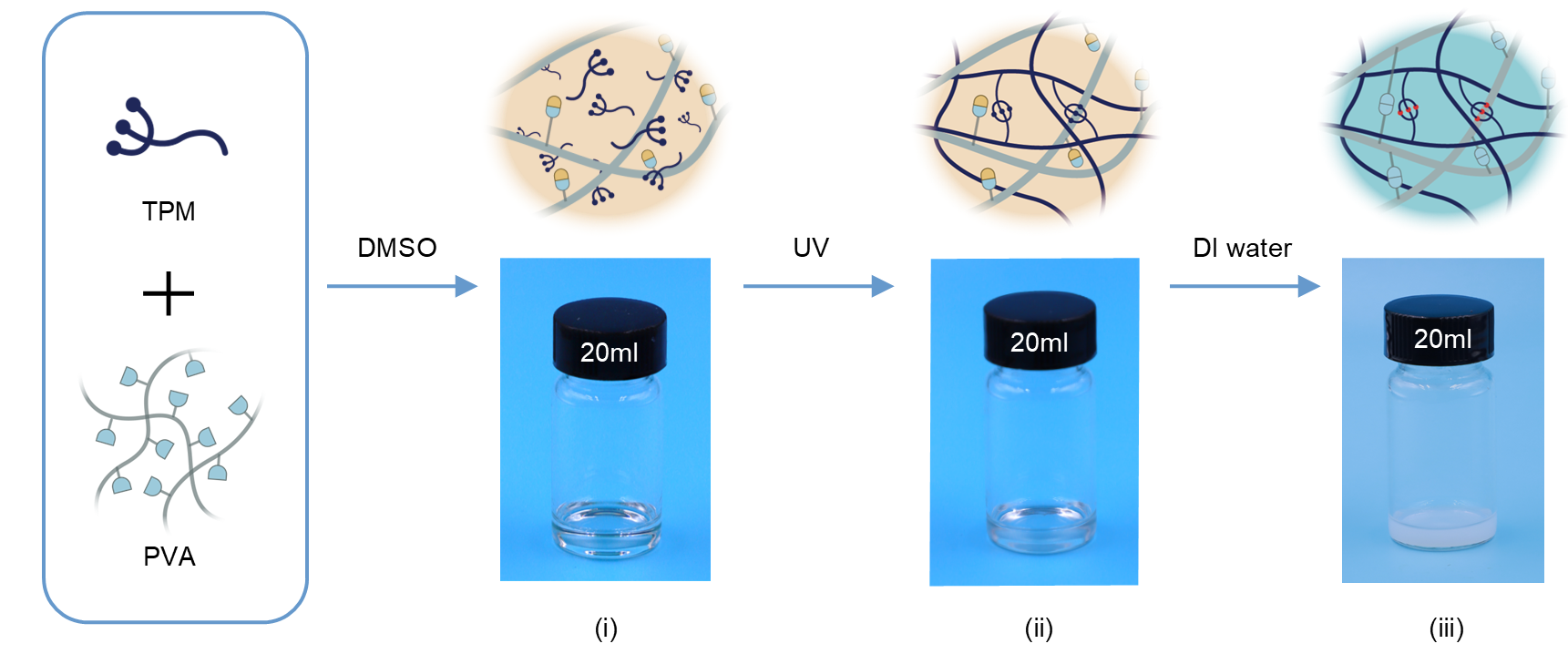


**Figure S1. Schematic illustrations and photographs of the aGel synthesis process.** (i) The initial solution system comprised DMSO as a solvent, with PVA and TPM thoroughly dissolved and homogeneously mixed. (ii) The TPM monomers underwent complete polymerization under UV irradiation in the presence of photoinitiator. (iii) The final phase involved solvent exchange, in which DMSO was displaced by DI water.


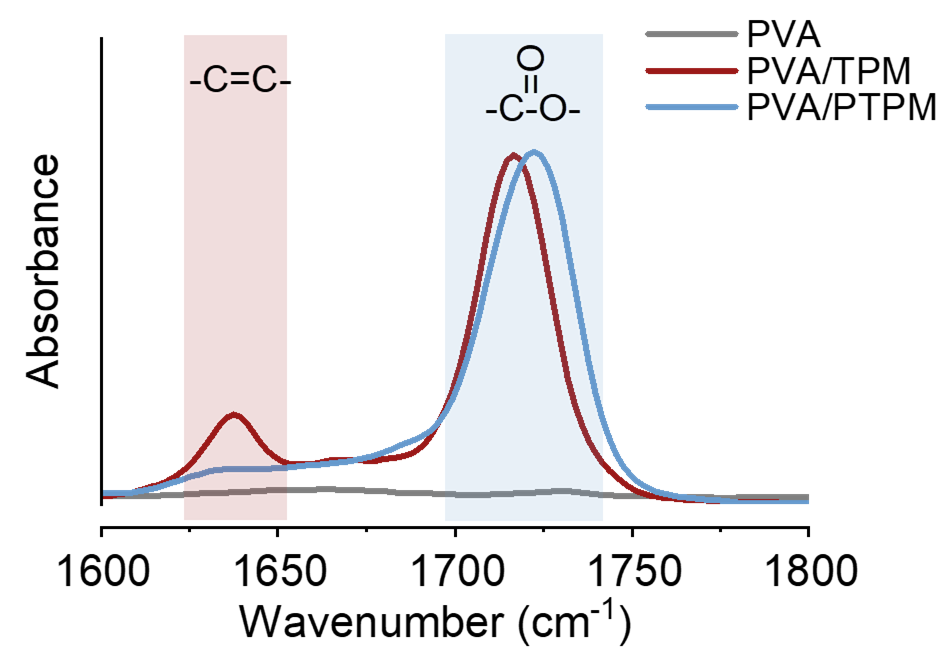


**Figure S2. FT-IR spectra of the PVA/PTPM, PVA/TPM, and pure PVA solutions.** The characteristic absorption peak corresponding to the C=C double bond (1635 cm⁻¹) was no longer detected following photopolymerization, confirming that the TPM monomers initiated by UV irradiation were successfully synthesized into PTPM. Additionally, the C=O-O ester group absorption shifted from 1715 cm^−1^ to 1725 cm^−1^, which was attributed to the disruption of the conjugated structure.

**
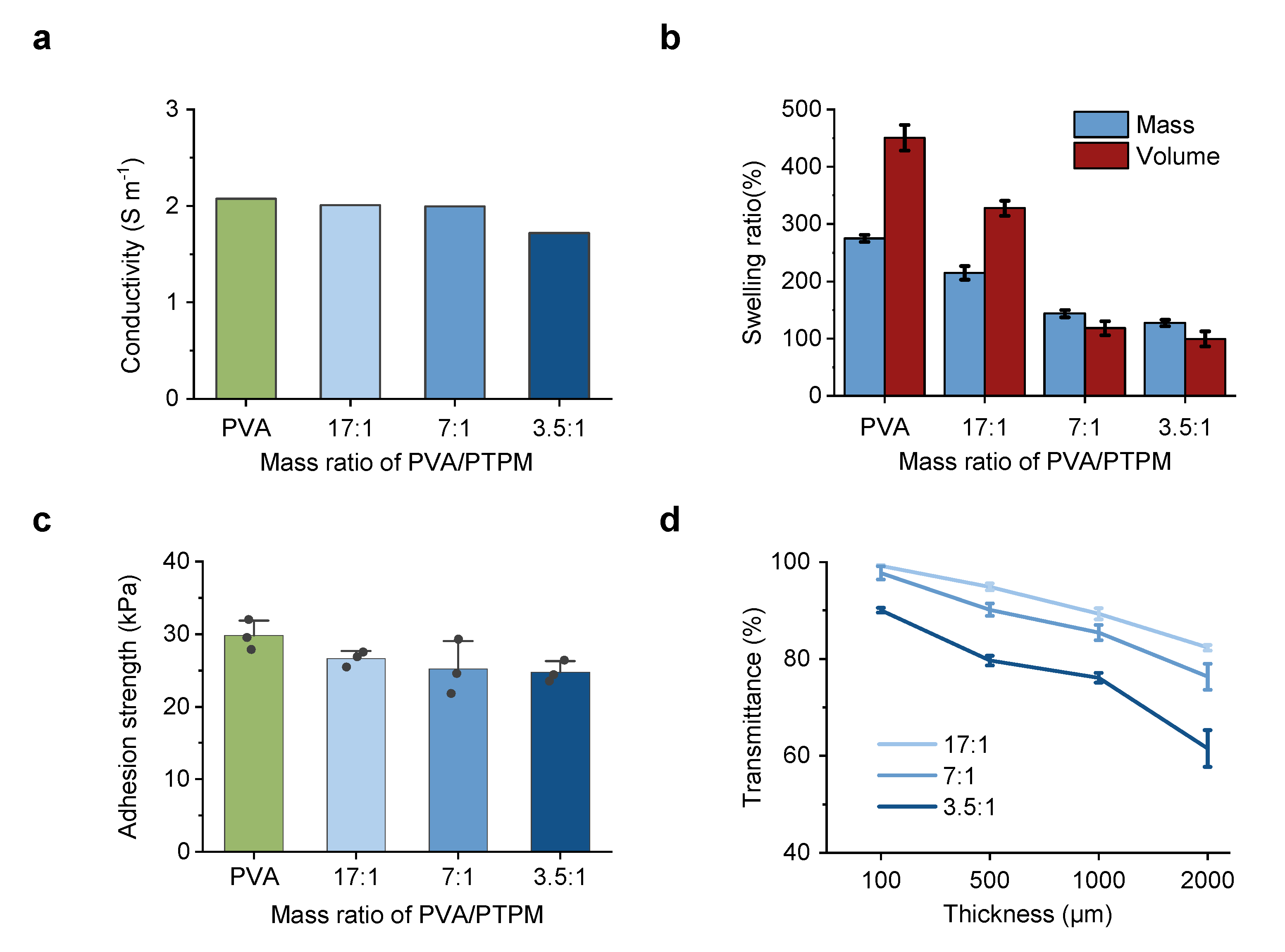
**

**Figure S3. Comparison of properties of PVA/PTPM hydrogels with different compositions.** **a)** Ionic conductivity. **b)** Swelling kinetics of hydrogels upon immersion in 1x PBS. **c)** Tissue adhesion strength measured by lap-shear tests. **d)** Average optical transmittance of hydrogel coatings on glass slides as a function of mass ratio and coating thickness. Data are presented as mean ± SD.


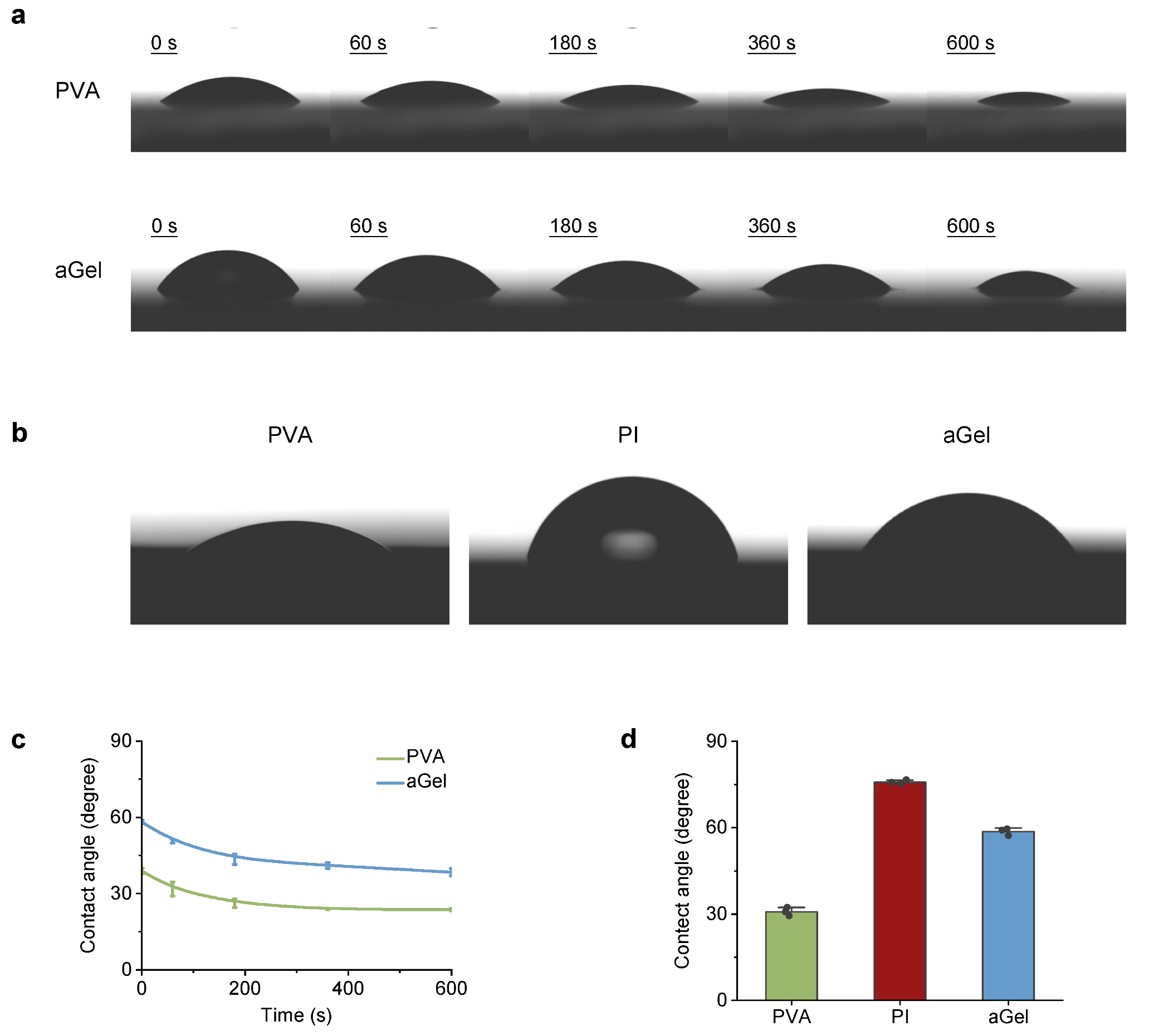


**Figure S4. Water contact angle of the PVA, aGel, and PI films. a)** Representative water contact angle images of dry state aGel and PVA films. **b)** Water contact angle images of PI, fully swollen aGel, and PVA films. **c)** Quantitative analysis of water contact angles over time for dry state aGel and PVA. **d)** Statistical comparison of water contact angles for PI, fully swollen aGel, and PVA films. n = 3 independent samples. Data are presented as mean ± SD.


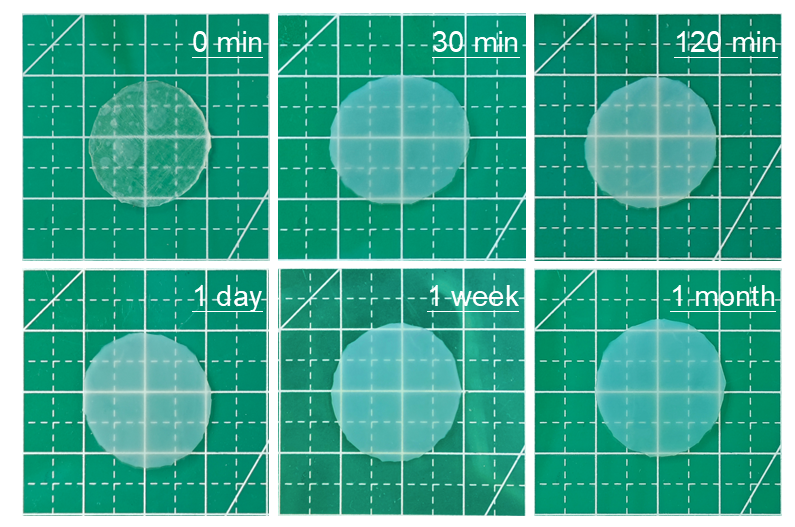


**Figure S5. Temporal characterization of aGel swelling behavior in PBS solution.** Sequential optical images showing the progressive swelling of aGel in PBS solution at various time intervals. The initially dry aGel sheet achieved an equilibrium swelling state within three hours in the PBS solution and maintained stable over 1 month.


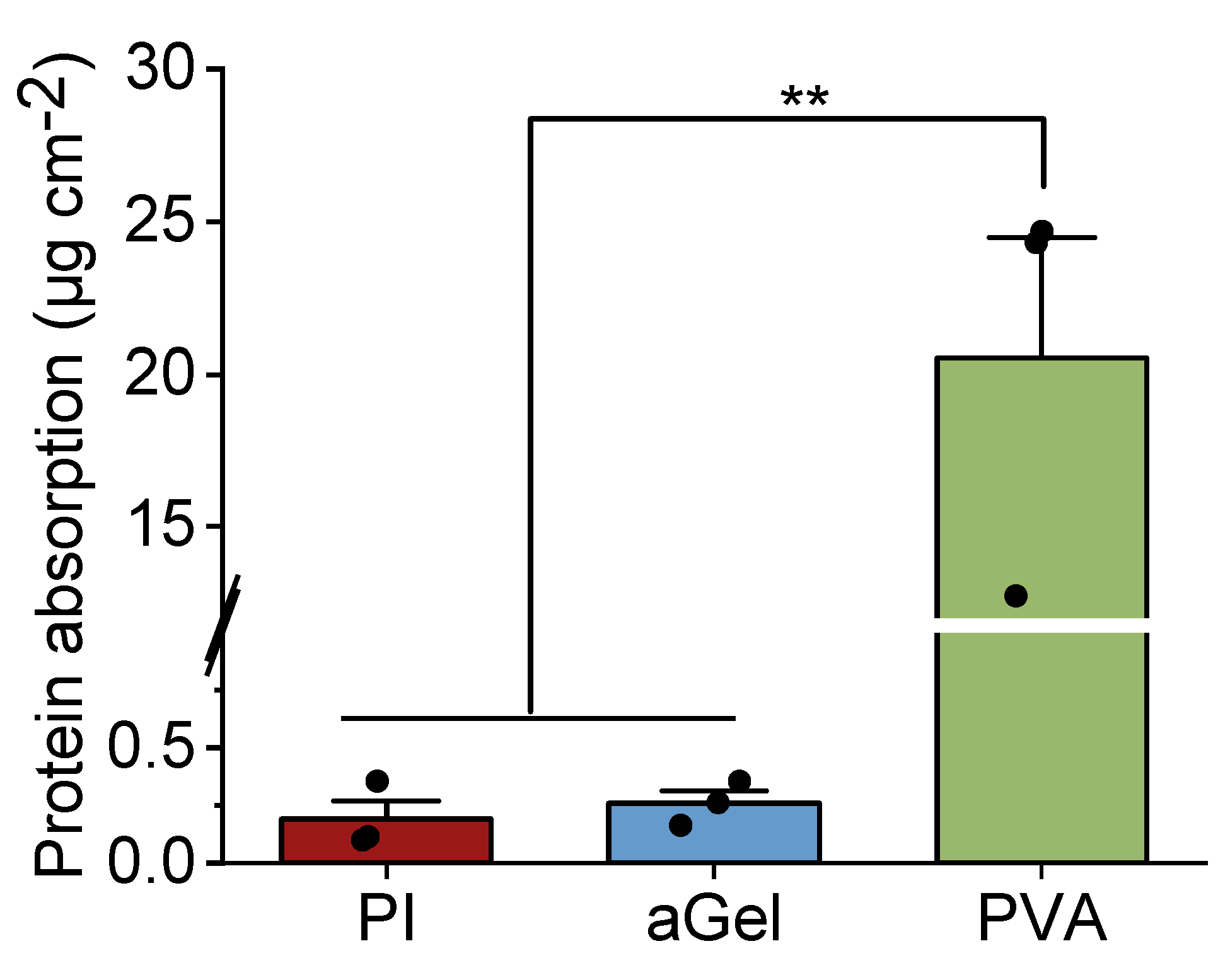


**Figure S6. Assessment of protein adsorption on PI, aGel and PVA.** Quantitative analysis of protein adsorption on the surfaces of PI, aGel, and PVA (n = 3). Data are presented as mean ± SEM. Data following a normal distribution were analyzed using one-way ANOVA; otherwise, the Kruskal-Wallis test was used for significance analysis. **p* < 0.05, ***p* < 0.01, ****p* < 0.001.


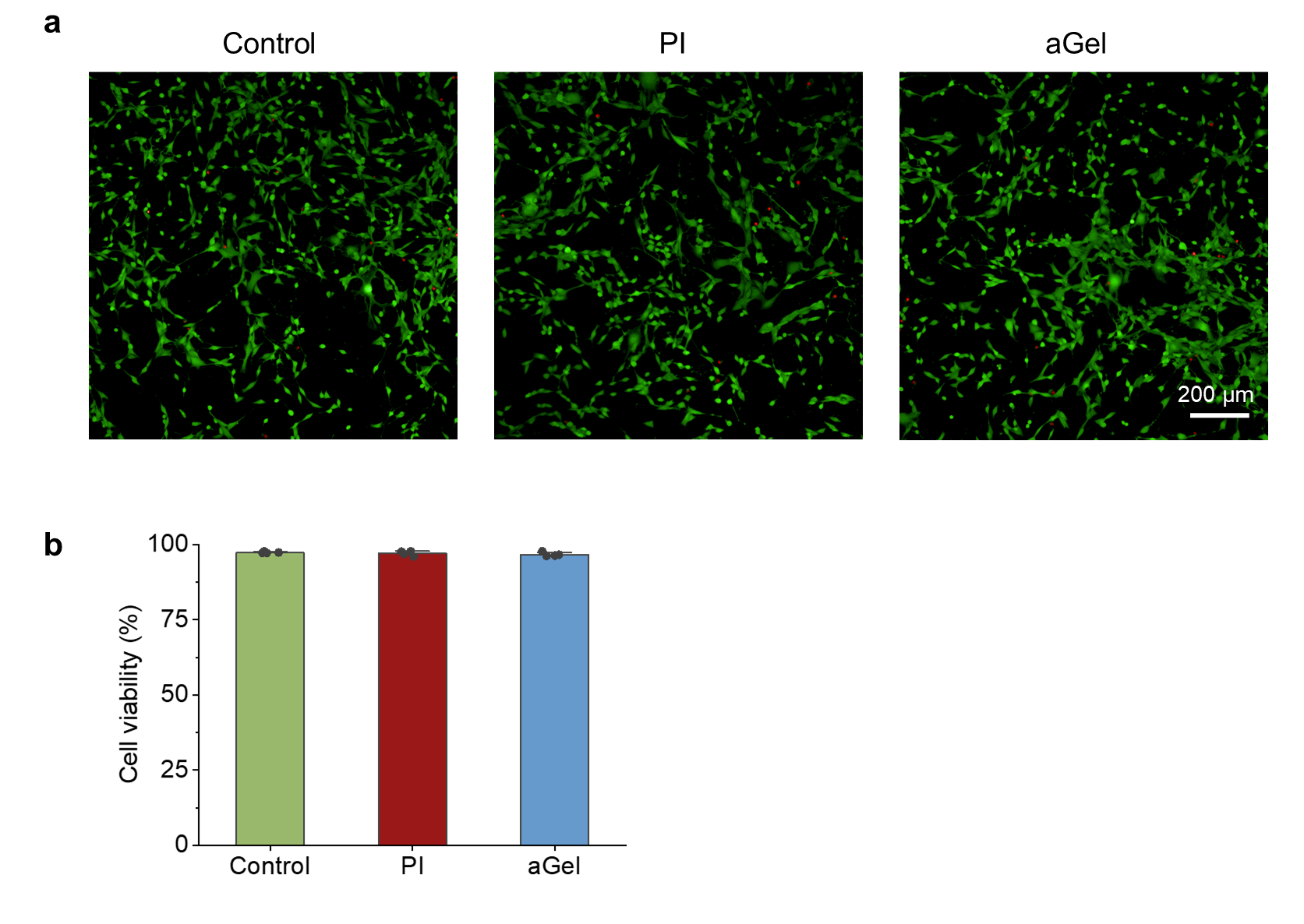


**Figure S7. Cell viability assessment of NIH3T3 cells after calcein-AM/propidium iodide staining. a)** Representative fluorescence images of cells following a 24-hour culture in different media. Control represents the culture medium, PI represents 100% PI extract, and aGel represents 100% aGel extract. Green dots indicate live cells and red dots indicate dead cells. **b)** Quantitative assessment of calcein-AM/propidium iodide staining results following a 24-hour incubation of cells with culture medium, 100% PI extract, or 100% aGel extract (n = 4). Data are presented as mean ± SD.


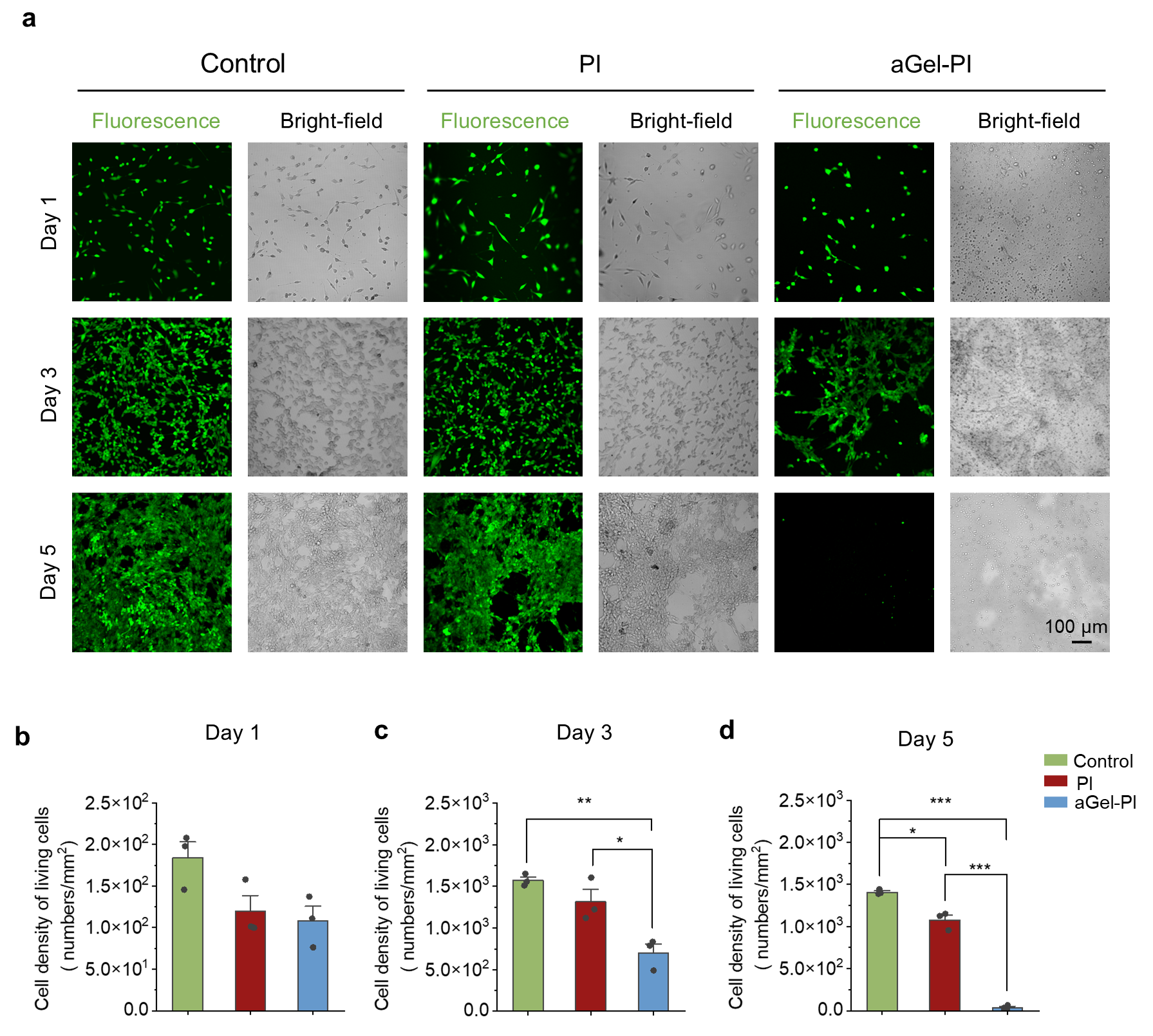


**Figure S8. Assessment of NIH3T3 cell adhesion and growth on different biomaterial substrates. a)** Representative fluorescent images of NIH3T3 cells cultured on control (glass), PI, and aGel-PI substrates for 1, 3, and 5 days, with live cells stained green using calcein-AM. **b-d)** Quantitative analysis of live cell density on control (glass), PI, and aGel-PI substrates after 1, 3, and 5 days of culture (n = 3). Data are presented as mean ± SEM. Data following a normal distribution were analyzed using one-way ANOVA; otherwise, the Kruskal-Wallis test was used for significance analysis. **p* < 0.05, ***p* < 0.01, ****p* < 0.001.


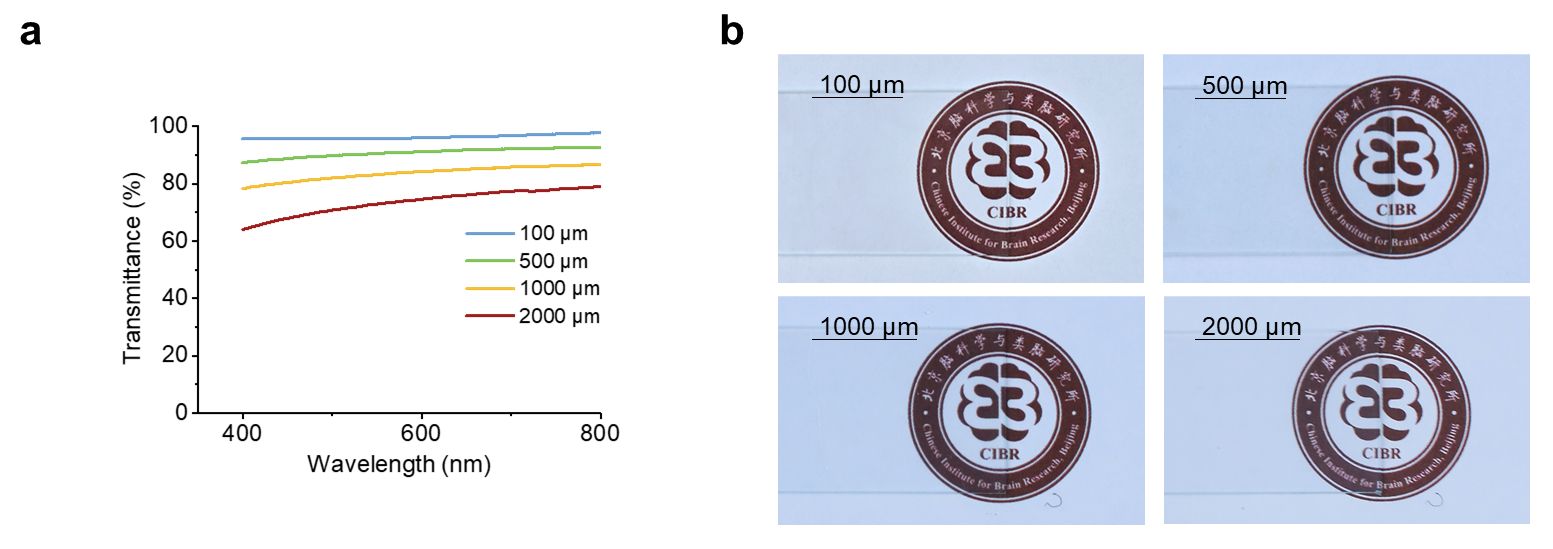


**Figure S9. Optical transmittance characterization of aGel coatings. a)** Spectral transmission analysis of aGel coatings with different thicknesses (100 μm, 500 μm, 1000 μm, and 2000 μm) applied to glass substrates. The optical transmittance of aGel coatings exhibited an inverse relationship with the coating thickness; however, within the practical application range (≤ 500 μm), the coating maintained a high visible light transmittance exceeding 85%. **b)** Optical images of aGel coatings with different thicknesses (100, 500, 1000, and 2000 μm) applied to glass substrates.


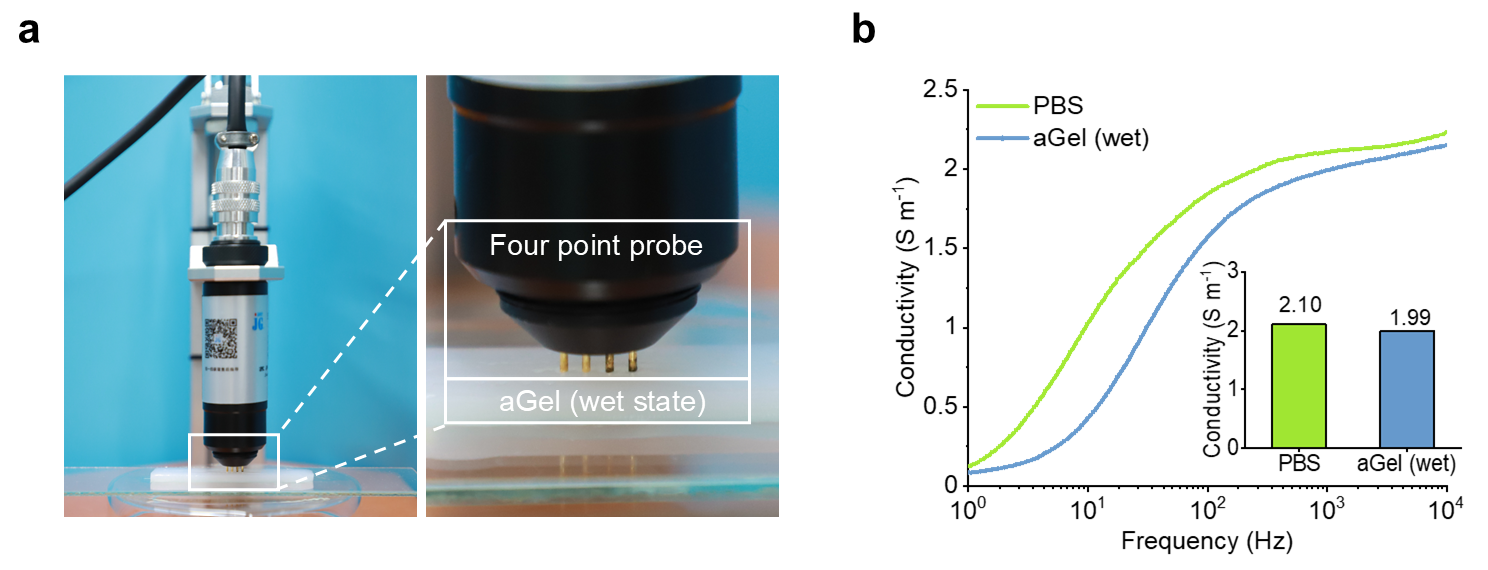


**Figure S10. Ionic conductivity measurements of aGel (wet state). a)** Experimental setup for conductivity measurements. **b)** Conductivity of aGel in the wet state and PBS (1x, pH 7.4) solution. The inset shows the conductivity at 1 kHz. The conductivity of the aGel (wet state) was 94% of that of the PBS solution.


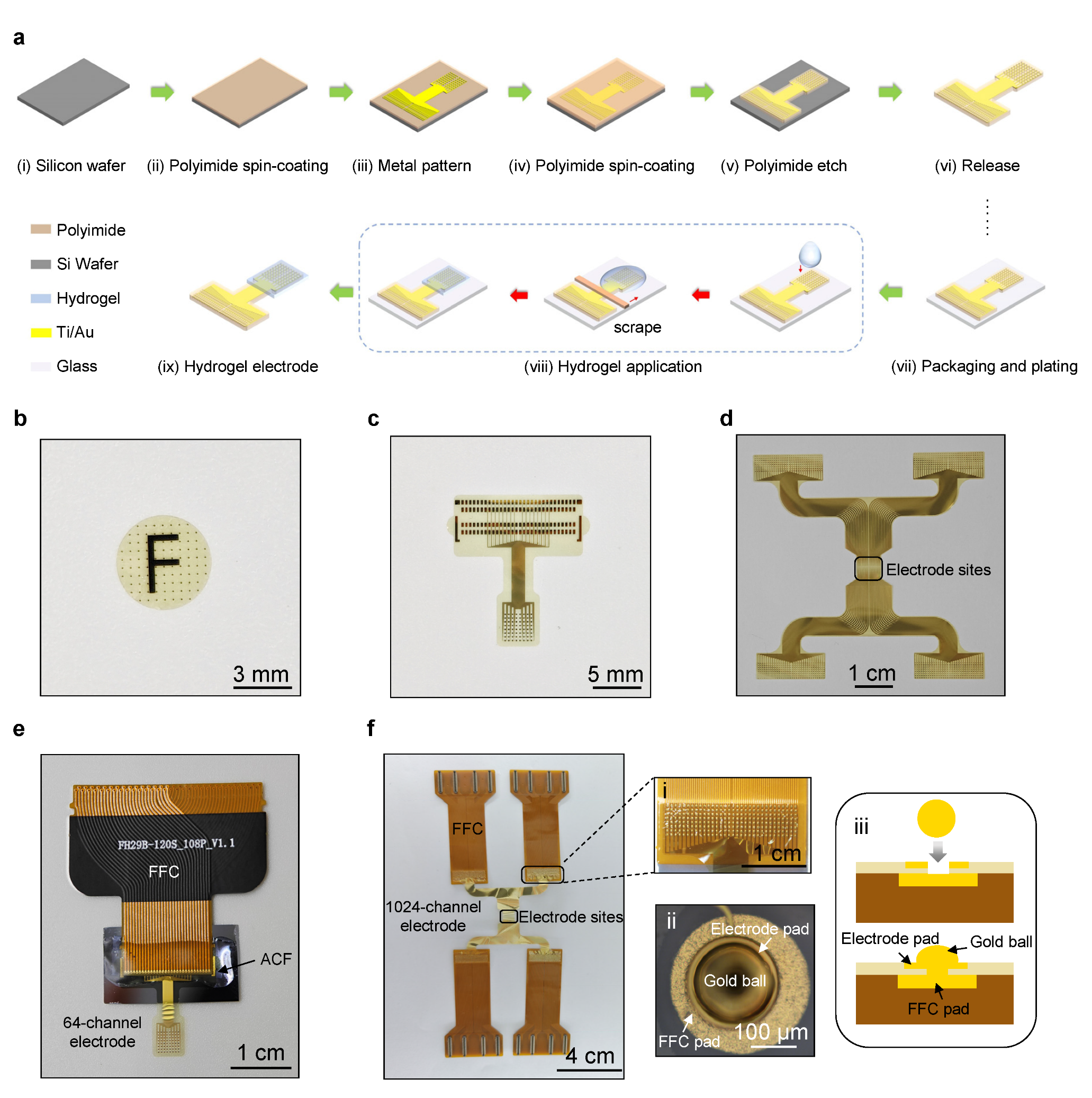


**Figure S11. Detailed fabrication process of aGel-μECoG and photographs of various arrays used in this study. a**) Step-by-step fabrication process of the aGel-μECoG. (i), A 4-inch silicon wafer served as the substrate. (ii), A 3 μm layer of PI was spin-coated onto the silicon wafer. (iii), Metal electrodes and wires were patterned on the substrate using photolithography and thermal evaporation. (iv), A second 3 μm layer of PI was spin-coated onto the initial PI layer. (v), The PI layers were patterned using reactive ion etch (RIE). (vi), The fabricated device was released from the silicon wafer. (vii), The device was packaged using ACF bonding or gold-ball bonding to custom designed FFCs, followed by electroplating if required. (viii), Hydrogel was applied to the arrays, if needed. (ix), The device was released from the glass substrate, completing the fabrication process. **b-d)** Representative photograph of fabricated arrays: disc array (b), 64-channel array (c), and 1024-channel array (d). **e)** Representative photograph of an ACF packaged 64-channel array connected to a custom-designed FFC. **f)** Representative photograph of a 1024-channel array using gold-ball bonding: (i), Gold-ball bonding packaging photograph. (ii), Detailed view of the gold-ball bonding. (iii), Schematic illustration of the gold ball bonding mechanism.


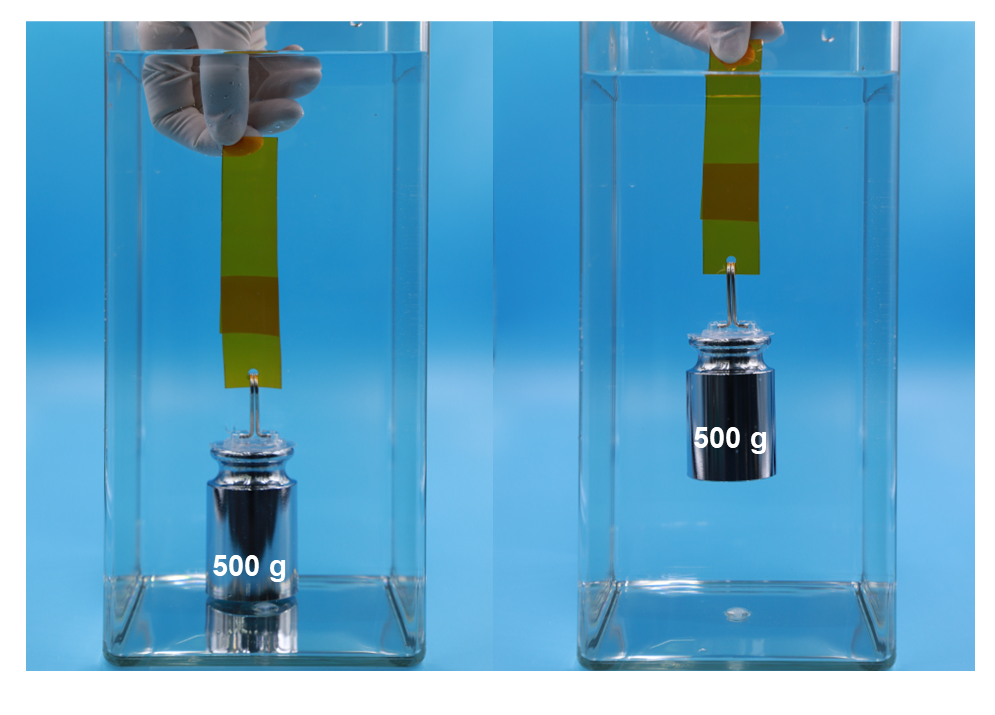


**Figure S12. Demonstration of underwater adhesive strength of aGel-bonded PI films.** The adhesive bond formed through topological entanglement enabled the aGel-bonded PI films to successfully support a 500 g standard weight, illustrating the stability of the adhesive mechanism in underwater environments.


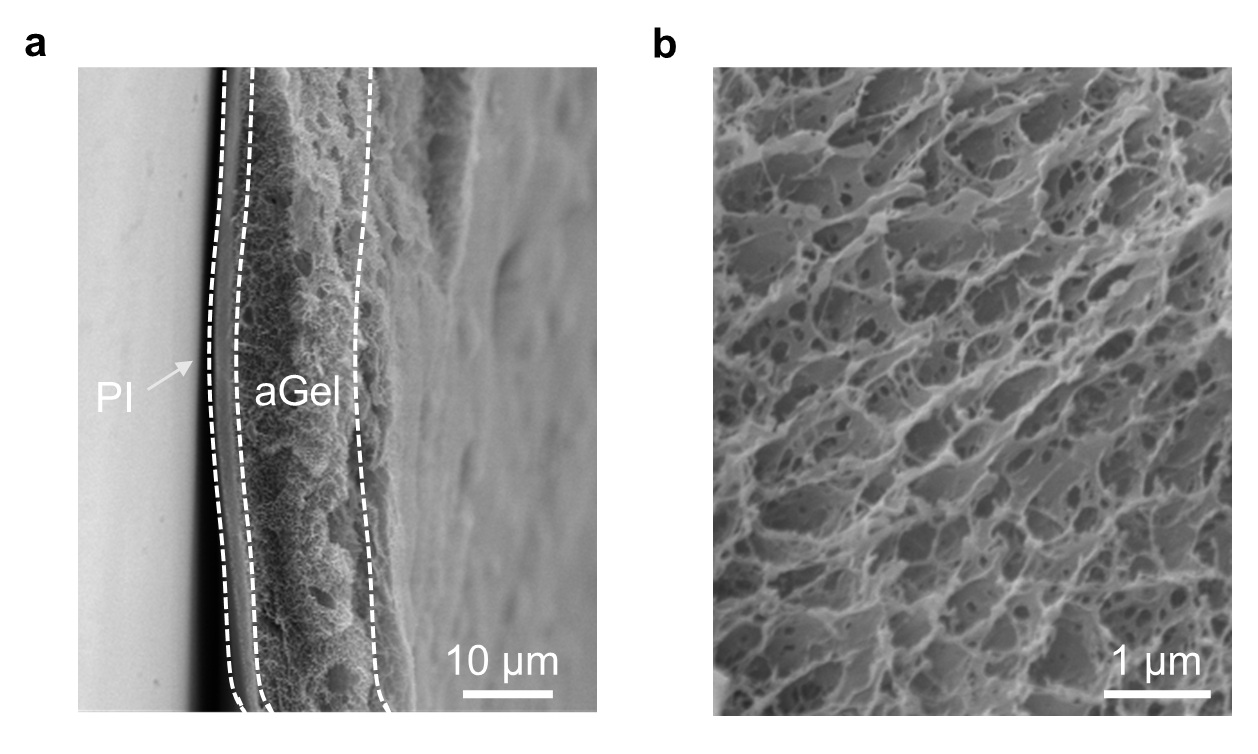


**Figure S13. Cryo-scanning electron microscopy characterization of aGel morphology. a)** Cross-sectional scanning electron microscopy image of the aGel-coated PI interface. The micrographs reveal intimate interfacial contact between the aGel coating and the PI substrate. **b)** Internal morphology and structure of aGel, showing a uniform, loose, and porous microstructure and network organization.


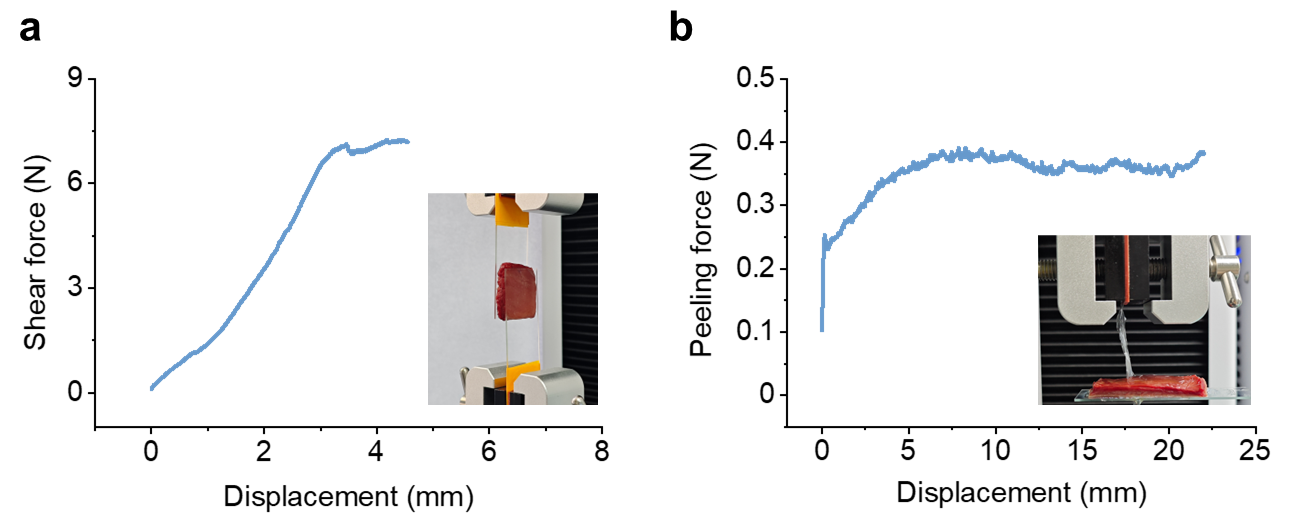


**Figure S14. Mechanical characterization of aGel-tissue interfacial adhesion through lap-shear and peeling tests. a)** Typical adhesive force-displacement curve of aGel-tissue interface obtained from 180-degree lap-shear test, with an inset showing the experimental setup. **b)** Typical adhesive force-displacement curve of aGel-tissue interface obtained from 90-degree peeling test, with an inset showing the experimental setup. The significant difference in shear and peeling adhesion strengths allows for robust adhesion and safe removal from biological tissues.


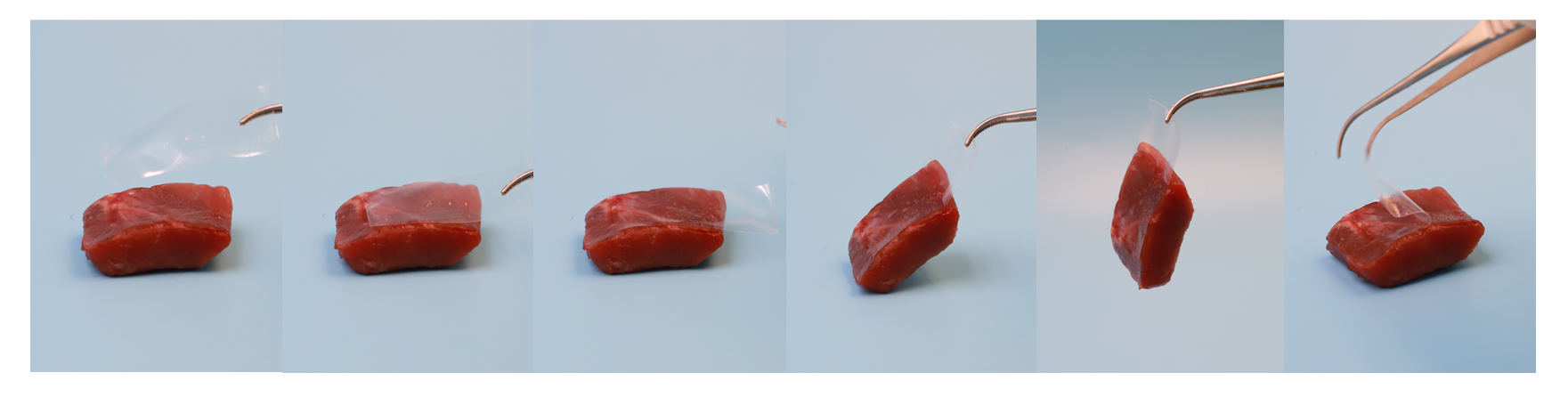


**Figure S15. Photographs of the adhesive process of the aGel (dry state) sheet on pig heart tissue.** Adhesive interaction between the aGel sheet and the heart tissue surface. The experimental observations demonstrated that the aGel sheet established sufficient adhesion to the heart tissue, as evidenced by the elevation of the tissue when the sheet was lifted, indicating strong interfacial adhesion between the aGel sheet and the biological surface.


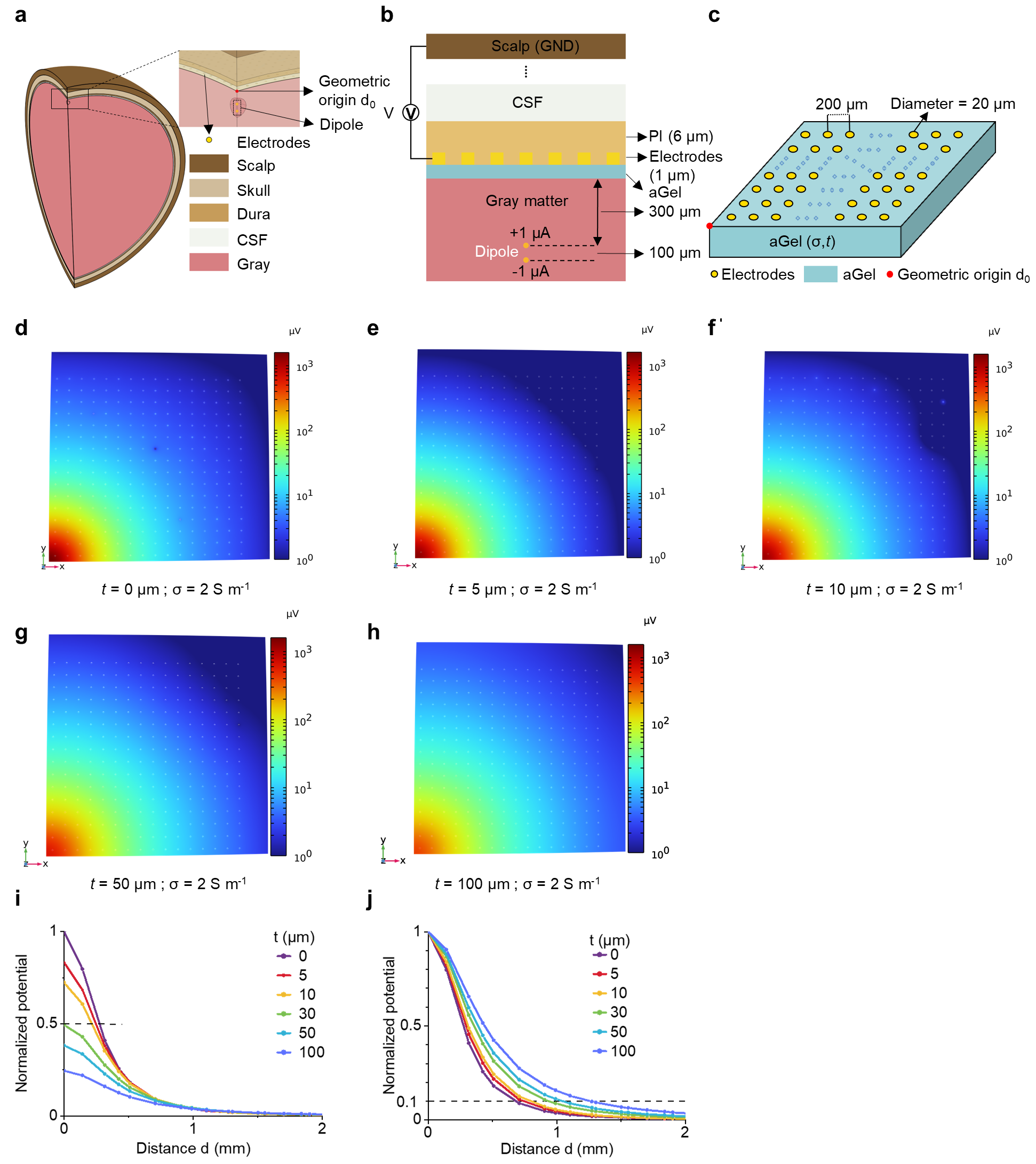


**Figure S16. FE simulations of electrical recordings with varying aGel thickness at fixed conductivity. a)** Full rodent head model showing the position of the dipole source**. b)** Close-up schematic of FE simulation setup. A dipole source was positioned at a depth of 300 μm in the gray matter layer, generating a current of ±1 μA**. c)** Illustration of the 16 × 16 aGel-μECoG array (20 μm diameter, 200 μm pitch). *d_0_* (red dot) represents the geometric origin, where the potential field reaches its maximum under aGel-free conditions. **d-h)** Simulated potential distributions on the electrode plane for aGel layers of different thicknesses (t) with fixed conductivity (σ = 2 S m^-1^). **i)** Potential decay profile versus distance *d* extracted from (d) to (h). Distance d was calculated using the Euclidean distance from each electrode to *d_0_*. **j)** Normalized potential decay profiles as a function of electrode distance *d* for fixed σ = 2 S m^-1^ across varying *t*, with spatial resolution defined by the position where the normalized potential drops to 0.1.


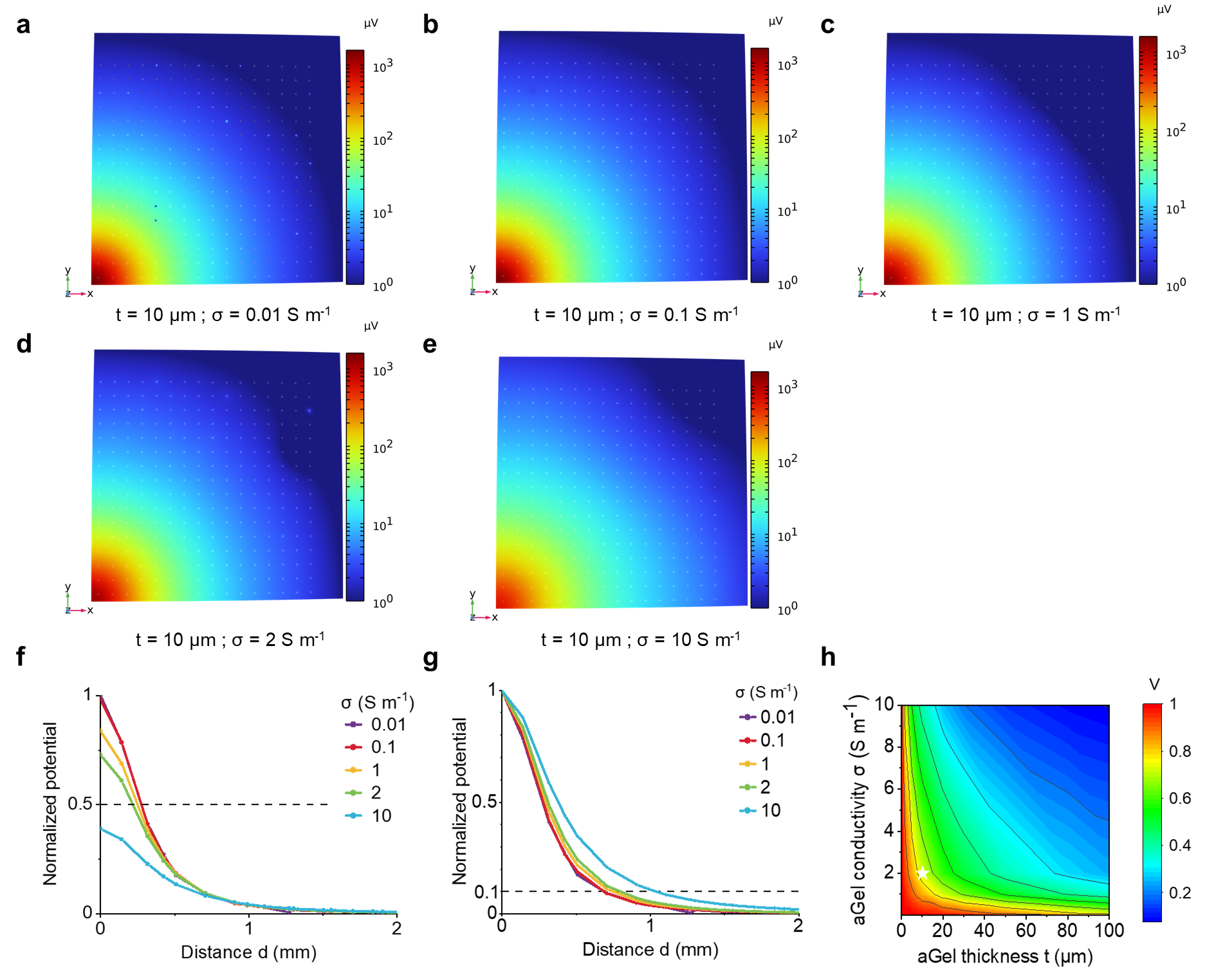


**Figure S17. FE simulations of electrical recordings with varying aGel conductivity at a fixed thickness. a-e),** Simulated potential distributions on the electrode plane for aGel layers of different conductivities (σ) with fixed thickness (*t* = 10 μm). **f)** Normalized potential decay profile versus distance *d* extracted from (a) to (e). **g)** Normalized potential decay profiles as a function of electrode distance *d* for fixed *t* = 10 μm across varying σ, with spatial resolution defined by the position where the normalized potential drops to 0.1. **h)** Normalized iso-potential map showing the effects of varying *t* (0-100 μm) and σ (0.01-10 S m^-1^). The potential (*V*) was normalized to the value at *d*_0_ under aGel-free conditions. The white star indicates the optimal design parameter chosen for this study (*t* ≈ 10 μm, σ ≈ 2 S m^-1^).


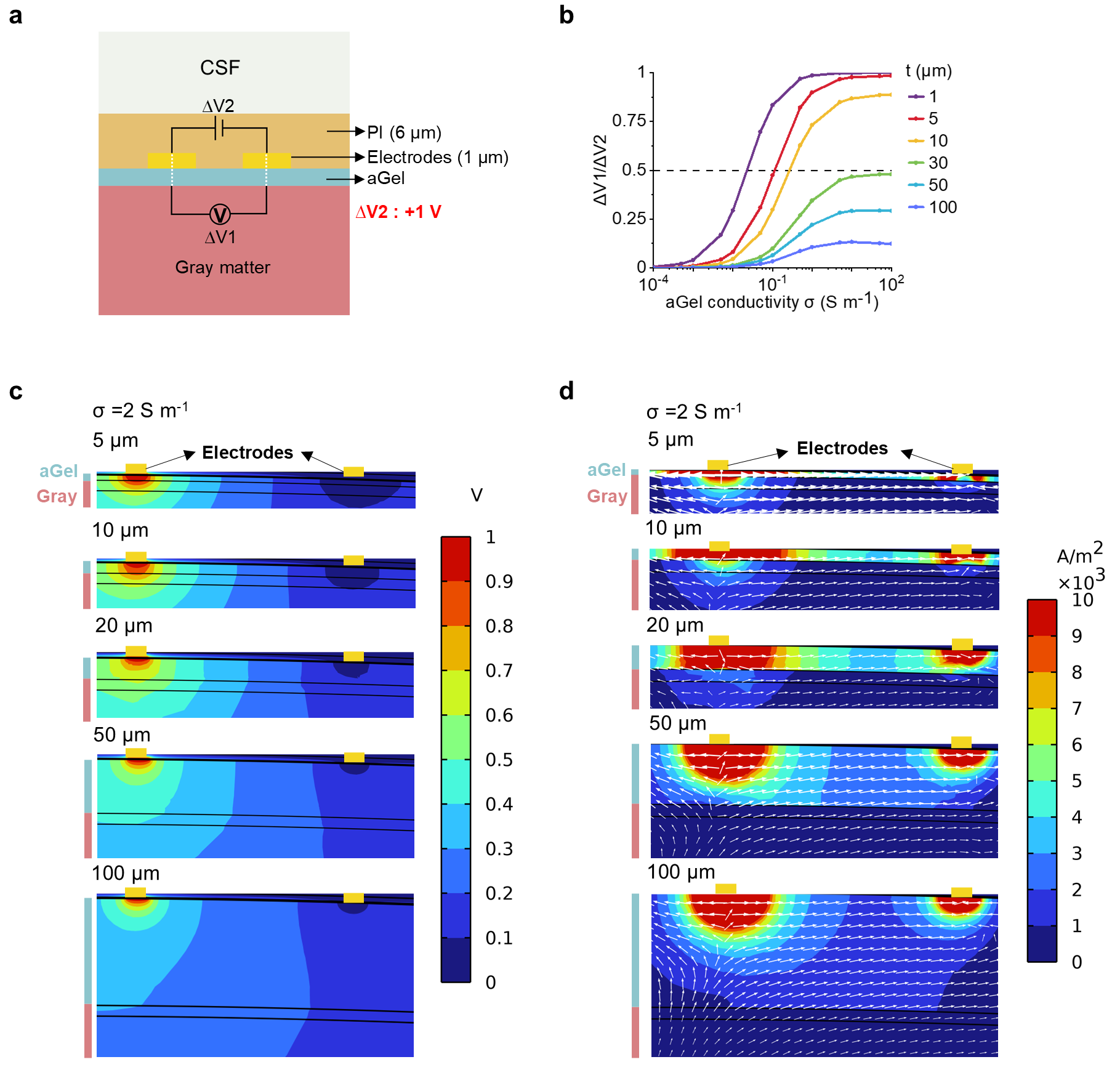


**Figure S18. FE simulations of electrical stimulation with varying aGel thickness. a)** FEM simulation diagram, electrode provides +1 V voltage stimulation signal. **b)** Representative energy transfer ratio curve by sweeping σ across varying thickness *t* (*t* = 5, 10, 20, 50, and 100 μm). **c)** Potential distribution resulting from the above setup of varying thickness *t* (*t* = 5, 10, 20, 50, and 100 μm), with a fixed aGel conductivity σ of 2 S m^-1^. **d)** Corresponding current density distribution for the same conditions as shown in (c).


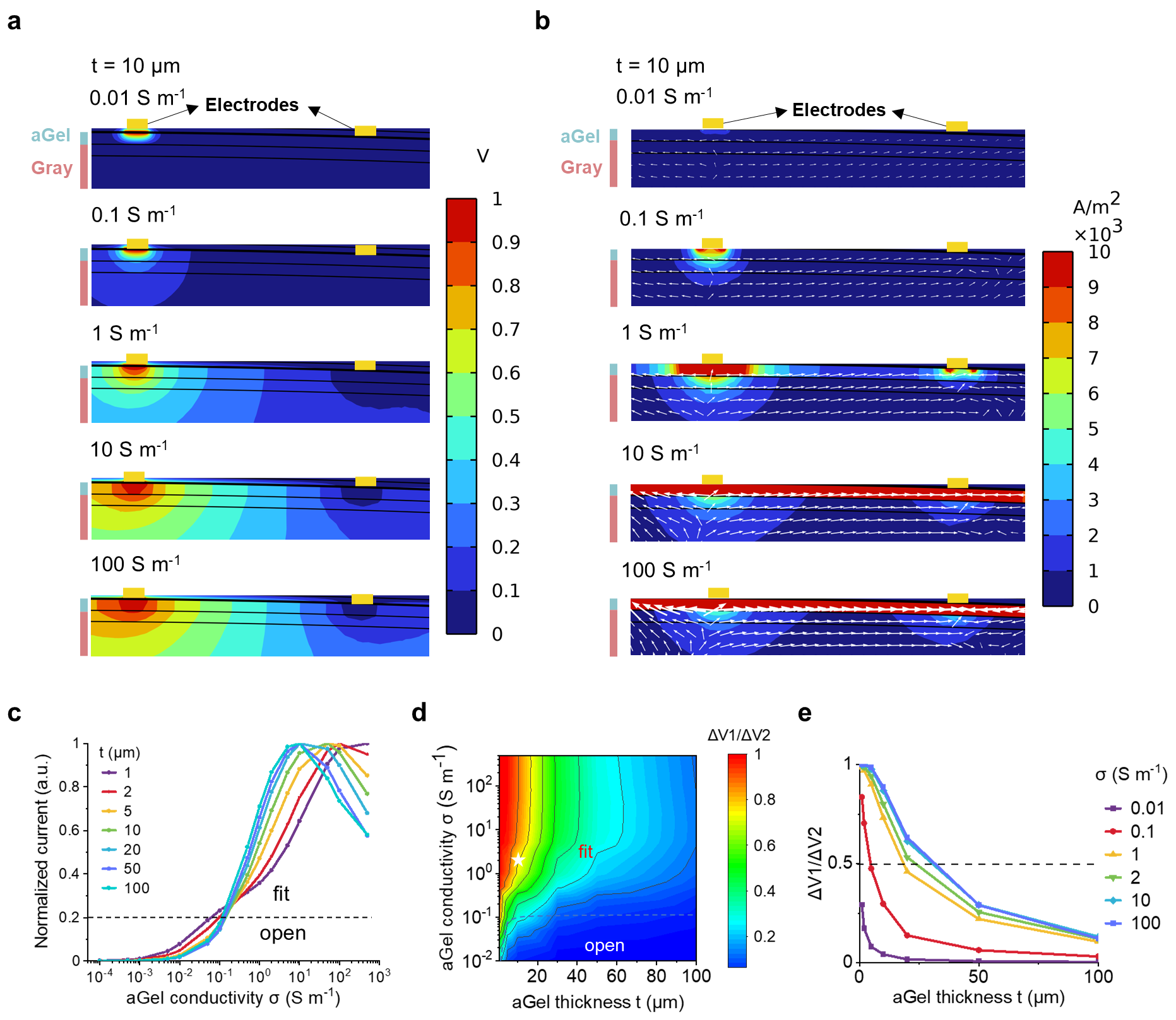


**Figure S19. FE simulations of electrical stimulation with varying aGel conductivities. a)** Simulated potential distribution under a constant voltage stimulation of 1 V for aGels with different conductivities (σ = 0.01, 0.1, 1, 10, and 100 S m^-1^), with the hydrogel thickness t fixed at 10 μm. **b)** Corresponding current density distribution for the same conditions as shown in (a). **c)** The Normalized current in the gray tissue extracted from (b). A normalized current value of 0.2 was regarded as an open circuit in the analysis. **d)** Energy transfer ratio map from the parametric sweep of t and σ. The white star indicates the optimal design parameter chosen for this study (*t* ≈ 10 μm, σ ≈ 2 S m^-1^). **e)** Representative energy transfer ratio curves for sweeping hydrogel thickness *t* across varying σ (σ = 0.01, 0.1, 1, 10, and 100 S m^-1^).


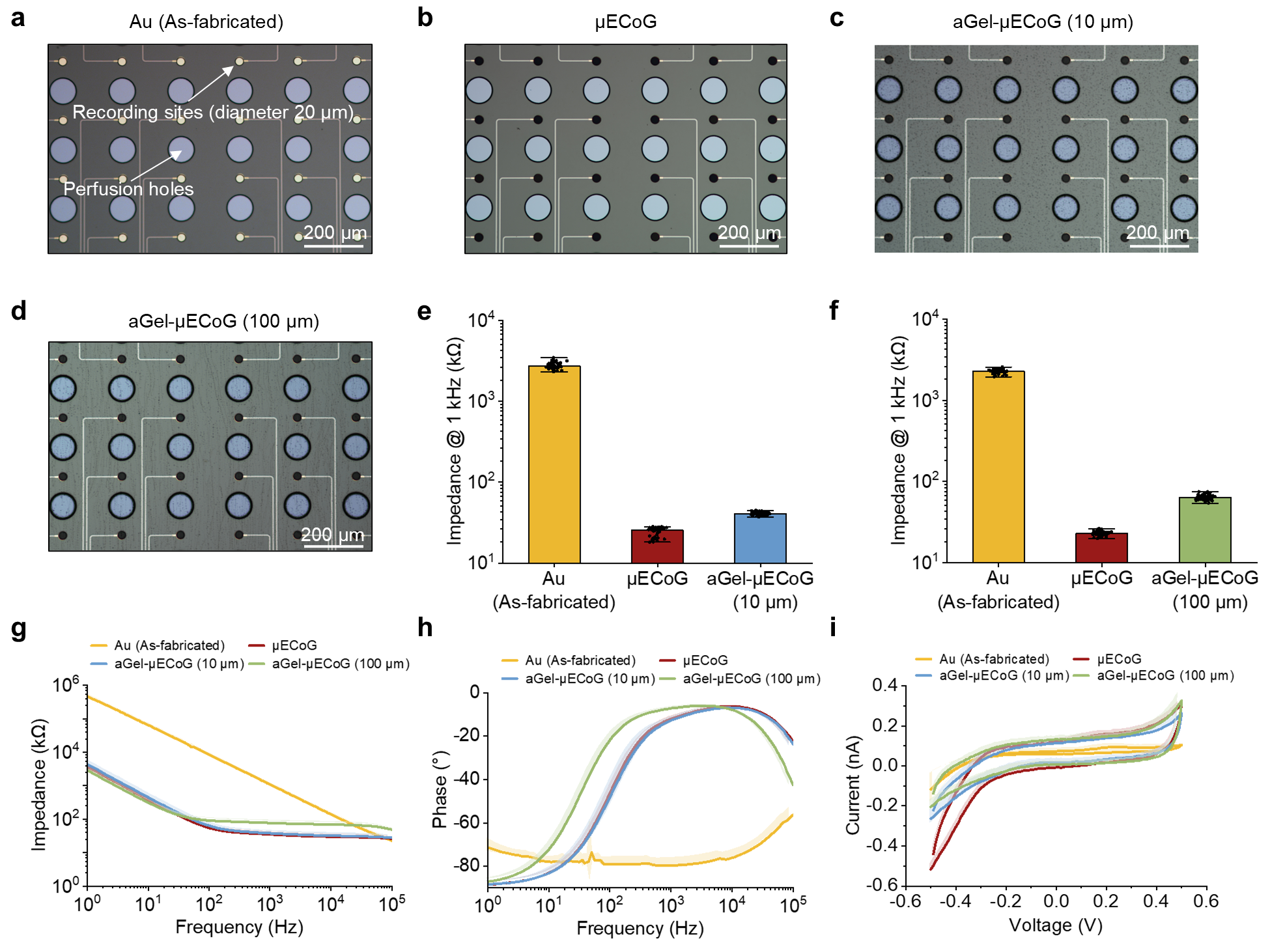


**Figure S20. Electrical characterization of 1024-channel arrays. a-d)** Representative microscopic photographs of the Au (a), μECoG (b), aGel-μECoG (10 μm) (c), and aGel-μECoG (100 μm) (d) arrays. **e)** 1 kHz impedance for Au, μECoG, and aGel-μECoG (10 μm) arrays. **f)** 1 kHz impedance for Au, μECoG, and aGel-μECoG (100 μm) arrays. **g,h)** Impedance (g) and phase (h) measurements for Au, μECoG, aGel-μECoG (10 μm), and aGel-μECoG (100 μm) arrays. **i)** CV measurements for Au, μECoG, aGel-μECoG (10 μm), and aGel-μECoG (100 μm) arrays.


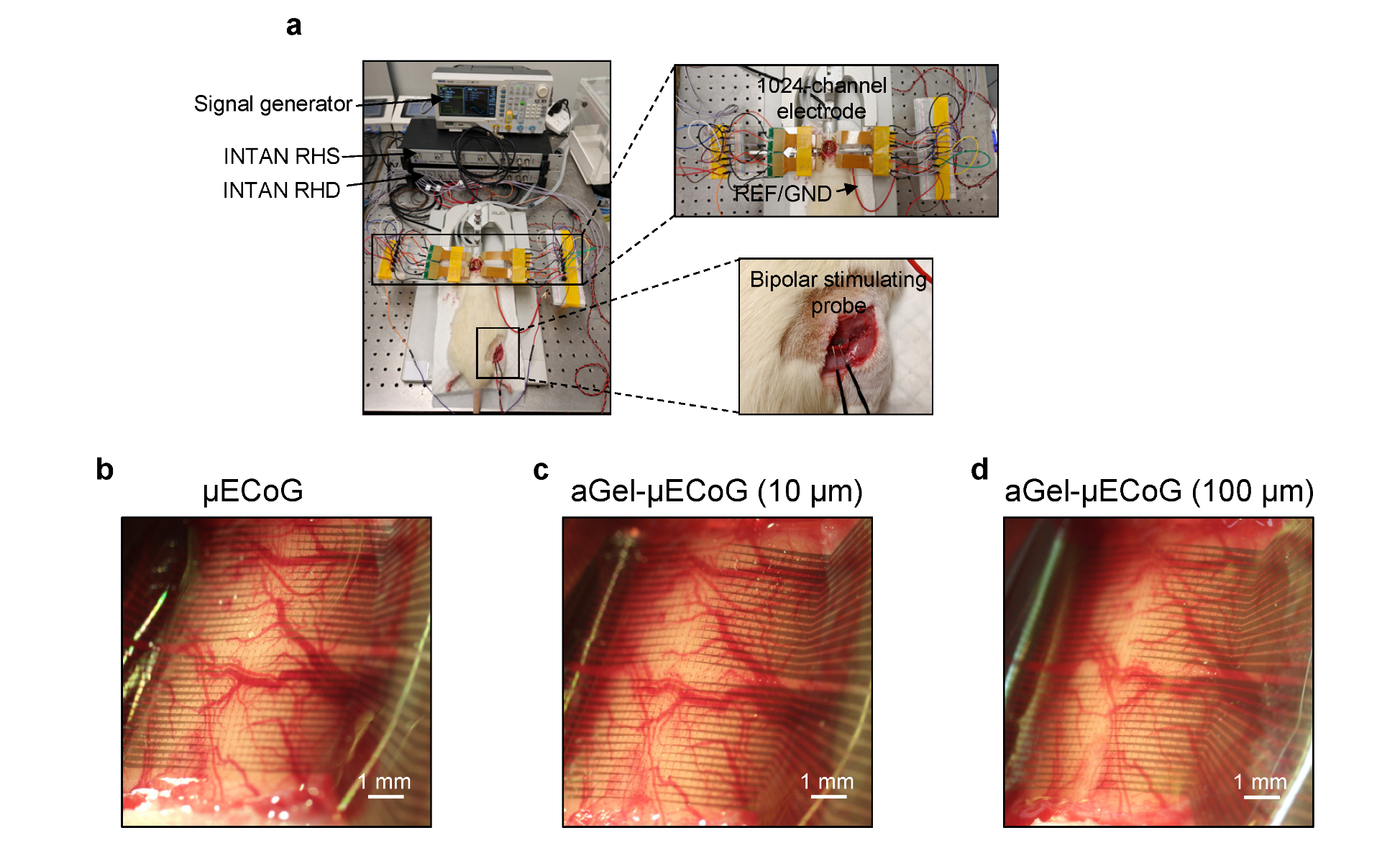


**Figure S21. Anesthetic test photographs of 1024-channel devices during functional cortical mapping. a)** Photograph of the intraoperative test setup. **b-d)** Representative photos of various 1024-channel arrays conformally attached to the rat cortical surface: μECoG (b), aGel-μECoG with a 10 μm hydrogel layer (c), and aGel-μECoG with 100 μm hydrogel layer (d).


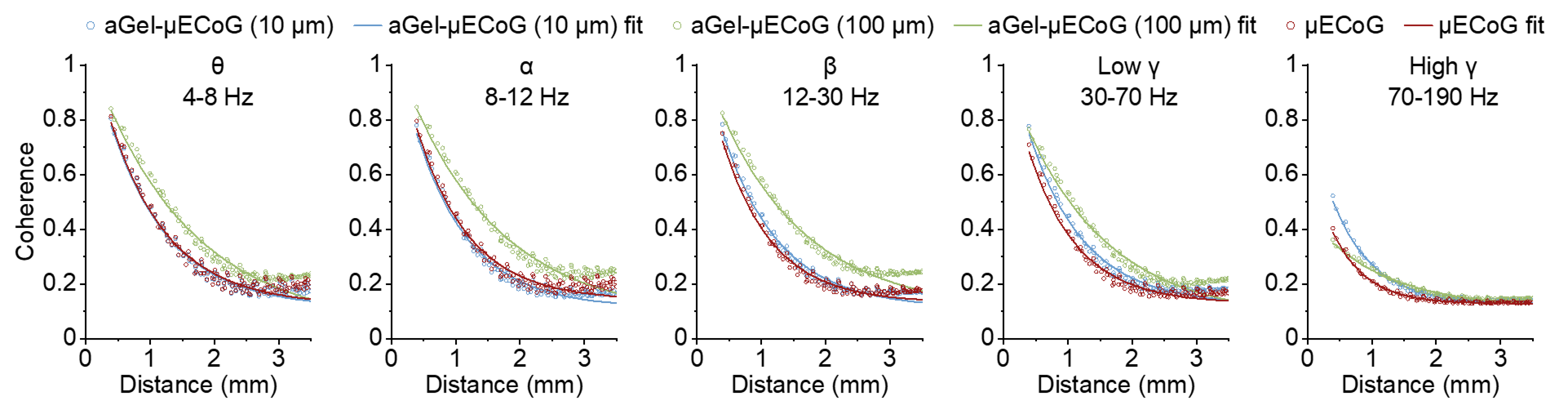


**Figure S22. Spatial resolution analysis.** Inter-electrode coherence-distance relationship for the high-density 1024-channel μECoG across frequency bands (θ, α, β, low γ, and high γ) (curve fitting: y = a∙exp(-x/λ) + c).


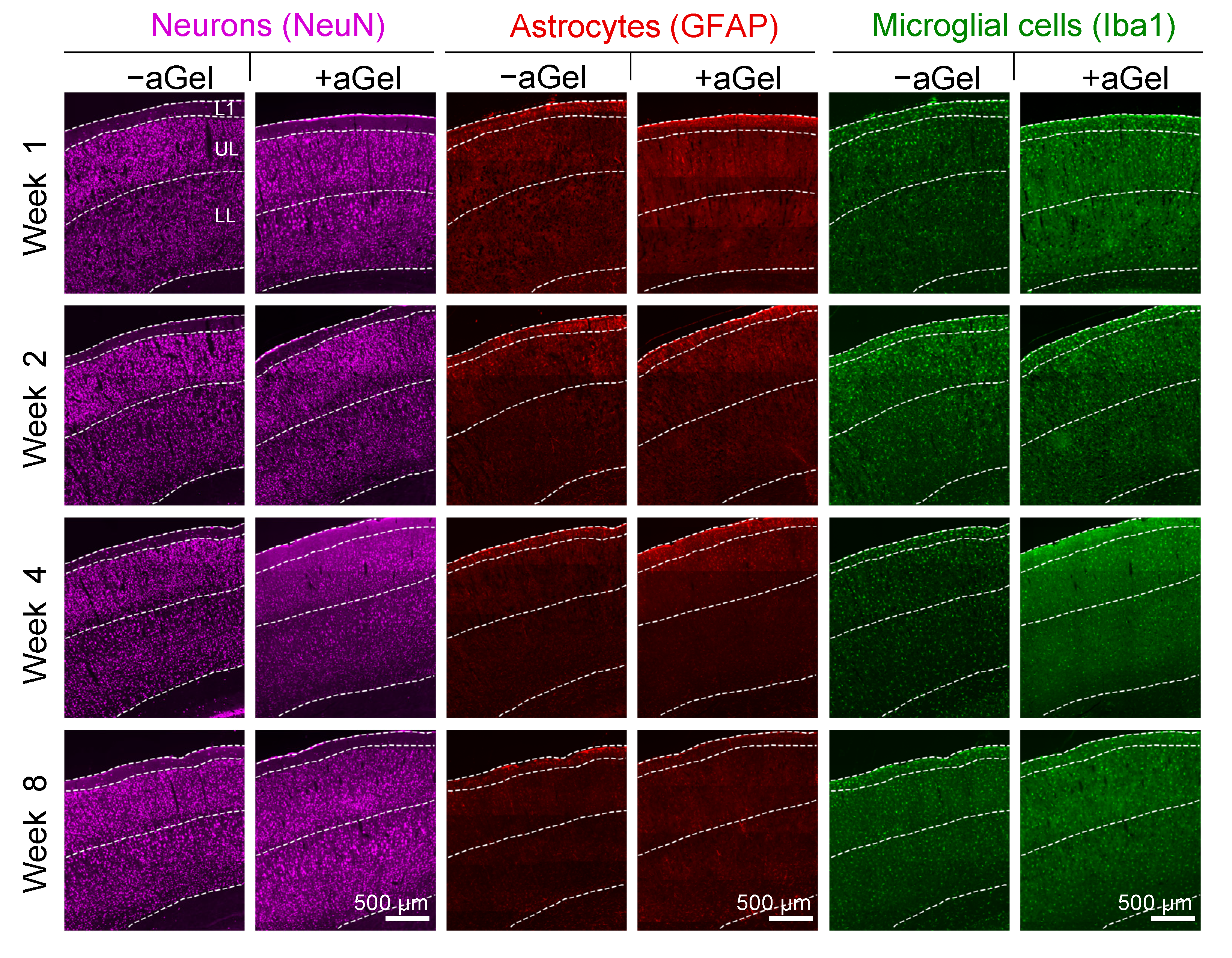


**Figure S23. Fluorescence images of the control side.** Representative immunofluorescence images of the control side at various time points after the implantation of −aGel and +aGel. The cortex was divided into L1, UL, and LL based on the neuronal density and distribution characteristics.


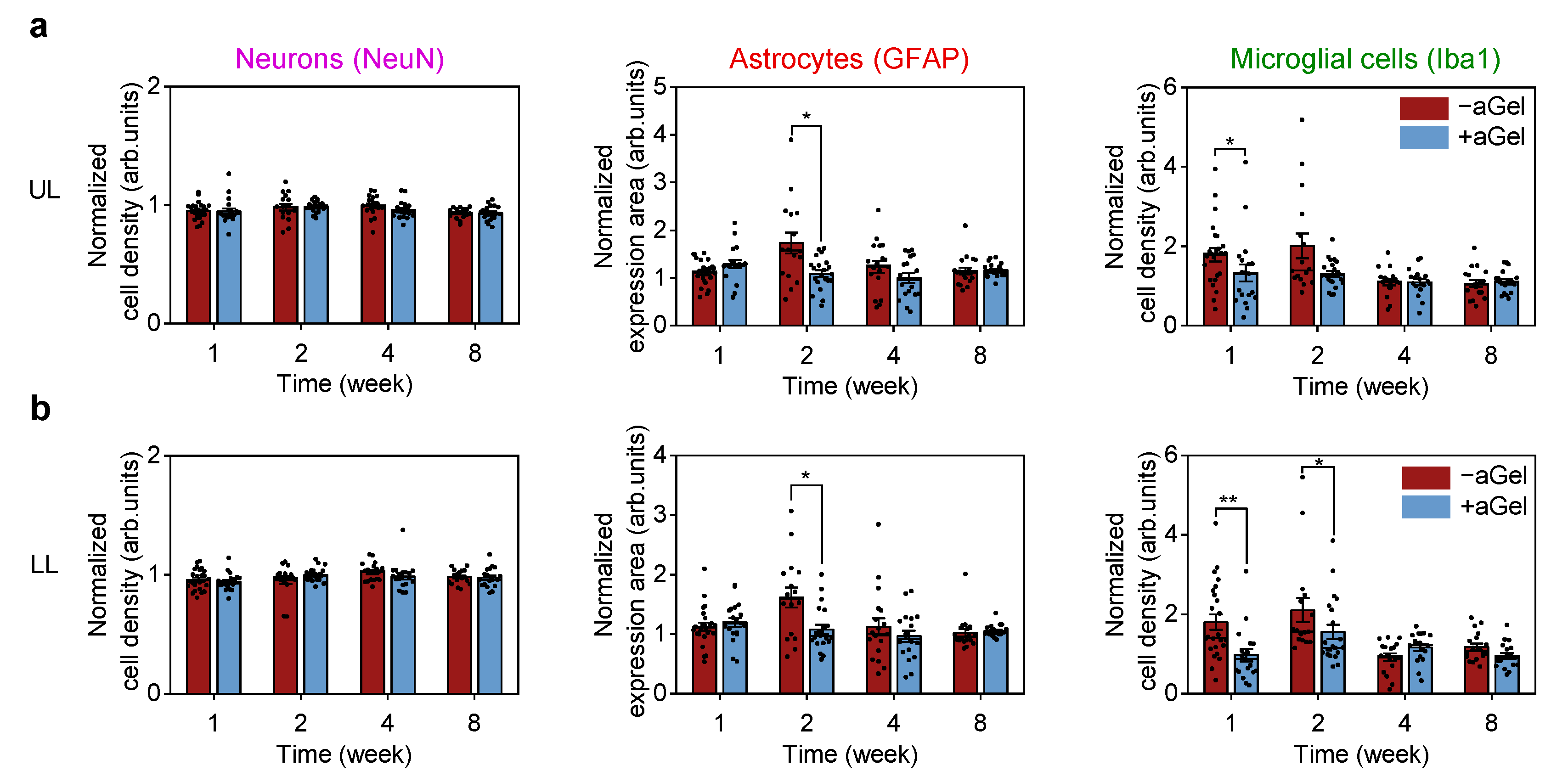


**Figure S24. Quantitative statistical analysis of the UL and LL. a,b)** Quantitative statistical analysis of NeuN, GFAP, and Iba1 expression in UL (a) and LL (b) at various time points after the implantation of −aGel and +aGel. Data from the implant side were normalized to the contralateral control side for both the −aGel and +aGel groups (n = 24, 19 from five rats for week 1; n = 16, 21 from three rats for week 2; n = 18 from three rats for week 4; n = 18 from three rats for week 8). *p* = 0.0113 (GFAP UL, week 2), *p* = 0.0175 (GFAP LL, week 2), *p* = 0.0252 (Iba1 UL, week 1), *p* = 0.0014 (Iba1 LL, week 1), *p* = 0.0224 (Iba1 LL, week 2). Data are presented as mean ± SEM. **p* < 0.05, ***p* < 0.01, ****p* < 0.001.


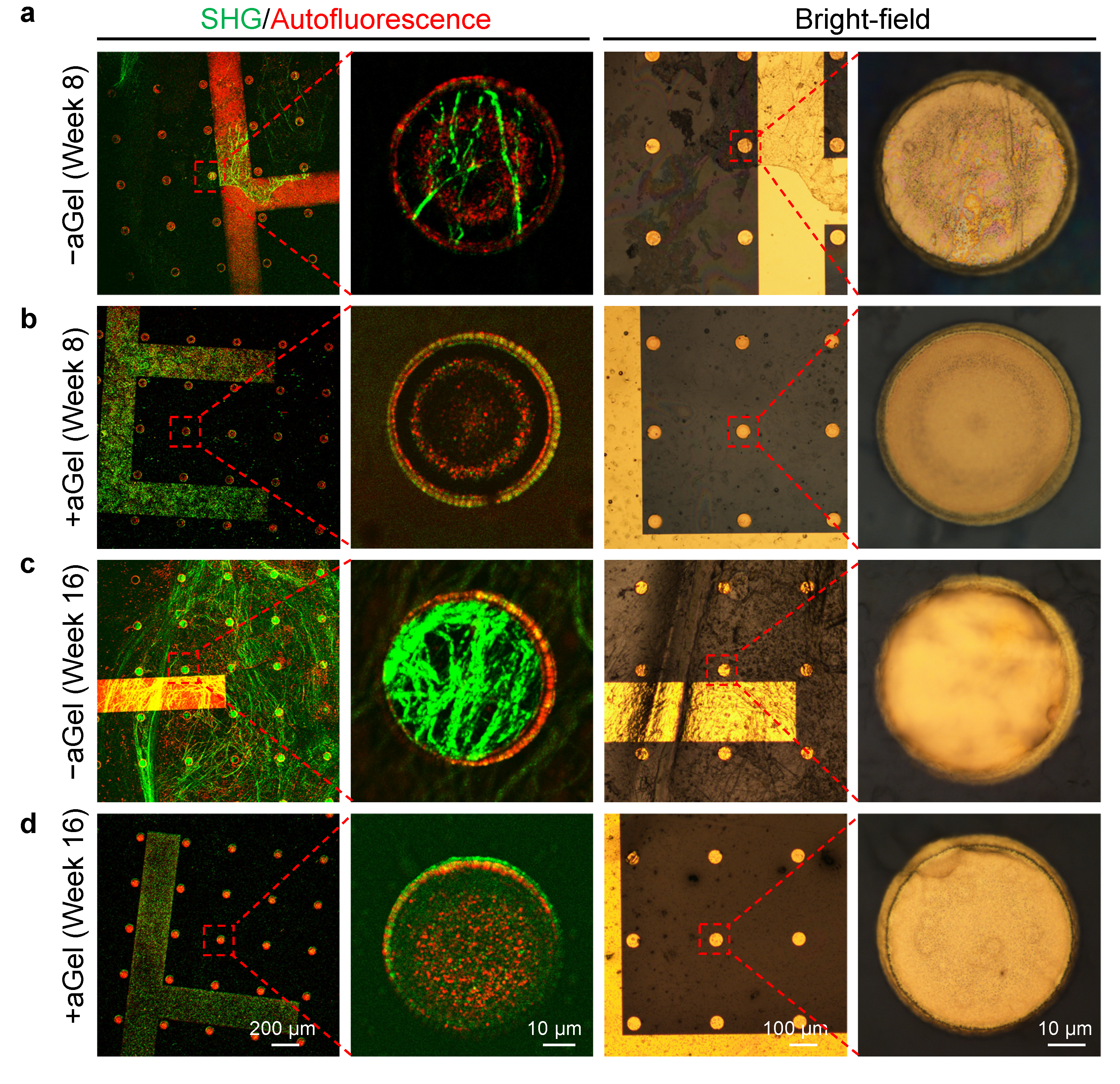


**Figure S25. SHG images of −aGel and +aGel films**. **a-d)** Representative images of −aGel (a, c) and +aGel (b, d) films taken after 8 weeks (a, b) and 16 weeks (c, d) of in vivo implantation. The SHG images were captured using a Nikon two-photon microscope, with green and red representing the SHG signal and spontaneous fluorescence, respectively. Bright-field optical photographs were also taken under white light. Enlarged images of both SHG and bright-field photographs are positioned on the right side.


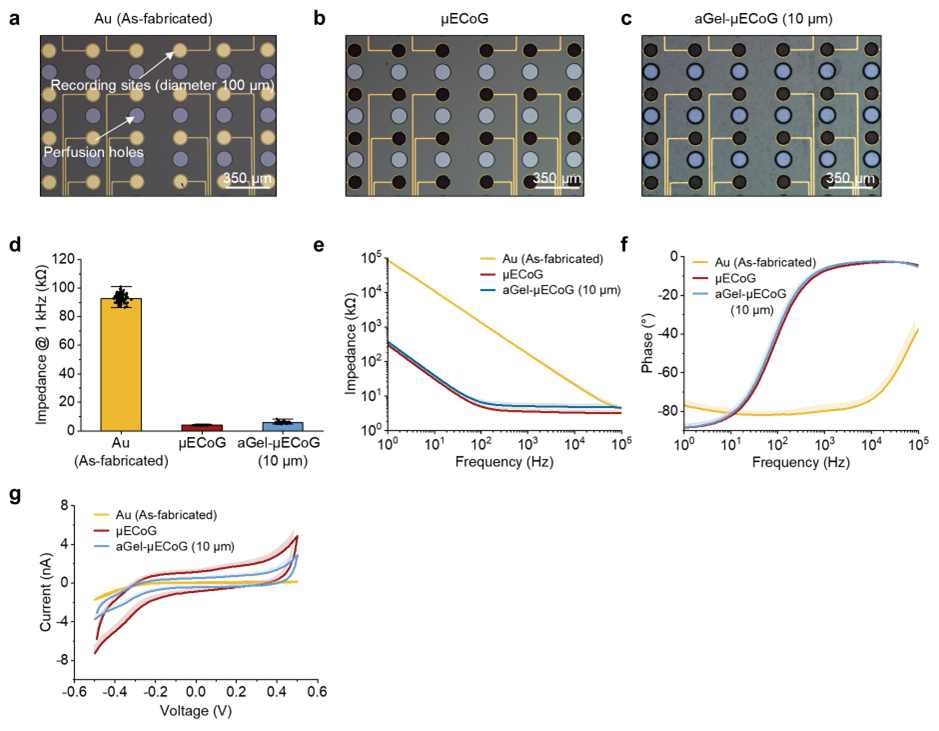


**Figure S26. Electrical characterization of 64-channel arrays. a-c)** Representative microscopic photographs of Au (a), μECoG (b), and aGel-μECoG (10 μm) (c) arrays. **d)** 1 kHz impedance for Au, μECoG, and aGel-μECoG (10 μm). **e,f)** Impedance (e) and phase measurements of Au, μECoG, and aGel-μECoG (10 μm) arrays. **g)** CV measurements of Au, μECoG, and aGel-μECoG (10 μm) arrays.


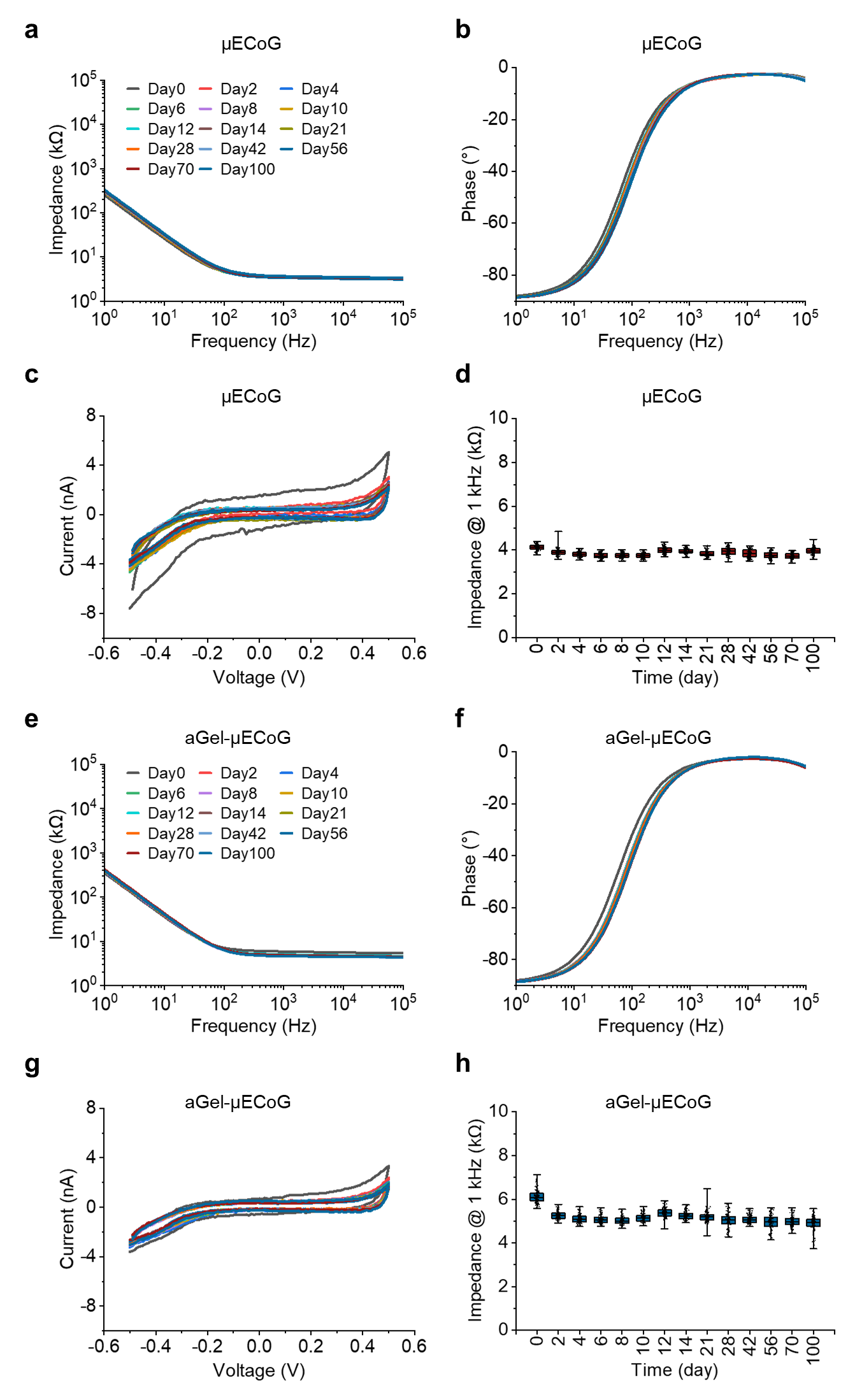


**Figure S27. Aging Experiments. a-d)** EIS (a, b), CV (c), and impedance at 1 kHz (d) of μECoG immersed in PBS at 60°C, measured at 0, 2, 4, 6, 8, 10, 12, 14, 28, 42, 56, 70, and 100 days. **e-h)** EIS (e, f), CV (g) and Impedance at 1 kHz (h) of aGel-μECoG immersed in PBS at 60°C, measured at 0, 2, 4, 6, 8, 10, 12, 14, 28, 42, 56, 70, and 100 days. Box plots display the median, maximum, minimum, and interquartile range, with individual data points overlaid. n = 118 for μECoG; n = 108 for aGel-μECoG.

**
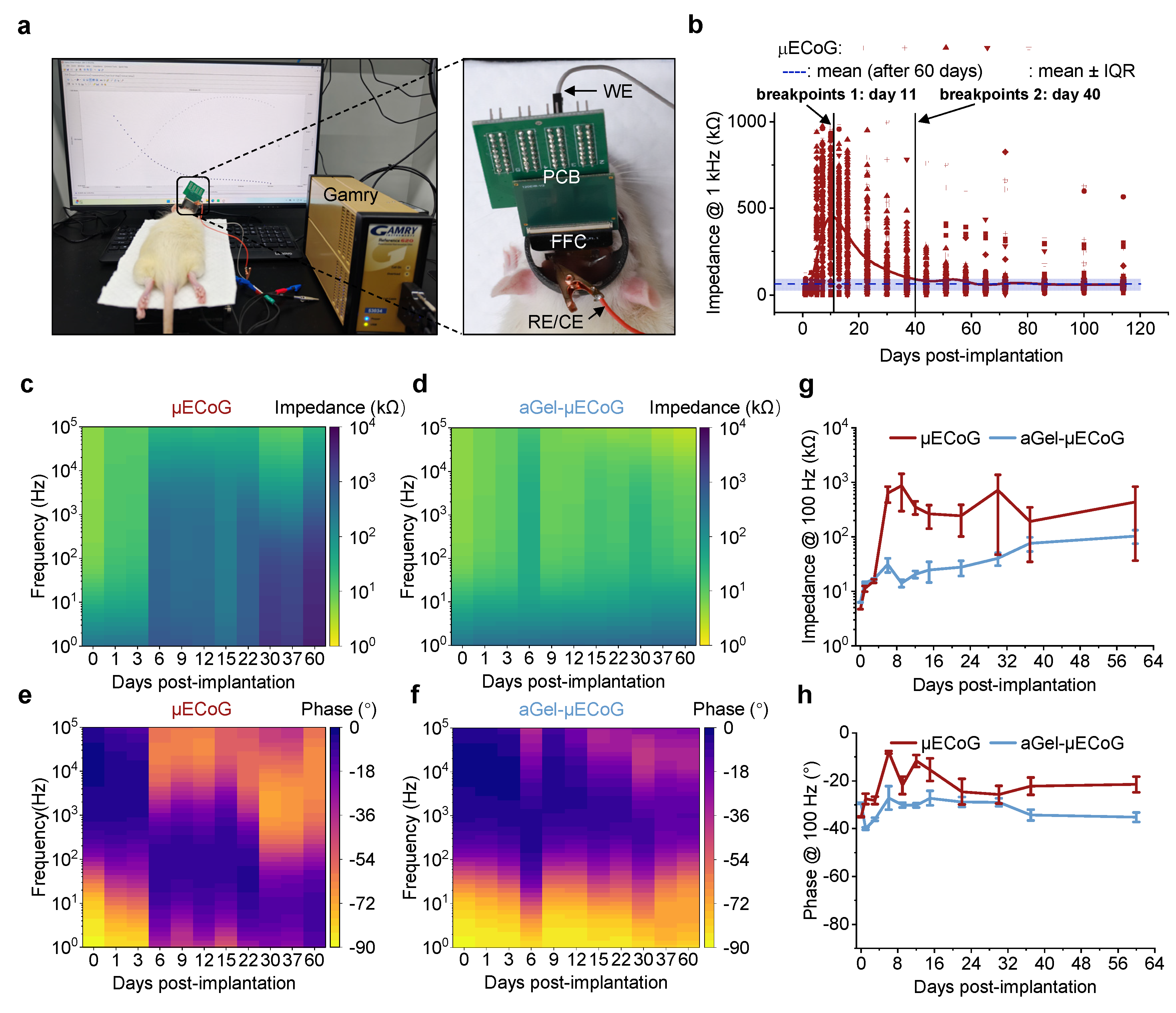
**

**Figure S28. Impedance nonlinearity trends and EIS changes across a 60-day implantation period. a)** Experiments setup for in vivo EIS measurements. **b)** Nonlinear variation trend of electrode impedance of μECoG over time modeled using GAM. The impedance initially increased, peaking on day 11 (breakpoint 1) at 451.1 kΩ, followed by a decreased until day 40 (breakpoint 2), where it stabilized at 91.8 kΩ, and subsequently remained within the range of 70.4 ± 1.2 kΩ. **c,d)** Impedance changes across a 60-day implantation period for μECoG (c) and aGel-μECoG (d). **e,f)** Phase changes during the 60-day implantation period for μECoG (e) and aGel-μECoG (f). **g)** Comparison of impedance at 100 Hz between μECoG and aGel-μECoG. **h)** Comparison of phase at 100 Hz between μECoG and aGel-μECoG. Data are presented as mean ± SEM. n = 6 from two rats for each testing day.


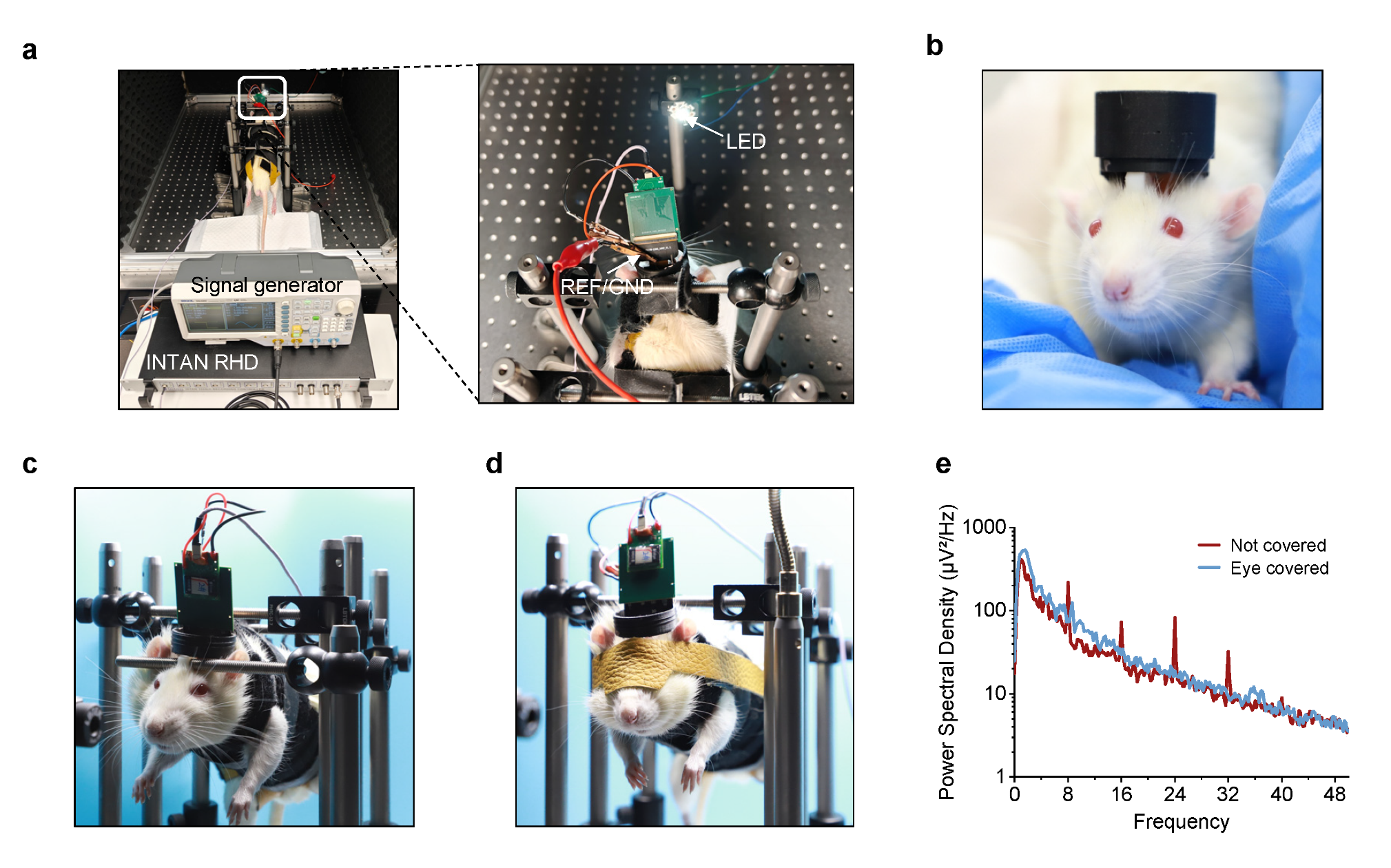


**Figure S29. Experimental setup for SSVEP recording in awake rats**. **a)** Photograph of the SSVEP recording setup, showing the complete experimental details. **b)** A rat two months post-implantation with the electrode connector protected by a custom-designed black cap. The cap was created using 3D printing, and half of its base was removed to allow a stub to pass through the nut, as shown in panel (c). **c)** The rat was elevated and its head was fixed, with the headstage connected to the electrode for stable recording. **d)** The rat’s eye was covered with a belt during recording. **e)** Comparison between eye-covered and non-covered SSVEP recording results, demonstrating that the recorded signal originated from the brain rather than interference from signal generator or LED.


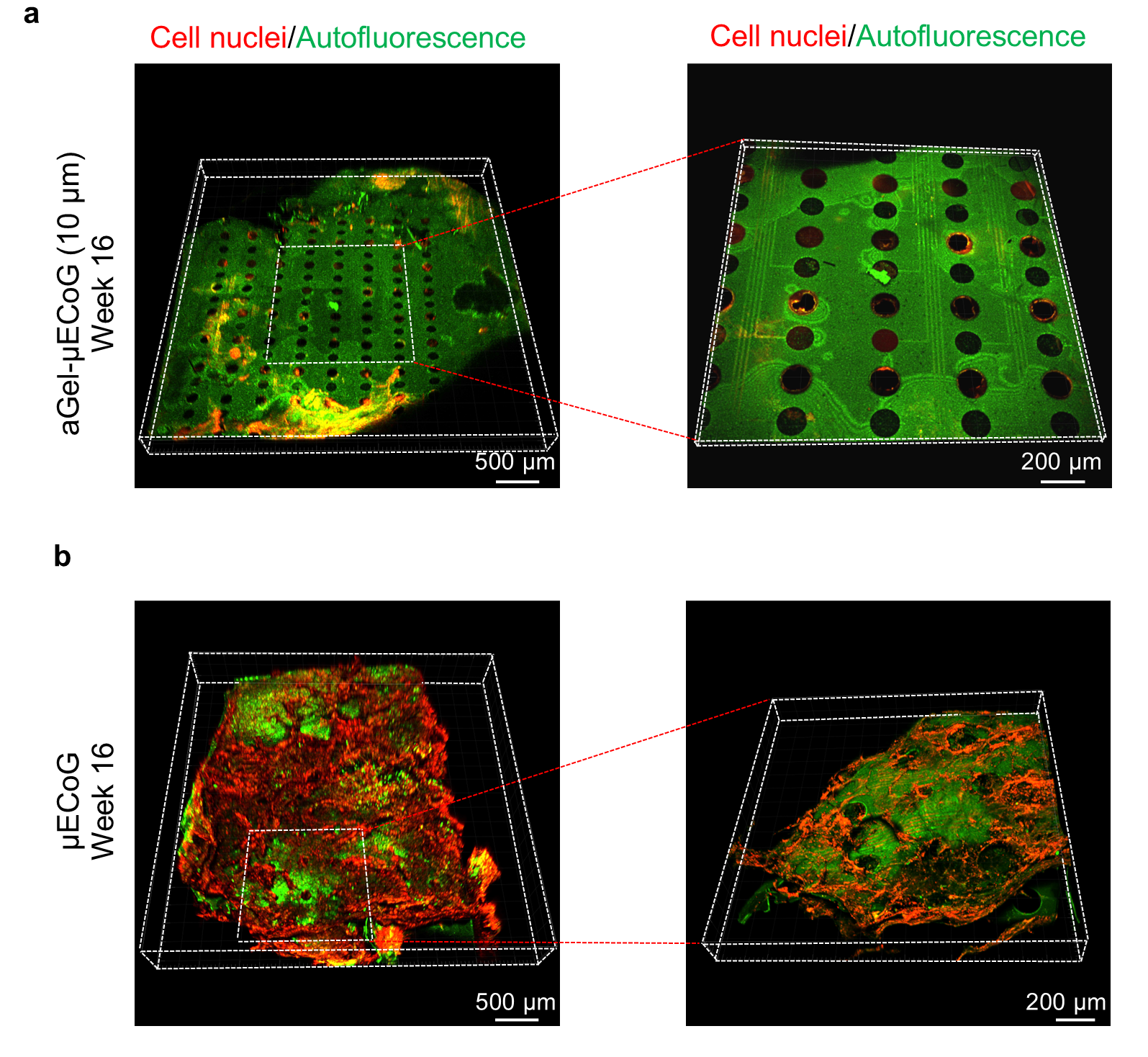


**Figure S30. Fluorescence images of cell nuclei stained with propidium iodide. a,b)** Representative fluorescence images of the aGel-μECoG electrode (a) and μECoG electrode (b) stained with propidium iodide, with red representing the cell nuclei and green representing spontaneous fluorescence, the enlarged image is positioned on the right side.

**Table S1.** Comparison of aGel-μECoG with other recently published technologies using hydrogel as an interfacial layer. aGel-μECoG achieved stable chronic recordings for up to 16 weeks.

| Hydrogel/  Substrate/  Electrode Materials | # of Chan./Integrated? (Y/N) | t (µm)/  σ (S m^-1^) | Adhesion mechanism to biological tissues | Physical bond only? (Y/N) | Adhesion strength | Animal/organ/recording duration/Signal type | Chronic recording  (Y/N) | Refs. |
| --- | --- | --- | --- | --- | --- | --- | --- | --- |
| PVA-PTPM/  PI/  PEDOT: PSS | 64, 1024/Y | 10/1.99 | Hydrogen bonds | Y | 25.2 ± 3.8 kPa | 1.Rats/subdural/16 weeks/ECoG (SSVEP)  2.Rats/subdural/acute/ECoG (motor cortical signals) | Y/16 weeks | This work |
| Alg-CA/  PI/SHP  Au | 16/Y | ~10 µm/Ionic conductive | Covalent (catechol)+ Hydrogen bonds | N | 23.9 kPa | 1.Rats/epidural/acute/ECoG  2.Rats/epidural/24 weeks/ECoG (epileptic signals) | Y/24 weeks | [37] |
| F127DA-PSBMA-PAA-PVB/  SU8/  PEDOT | 128/N | 150  (depth of mold)/NA | Covalent (EDC/NHS) + Hydrogen bonds | N | 22.94 kPa | 1.Rats/subdural/7 days/  ECoG (epileptic signals) | Y/7 days | [36] |
| dPEDOT-CA-PDA-PAM/  PI/PDMS/  Au | 4/Y | NA/42.0 ± 0.6 | Covalent (catechol) + Hydrogen bonds | N | ~3.5 kPa | 1. Rats/epidural/  acute ECoG (SSVEP)  2.Human/forehead/acute/ EEG&EOG | N | [46] |
| PVA/  PDMS/  PEDOT | 2/Y | 1000/~90% of saline | Hydrogen bonds | Y | NA | 1.Rats/subdural/  acute/ECoG (epileptic signals)  2.Porcine/brain (subdural)/  acute/ECoG | N | [32] |
| PAA-PVA/  PU/  Au | 2/N | 100/NA | Covalent (NHS)+ Hydrogen bonds | N | ~170 kPa | 1.Rats/heart/84 days/ECG | Y/84 days | [41] |
| PEG-LA-DA/SA (BTIM)/  Parylene/  Au | 64/Y | 500/~0.5 | Covalent bonds  (chitosan+EDC/Sulfo-NHS) + Physical  entanglement | N | 240 ± 20 Jm^−2^ | 1.Rabbit/heart/(langendorff)/acute/ECG | N | [42] |
| Alg-CA/  E-SHN/  EGaIn composite | 4/N | 13.8  (solid film)/Ionic conductive | Covalent (catechol)+ Hydrogen bonds | N | 7.2 kPa | 1.Rats/heart/4 weeks/ECG  2.Rats/heart/acute/ECG | Y/4 weeks | [40] |
| pCB-pSB (ZEN hydrogel) | NA | 1000 (disk)/NA | Hydrogen bonds | Y | NA | NA | NA | [43] |

**Table S2.** Summary of the conductivity and layer thickness in the finite element model of the rat brain.^[46]^

| **Layer name** | **Thickness (mm)** | **Conductivity (S m^-1^)** |
| --- | --- | --- |
| Scalp | 0.5 | 0.465 |
| Skull | 0.5 | 0.02 |
| Dura | 0.1 | 0.065 |
| CSF | 0.1 | 1.79 |
| Gray | 5.0 | 0.27 |
| PI | 0.006 | 10^-14^ |

| Day | aGel-μECoG (5 rats) | | | μECoG (5 rats) | | |
| --- | --- | --- | --- | --- | --- | --- |
|  | Impedance (kΩ)  (Change compared  to day 1) | Slope  (kΩ d^−1^) | *p* | Impedance (kΩ)  (Change compared  to day 1) | Slope  (kΩ d^−1^) | *p* |
| 1 | 19.7 ± 3.8 | 0.8 | 0.083 | 23.7 ± 1.1 | 55.8 | <0.001 |
| 3 | 21.0 ± 3.8 (+6.7%) |  |  | 23.6 ± 1.0 (-0.4%) |  |  |
| 5 | 21.2 ± 2.8 (+7.1%) |  |  | 174.9 ± 14.6 (+638.0%) |  |  |
| 7 | 18.7 ± 2.2 (-5.1%) |  |  | 417.0 ± 17.2 (+1659.5%) |  |  |
| 10 | 26.5 ± 2.0 (34.5%) |  |  | 449.4 ± 14.5 (+1796.2%) |  |  |
| 1-10 | 22.4 ± 1.7 |  |  | 215.3 ± 7.2 |  |  |
| 13 | 25.0 ± 2.1 (+26.9%) | 0.6 | 0.008 | 420.3 ± 13.1 (+1673.4%) | -12.9 | <0.001 |
| 16 | 26.5 ± 2.7 (+34.5%) |  |  | 332.0 ± 12.6 (+1300.8%) |  |  |
| 23 | 31.3 ± 4.1 (+58.9%) |  |  | 213.0 ± 8.9 (+798.8%) |  |  |
| 30 | 31.1 ± 5.2 (+57.9%) |  |  | 141.1 ± 7.6 (+495.4%) |  |  |
| 37 | 41.8 ± 7.2 (+112.2%) |  |  | 105.6 ± 5.9 (+345.6%) |  |  |
| 13-37 | 31.2 ± 2.1 |  |  | 242.5 ± 5.4 |  |  |
| 44 | 38.9 ± 6.3 (+97.5%) | -0.2 | 0.036 | 79.9 ± 4.2 (+237.1%) | -0.4 | <0.001 |
| 51 | 61.0 ± 5.7 (209.6%) |  |  | 89.6 ± 4.4 (+278.1%) |  |  |
| 58 | 35.6 ± 5.7 (+80.7%) |  |  | 78.1 ± 3.6 (+229.5%) |  |  |
| 65 | 37.3 ± 4.5 (+89.3%) |  |  | 61.2 ± 2.9 (+158.2%) |  |  |
| 72 | 34.9 ± 3.8 (+77.2%) |  |  | 67.5 ± 3.4 (+184.8%) |  |  |
| 86 | 34.3 ± 3.6 (+74.1%) |  |  | 63.7 ± 2.4 (+168.8%) |  |  |
| 100 | 39.3 ± 5.3 (+99.5%) |  |  | 61.5 ± 3.0 (+159.5%) |  |  |
| 114 | 32.4 ± 2.7 (+64.5%) |  |  | 60.8 ± 2.8 (+156.5%) |  |  |
| 44-114 | 39.0 ± 1.8 |  |  | 70.4 ± 1.2 |  |  |
| All | 32.3 ± 1.1 |  |  | 158.6 ± 2.8 |  |  |

**Table S3.** In vivo impedance at 1 kHz of electrodes implanted in the rat brain.

**Table S4.** Parameter estimates of the LMM for the SNR of SSVEP at 8 Hz for the aGel-μECoG (5 rats) and μECoG (5 rats) electrodes over 1-16 weeks.

| Index parameter | | Estimate | SE | confidence interval (95%) | *p* |
| --- | --- | --- | --- | --- | --- |
| Fixed Effects  (aGel-μECoG as reference group) | *β_0_* | 110.7 | 13.9 | [83.42, 137.99] | <0.001 |
|  | *β_1_* | 10.3 | 19.3 | [-27.57, 48.16] | 0.594 |
|  | *β_2_* | -0.4 | 0.2 | [-0.72, 0.04] | 0.029 |
|  | *β_3_* | -1.0 | 0.2 | [-1.46, -0.52] | <0.001 |
| Random Effects | *μ* | 30.4 |  |  |  |
|  | *ν* | 21.4 |  |  |  |
|  | *ε* | 49.6 |  |  |  |

**Table S5.** Predicted SNR at 8 Hz for aGel-μECoG (5 rats) and μECoG (5 rats) electrodes per week using LMM. Week 1 was used as the baseline for normalization.

| Week | Predicted SNR for 8 Hz (%) | |
| --- | --- | --- |
|  | aGel-μECoG | μECoG |
| 1 | 100.0 | 100.0 |
| 2 | 99.7 | 98.9 |
| 3 | 99.3 | 97.7 |
| 4 | 99.0 | 96.5 |
| 5 | 98.6 | 95.4 |
| 6 | 98.3 | 94.3 |
| 7 | 97.9 | 93.1 |
| 8 | 97.6 | 92.0 |
| 9 | 97.2 | 90.8 |
| 10 | 96.9 | 89.7 |
| 11 | 96.6 | 88.5 |
| 12 | 96.2 | 87.4 |
| 13 | 95.9 | 86.3 |
| 14 | 95.5 | 85.1 |
| 15 | 95.2 | 84.0 |
| 16 | 94.8 | 82.8 |

**Table S6.** Parameter estimates of the LMM for SSVEP SNR at 8 Hz recorded using aGel-μECoG (5 rats) electrodes over 1-16 weeks. Segmentation was performed at week 7 to accommodate separate linear trends before and after week 7 of the implantation.

| Segmentation | Index parameter | | Estimate | SE | confidence interval (95%) | *p* |
| --- | --- | --- | --- | --- | --- | --- |
| Before Week 7 | Fixed Effects | *β_0_* | 152.0 | 11.4 | [129.61, 174.44] | <0.001 |
|  |  | *β_2_* | -7.4 | 0.5 | [-8.39, -6.34] | <0.001 |
|  | Random Effects | *μ* | 22.8 |  |  |  |
|  |  | *ν* | 35.3 |  |  |  |
|  |  | *ε* | 53.3 |  |  |  |
| After Week 7 | Fixed Effects | *β_0_* | 80.0 | 11.5 | [57.50, 102.44] | <0.001 |
|  |  | *β_2_* | 2.1 | 0.4 | [1.38, -2.80] | <0.001 |
|  | Random Effects | *μ* | 23.0 |  |  |  |
|  |  | *ν* | 23.8 |  |  |  |
|  |  | *ε* | 49.5 |  |  |  |

**Table S7.** Predicted SNR at 8 Hz for μECoG (5 rats) electrodes per week using LMM, segmented at week 7. Week 1 was used as the baseline for normalization.

| Segmentation | Week | Predicted SNR for 8 Hz (%) |
| --- | --- | --- |
|  |  | μECoG |
| Before Week 7 | 1 | 100 |
|  | 2 | 94.9 |
|  | 3 | 89.8 |
|  | 4 | 84.7 |
|  | 5 | 79.6 |
|  | 6 | 74.5 |
|  | 7 | 69.5 |
| After Week 7 | 7 | 65.4 |
|  | 8 | 66.8 |
|  | 9 | 68.3 |
|  | 10 | 69.7 |
|  | 11 | 71.2 |
|  | 12 | 72.6 |
|  | 13 | 74.1 |
|  | 14 | 75.5 |
|  | 15 | 77.0 |
|  | 16 | 78.4 |

**Movie S1. Demonstration of robust adhesion and safe removal of aGel-μECoG from brain tissue.** Attachment of aGel-μECoG to the surface of rat brain tissue. Fresh, intact rat brain tissue was used for the experiments. The video shows the process of attaching aGel-μECoG in a dry state to the surface of moist rat brain tissue, followed by the application of shear and peeling forces. The aGel-μECoG spontaneously adhered to the surface of the brain tissue without pressing or additional manipulation. During the application of shear force, the aGel-μECoG maintained its initial implantation position. When subjected to a peeling force, aGel-μECoG was easily removed without damaging the brain tissue. The video playback speed was 2x. The entire process took approximately 1 min. To maintain a reasonable video size, the middle 30 s between the initial apposition and lateral tugging were omitted, during which no manipulation was performed.

**Movie S2. Electrical stimulation of the rat sciatic nerve.** Electrical stimulation of the rat sciatic nerve. The sciatic nerve of the right hind leg of each rat was exposed using scissors and tweezers. The ends of the stimulation electrodes were bent to securely hook onto the nerve. The video demonstrates electrical stimulation at 1 mA for 10 s with a stimulation frequency of 1 Hz.

**Movie S3. SSVEP recordings from head-fixed rats.** This video shows a head-fixed rat receiving 8 Hz light flashes, while the SSVEP signal was recorded by the headstage attached to its head. The rats were placed in a custom-designed jacket to hold them still during recording. The recording environment was completely dark, except for 8 Hz light flashes.
